# Reporting of flow diagrams in randomised controlled trials published in periodontology and implantology: a survey

**DOI:** 10.1186/s12874-023-01923-7

**Published:** 2023-04-27

**Authors:** Hanns-Gustav Julius Meyer, Nikolaos Pandis, Jadbinder Seehra, Clovis Mariano Faggion

**Affiliations:** 1 Department of Prosthodontics and Biomaterials, Faculty of Dentistry, University Hospital Münster, Waldeyerstraße 30, 48149 Münster Münster, Germany; 2 Department of Orthodontics and Dentofacial Orthopedics, Dental School/Medical Faculty, University of Bern, Bern, Switzerland; 3 Centre for Craniofacial Development & Regeneration, Faculty of Dentistry, Oral & Craniofacial Sciences, King’s College London, Floor 25, Guy’s Hospital, London, SE1 9RT UK; 4 Department of Periodontology and Operative Dentistry, Faculty of Dentistry, University Hospital Münster, Waldeyerstraße 30, 48149 Münster Münster, Germany

**Keywords:** Randomised controlled trials, Periodontics, Dental implants, Methods, Methodological study, Evidence-based dentistry

## Abstract

**Background:**

Item 13 of the CONSORT guidelines recommends documentation of the participant flow in randomised clinical trials (RCTs) using a diagram. In the medical literature, the reporting of the flow of participants in RCTs has been assessed to be inadequate. The quality of reporting flow diagrams in periodontology and implantology remains unknown. The aim of this study was to assess the reporting of flow diagrams in RCTs published in periodontology and implantology journals.

**Materials and Methods:**

RCTs published between 15^th^ January 2018 and 15^th^ January 2022 in twelve high-ranked periodontology and implantology journals were identified. Trial characteristics at the RCT level were extracted. The flow diagram included in each RCT was assessed for completeness of reporting in relation to published criteria and the CONSORT flow diagram template.

**Results:**

From the 544 eligible articles, 85% were single-centre, 82% of parallel-group design and 79% investigated surgical interventions. Three-hundred and fifteen (58%) articles were published in CONSORT endorsing journals. A flow diagram was reported in 317 (58%) trials and reporting was more common in periodontology (73.1%). Overall, 56% of publications with a flow diagram reported a complete CONSORT flow diagram, while in 44% of flow diagrams, at least one point from the CONSORT reporting template was missing. Reasons for loss to follow-up (69.7%) and exclusions from the RCT analysis (86.4%) were poorly reported.

**Conclusion:**

The reporting of flow diagrams in periodontology and implantology RCTs was sub-optimal. Greater awareness of the importance of fully completing the participant CONSORT flow diagram is required.

**Supplementary Information:**

The online version contains supplementary material available at 10.1186/s12874-023-01923-7.

## Background

Within healthcare, randomised controlled trials (RCTs) are considered the gold standard methodology to appraise both the efficacy and safety of medical interventions [1]. Clear and transparent reporting in the scientific literature improves the trial's reproducibility, allows for the assessment of the internal and external validity of the trial [2], reduces research waste [3] and correctly maps the certainty of the available evidence during clinical decisions. To improve the reporting of RCTs, the CONSORT reporting guideline was introduced in 1996 [4] with the current version published in 2010 and consisting of a 25-item checklist [5].

Item 13, which is in two parts, relates to the documentation of the flow of the trial participants and strongly recommends the use of flow diagrams to help visualize the trial flow and any deviations from the protocol which could potentially bias the results [5] (CONSORT Flow Diagram Template, see Additional file 1). In the flow diagram, the number of trial participants who were randomly allocated to different trial arms and received the intended intervention should be reported. In addition, losses and exclusions after randomisation, together with reasons, should be reported per trial arm [5]. The reporting of losses with reasons is important because losses to follow-up associated with the treatment or the outcome can bias the trial results [6–8]. A distinction also must be made between trial participants who are lost to follow-up (attrition) and potential study participants that were not included for reasons such as not meeting the eligibility criteria. Not meeting eligibility criteria relates only to the applicability of the trial results (external validity) and does not introduce bias.

Previous surveys report the presence of an RCT flow diagram in 20.5% to 70.6% of papers within different fields of biomedicine [9–16] and suggest that the completeness of reporting seems inadequate [12–14]. Commonly, the number of trial participants allocated to the trial arms, the number of trial participants who discontinued the intervention and the number of trial participants lost to follow-up were poorly reported [12, 13]. Up to 20% of RCTs also did not report the number of trial participants who underwent randomisation and the number of trial participants that were excluded from the statistical analyses [12]. According to recent studies, the reporting prevalence of a flow diagram in dentistry and periodontology trials was 50% and 59%, respectively [17, 18]. However, the quality of reporting of the flow diagrams in periodontology and implantology RCTs remains unknown. Therefore, the aim of this meta-research study was to assess the reporting of flow diagrams in RCTs published in periodontology and implantology journals in relation to the CONSORT flow diagram template. Factors associated with the reporting of flow diagrams versus non-reporting of flow diagrams were also investigated.

## Materials and methods

### Eligibility criteria

English language RCTs published in periodontology and implantology journals with the highest-ranked 2021 impact factors were identified [19]. RCTs with the following design were included: parallel, split-mouth, crossover, cluster, factorial and interim analysis. If an RCT had a follow-up publication, this was also included. Quasi-RCTs, non-human RCTs, non-randomised clinical trials, secondary subgroup analysis from RCTs, systematic reviews, meta-analyses, and observational studies were excluded.

### Search strategy and selection

RCTs published between 15^th^ January 2018 and 15^th^ January 2022 in twelve journals were identified from a search of a single electronic database (Medline via PubMed). The first part of the search was performed on 15th January 2021 and the second on 30th March 2022. The search criteria are reported in detail in Additional file 2. We searched this time interval to have the most updated evidence about the researched topic. The database was searched using a combination of keywords and the journals' International Standard Serial Number (ISSN). The following periodontology and implantology journals were searched: Journal of Clinical Periodontology, Journal of Periodontology, Clinical Oral Implants Research, Clinical Implant Dentistry and Related Research, Journal of Periodontal Research, International Journal of Oral Implantology, International Journal of Oral & Maxillofacial Implants, International Journal of Implant Dentistry, Journal of Periodontal and Implant Science, International Journal of Periodontics & Restorative Dentistry, Implant Dentistry and Journal of Oral Implantology.

Titles and abstracts of articles were reviewed. If the article did not meet the eligibility criteria, it was excluded and reasons for exclusion were recorded. The search and article identification were carried out independently by two reviewers (GM and CMF). Any discrepancies were resolved by discussion between all reviewers.

### Data extraction

A standardized pre-piloted data extraction spreadsheet was used to extract the data. Data extraction was carried out independently by two reviewers (GM, CMF). Both reviewers extracted data from 10% of eligible studies and achieved a good level of agreement (80%) [20]. The remaining data was extracted by one reviewer (GM). At both the journal and trial level, the following characteristics were extracted: journal type (periodontology or implantology), dental specialty (periodontology or implantology), pilot study (yes or no), trial design (parallel, split-mouth, parallel & split-mouth, cross-over and other), type of intervention (interventional or non-interventional RCT), type of procedure (surgery or non-surgery), number of centres (single or multi), number of treatment arms, published in a CONSORT endorsing journal (endorsed or non-endorsed), type of funding (non-profit, profit, self-funded, not reported), report of conflict of interest (yes or no), statistically significant results (yes or no), trial registration (yes or no), statistician involved (yes or no), country and continent of the first author, report of ethical approval (yes or no), report of a flow diagram (yes or no) and if yes, is it in the main text or in supplementary files, RCT analysis type (intention-to-treat, per protocol, intention-to-treat and per protocol, not reported), number of authors, sample size. Additionally, any deviations from the reported analysis type and the actual analysis conducted were assessed.

Each RCT flow diagram was assessed for discrepancies against the CONSORT flow diagram template (Supplementary file, Fig. 1). Additionally, the completeness of the flow diagram was assessed in relation to the criteria described by Hopewell [13]. This consists of 17 items that assesses the reporting of four domains: enrollment, allocation, follow-up and analysis. The reporting of each item was categorised as either reported or not reported. Studies reporting dropouts and studies with no dropouts were considered as `reported´ in the lost to follow-up and discontinued intervention stage. The number of studies reporting dropouts was also recorded.

**Fig. 1:**
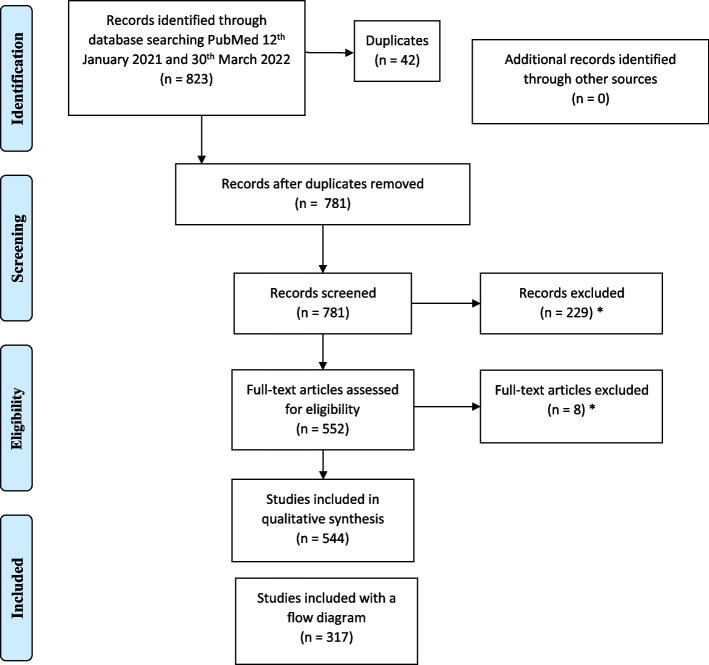
PRISMA flow diagram Legend: * Reasons for exclusion are reported in the Additional files 4 and 5

### Statistical analysis

Descriptive and frequency statistics were calculated for both the trial characteristics and for the reporting adherence against the CONSORT flow diagram template and published criteria [13]. A Pearson´s chi-squared test was performed to detect any associations between the reporting of a flow diagram versus non-reporting of flow diagrams and RCT characteristics. A two-tailed *P*-value of 0.05 was considered statistically significant. All analyses were performed using Stata 17 (Stata Corp, Texas, USA) and R Software version 4.0.3 (R Foundation for Statistical Computing, Vienna, Austria).

## Results

### Selection process

Eight hundred and twenty-three articles were identified in the primary search with forty-two duplicates. After reviewing the abstract and title, two hundred and twenty-nine articles were excluded. After the full-text assessment, a further eight articles were excluded. Five Hundred and forty-four articles (from 541 RCTs) were included in the final analysis (Fig. 1). Three articles were a follow-up of an RCT and reported results from different time periods. The list of included articles and excluded articles with reasons for exclusion are reported in the Additional files 3, 4 and 5.

### Journal characteristics (*N* = 544)

From the twelve journals included in this assessment, Clinical Oral Implants Research, Journal of Clinical Periodontology, Journal of Periodontal Research and Journal of Periodontology endorse the CONSORT recommendations according to the CONSORT website. The majority of RCTs were published in Clinical Oral Implants Research (*n* = 108/544, 20%), Journal of Clinical Periodontology (*n* = 106/544, 19%), Journal of Periodontology (*n* = 86/544,16%) and Clinical Implant Dentistry Related Research (*n* = 82/544, 15%). The median IF was 4.494 with the highest IF from Journal of Clinical Periodontology (7.478) and the lowest IF from Journal of Oral Implantology (1.546) (Table 1).

**Table 1:** Journal characteristics (*N* = 544)

Journals of included RCTs	Publisher	n	%	CONSORT endorsing
Clinical Implant Dentistry Related Research	Wiley	82	15%	no
Clinical Oral Implants Research	Wiley	108	20%	yes
International Journal of Oral Implantology^a^	Quintessence Publishing	20	4%	no
Implant Dentistry	Wolters Kluwer	8	1%	no
International Journal of Implant Dentistry	Springer	20	4%	no
International Journal of Oral & Maxillofacial Implants	Quintessence Publishing	37	7%	no
International Journal of Periodontics & Restorative Dentistry	Quintessence Publishing	37	7%	no
Journal of Clinical Periodontology	Wiley	106	19%	yes
Journal of Oral Implantology	Allen Press	14	3%	no
Journal of Periodontal Research	Wiley	15	3%	yes
Journal of Periodontal & Implant Science	Korean Academy of Periodontology	11	2%	no
Journal of Periodontology	Wiley	86	16%	yes
Characteristics		**Median**	**IQR**	
Impact factor		4.494	*1.898*	

^a^European Journal of Oral Implantology was renamed to International Journal of Oral Implantology in 2019

### Article characteristics (*N* = 544)

Fifty-three percent (*n* = 286/544) of trials were published in a journal related to implantology. The most common trial design was parallel (*n* = 444/544, 82%), followed by split-mouth (*n* = 84/544, 15%) and 26/544 (5%) articles were pilot studies. More trials were funded by for-profit organizations (*n* = 221/544, 41%) than by non-profit organizations (*n* = 174/544, 32%) and more than half of the RCTs were registered either at Clinicaltrials.gov or in national databases for RCTs (*n* = 316/544, 58%). A flow diagram was reported in 58% (*n* = 317/544) of articles reporting trials and was commonly located in the main text (*n* = 294/317, 93%) (Table 2).

**Table 2:** Article Characteristics (*N* = 544)

Characteristics	n	%
**Journal**		
Periodontology	258	*47%*
Implantology	286	*53%*
**Dental specialty**		
Periodontology	171	*31%*
Implantology	373	*69%*
**Pilot Study**		
Yes	26	*5%*
No	518	*95%*
**Design**		
Parallel	444	*82%*
Split-mouth	84	*15%*
Crossover	9	*2%*
Other	4	*1%*
Parallel and split-mouth	3	*1%*
**Type of intervention**		
Interventional RCT	544	*100%*
**Type of procedure**		
Surgical	431	*79%*
Non-surgical	113	*21%*
**Centre**		
Single-centre	464	*85%*
Multi-centre	80	*15%*
**Number of treatment arms**		
2	464	*85%*
3	59	*11%*
4	19	*3%*
5	1	*0.5%*
8	1	*0.5%*
**CONSORT endorsement**		
Yes	315	*58%*
No	229	*42%*
**Funding**		
Non-profit organization	174	*32%*
Profit organization	221	*41%*
Self-funded	45	*8%*
Not reported	104	*19%*
**Conflict of interest**		
Reported	516	*95%*
Not reported	28	*5%*
**Statistical results**		
Significant results	432	*79%*
Non-significant results	112	*21%*
**Registration**		
Registered	316	*58%*
Not registered	228	*42%*
**Statistician**		
Statistician involved	115	*21%*
Statistician not involved	429	*79%*
**Country**		
USA	63	*12%*
Italy	59	*11%*
Brasil	54	*10%*
Switzerland	33	*6%*
Spain	33	*6%*
China	30	*6%*
Germany	28	*5%*
Turkey	23	*4%*
Egypt	22	*4%*
Netherlands	19	*3%*
**Top 10 countries**	364	*67%*
**Remaining countries**	180	*33%*
**Continent**		
Africa	27	*5%*
Asia	134	*25%*
Australia	4	*1%*
Europe	255	*47%*
North America	67	*12%*
South America	57	*10%*
**Ethics board approval**		
Reported	526	*97%*
Not reported	18	*3%*
**Flow diagram**		
Reported	317	*58%*
Not reported	227	*42%*
**Flow diagram (where reported)**		
Main text	294	93%
Additional information	23	7%
**Type of analysis**		
Intention-to-treat	54	*10%*
Per Protocol	14	*3%*
Not reported	460	*84%*
Intention-to-treat and per protocol	16	*3%*
**Characteristics**	**Median**	**IQR**
Number of authors	6	2
Sample size	36	29

### Reporting of article flow diagrams against published criteria and CONSORT flow diagram template (*N* = 317)

**Fig. 2:**
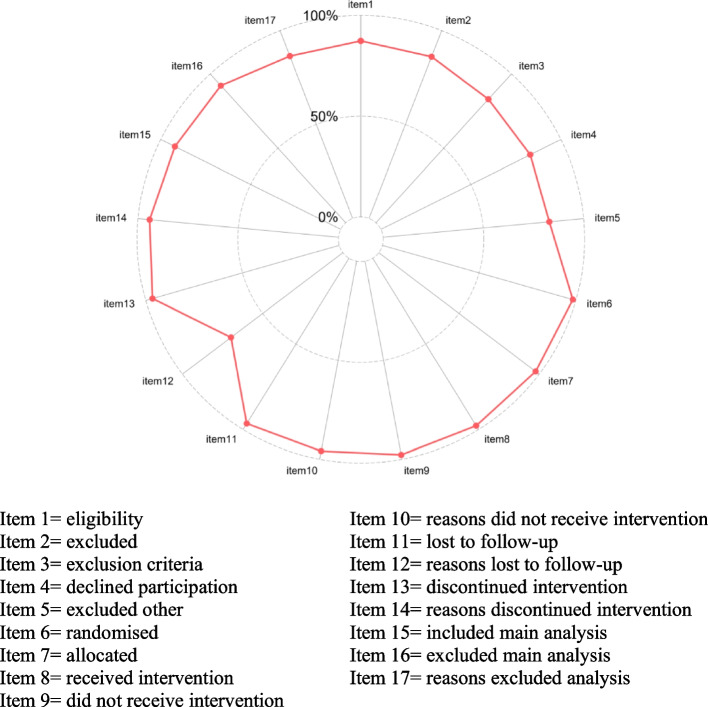
Radial plot showing completeness of reporting per item in the flow diagram (*N* = 317)

The reporting adherence of each flow diagram against published criteria is shown in Fig. 2 and Additional file 6. From the CONSORT endorsing journals (*n* = 315), 225 (71%) included a flow diagram, and from the non-CONSORT endorsing journals (*n* = 229), 92 (40%) included a flow diagram (Table 3). Overall, just over half of the articles (*n* = 178/317, 56.2%) reported a complete flow diagram, while at 43.8% (*n* = 139/317) of the articles at least one item from the CONSORT reporting template was missing. There was variability in the completeness of reporting among flow diagram items. Trial eligibility (*n* = 277/317, 87.4%), number of excluded participants (*n* = 273/317, 86.1%), number of participants randomised (*n* = 312/317, 98.4%), and participant group and intervention allocation (*n* = 310/317, 97.8%) and reasons for discontinued interventions (*n* = 299/317, 94.3%) were relatively well reported. However, reasons for losses to follow-up were missing in a third (*n* = 96/317, 30.3%) of article flow diagrams.

**Table 3:** Associations between the reporting of a flow diagram versus non-reporting of flow diagrams and journal and RCT characteristics

Characteristics	*N* = 317 Flow diagram reported (%)	*N* = 227 Flow diagram not reported (%)	*P* value
Periodontology	125 (73.1)	46 (26.9)	< 0.001
Implantology	192 (51.5)	181 (48.5)	
Parallel	267 (60.1)	177 (39.9)	0.12
Split-mouth	42 (48.3)	45 (51.7)	
Other	8 (61.5)	5 (38.5)	
Surgical	229 (53.1)	202 (46.9)	< 0.001
Non surgical	88 (77.9)	25 (22.1)	
Single-centre	272 (58.6)	192 (41.4)	0.69
Multi-centre	45 (56.3)	35 (43.8)	
CONSORT endorsed	225 (71.4)	90 (28.6)	< 0.001
Non endorsed	92 (40.2)	137 (59.8)	
Non-profit	122 (70.1)	52 (29.9)	< 0.001
Profit	128 (57.9)	93 (42.1)	
No sponsor	24 (53.3)	21 (46.7)	
Unclear funding	43 (41.3)	61 (58.7)	
Conflict of interest reported	305 (59.1)	211 (40.9)	0.09
Not reported	12 (42.9)	16 (57.1)	
Statistical significant results	255 (59.0)	177 (41.0)	0.48
Non significant results	62 (55.4)	50 (44.6)	
Trial registered	217 (68.7)	99 (31.3)	< 0.001
Not registered	100 (43.9)	128 (56.1)	
Statistician involved	71 (61.7)	44 (38.3)	0.40
Not involved	246 (57.3)	183 (42.7)	
Africa	9 (33.3)	18 (66.7)	< 0.001
Asia & Australia	94 (68.1)	44 (31.9)	
Europe	136 (53.3)	119 (46.7)	
North America	33 (49.3)	34 (50.7)	
South America	45 (78.9)	12 (21.1)	
Ethical approval reported	315 (59.9)	211 (40.1)	< 0.001
Not reported	2 (11.1)	16 (88.9)	

### Associations between reporting of a flow diagram versus non-reporting of flow diagrams and article characteristics

An association between the reporting of a flow diagram versus non-reporting of flow diagrams and the following journal and article characteristics was evident: dental specialty (*p* < 0.001), type of procedure (*p* < 0.001), CONSORT endorsing journal (*p* < 0.001), type of funding (*p* < 0.001), trial registration (*p* < 0.001), country and continent of the first author (*p* < 0.001) and report of ethical approval (*p* < 0.001) (Table 3).

### Type of analysis

Within the total sample of articles (*n* = 544), the majority (*n* = 460/544, 84.6%) did not disclose whether the analysis followed the intention-to-treat principle or per protocol. From 317 articles reporting a flow diagram, 210 (66.2%) had participant dropouts from the studies. Within this cohort, 35/210 (16.7%) trials reported an intention-to-treat analysis and out of those 9/35 (25.7%) reported the use of the last observation carried forward approach and 8/35 (22.9%) the imputation of missing data without further explanation. The remaining 18/35 (51.45%) reported an intention-to-treat analysis, but in fact a per-protocol analysis was conducted. Only 7/210 trials (3.3%) performed a per-protocol analysis and 9/210 (4.3%) trials combined an intention-to-treat principle and per-protocol analysis. One-hundred and fifty-nine out of 210 (75.7%) trials with missing data did not provide information on the type of analysis and the handling of missing data (Table 4).

**Table 4:** Type of analysis used in randomised controlled trials

Randomised controlled trial Type of analysis	Deviation from reporting and actual type of analysis	No deviation from reporting and actual type of analysis	Total
Flow diagram included with missing data			
Intention-to-treat	18	17	35
Per-protocol	0	7	7
Intention-to-treat & per-protocol	0	9	9
Not reported	159	0	159
			210
Flow diagram included without missing data			
Intention-to-treat	5	0	5
Per-protocol	0	0	0
Intention-to-treat & per-protocol	0	2	2
Not reported	100	0	100
			107
All articles			
Intention-to-treat	23	31	54
Per-protocol	14	0	14
Intention-to-treat & per-protocol	0	16	16
Not reported	460	0	460
			544

## Discussion

Within this study sample, 58% (*n* = 315/544) of articles were published in journals that endorse the CONSORT recommendations and therefore, it is not surprising that the majority of these articles (*n* = 225/315, 71.4%) included a flow diagram. The reason that not all reports in the CONSORT endorsing journals included a flow diagram could be attributed to the fact that the instructions to authors can vary from recommended to required [21].

In the current sample, only 32,7% (*n* = 178/544) of articles reported a fully completed flow diagram. The reporting of reasons lost to follow-up and exclusion from the analysis were commonly not reported. As previously reported, clarification of the reasons for dropouts is important as dropouts can bias the treatment results [7]. In the presence of dropouts, a per-protocol analysis could give rise to bias if the remaining patients in the trial and the lost patients have different baseline characteristics or different prognoses. An intention-to-treat analysis with imputation of missing data is an approach to consider and compare with the per-protocol analysis.

In our study, 84,6% (*n* = 460/544) of the publications did not report the type of analysis (intention-to-treat or per-protocol). These findings are in agreement with another meta-research study on the reporting and handling of incomplete outcome data in implantology that was recently published where 85% of the RCTs did not report the type of analysis [22]. In the present study, eight from 35 studies with dropouts and using intention-to-treat analysis did not explain how missing data was treated. An assumption is that the authors ignored the missing data and used a “complete case analysis” [23]. This approach can be problematic because it makes the strong assumption that the data is missing completely at random which would hypothetically not affect the treatment effect estimates. In the previously mentioned study that assessed implantology RCTs, it was assumed by the authors of the study that the complete case analysis approach was applied in 45% of the RCTs [22]. In addition, there is often confusion as to what each analytical approach means, and the different approaches have been used under the same name [24].

Possible reasons for omitting the flow diagram could be due to manuscript space limitations and the limitations on the number of tables and figures that can be included. However, in the current investigation, 93% (*n* = 294/317) of the flow diagrams were in the main text and only 7% (*n* = 23/317) were in the supplementary files. Three out of four CONSORT endorsing journals are related to periodontology. This could explain the observation that reporting of flow diagrams was more frequent in periodontology publications. Registered studies and studies including ethical approval were more likely to report a flow diagram. These studies usually have a research protocol to be approved by the ethics committee and one can assume that the authors of these studies are more compliant with reporting standards.

The reporting of a flow diagram has been assessed in previous studies from several areas of medicine and varied widely from 20.5%-70.6% [9–16]. Two recent studies in periodontology and general dentistry reported 50% and 59% compliance with flow diagram reporting [17, 18]; a finding consistent with our results. In comparison, a study assessing trials published between 2007–2012 in implantology and prosthodontics reported only 23% compliance with flow diagram reporting [25]. These studies reported only the presence or absence of a flow diagram with no assessment of the quality of the information reported within the flow diagram. Our results are broadly similar to those of Hopewell et al. in that the reporting of reasons lost to follow-up and analysis exclusion were infrequently reported [13]. More recent investigations conducted in pharmacology reported that the enrollment, randomisation, allocation and analysis are well documented (78%-96%), however, the follow-up section is underreported (44%-61%) [15]. In that study, the reasons for `lost to follow-up´ and `excluded from analysis´ were not assessed and no distinction was made between `lost to follow-up´ and `discontinued intervention´.

Five hundred and forty-four articles (from 541 RCTs) were included in this study which is a large enough sample to examine the reporting quality of RCT flow diagrams published in periodontology and implantology journals. Only high impact journals were included in this assessment and hence a degree of bias (probably over estimation of flow diagram reporting) may exist as the results are only representative of these journals. However, we hypothesize that lower ranked journals in both specialties are less likely to include flow diagrams and to have them properly completed. For the studies without a reported flow diagram, we did not extract the information from the text that should be presented in the flow diagram according to CONSORT recommendations. The review of the text was beyond the remit of this study but could be explored in future studies.

Flow diagrams shorten the time it takes to extract essential information that facilitates the assessment of the study quality. Hopewell et al. found that in a sample of 469 RCTs on health care interventions, only 50% of RCTs reported the number of participants included in the main analysis [13]. However, in most of these trials, the information on the number of participants was reported in the full text and tables and figures. Depending on the reader’s experience, it took, on average, six minutes to find this extra information in text, tables and figures [13]. Considering that there has been a significant growth in dental publications as well as in implantology [26–28], a quick understanding of a study can help systematic reviews and clinical guidelines developers better deal with the increasing amount of published research.

In conclusion, under 60% (*n* = 317/544) of articles included a participant flow diagram of which 56.2% (*n* = 178/317) reported a complete CONSORT flow diagram. Only 178/544 articles (32.7%) from our sample followed the CONSORT recommendations on item 13 sufficiently. The reporting of reasons lost to follow-up and analysis exclusion was sub-optimal. To improve transparency and to facilitate quality assessment of published RCTs an accurate and complete participant CONSORT flow diagram should be included in trial manuscripts. Greater awareness of the importance of fully completing the participant CONSORT flow diagram is required.

## Supplementary Information

**Additional file 1. **CONSORT Flow Diagram Template.

**Additional file 2. **Literature search.

**Additional file 3. **List of included articles.

**Additional file 4. **Articles excluded after abstract assessment with reasons.

**Additional file 5. **Articles excluded after full text assessment with reasons.

**Additional file 6. **Completeness of reporting per item in the flow diagram (*N*=317).

**Additional file 7. **Raw data.

## Data Availability

The dataset generated and analysed during this study is reported as an excel sheet in this published article (see Additional file 7).
