# Supplementary figures and images for: Reporting of flow diagrams in randomised controlled trials published in periodontology and implantology: a survey
Source: BMC Med Res Methodol. 2023 Apr 27;23:105. doi: 10.1186/s12874-023-01923-7 (PMC10134555; doi:10.1186/s12874-023-01923-7)

**Additional file 1** CONSORT Flow Diagram Template


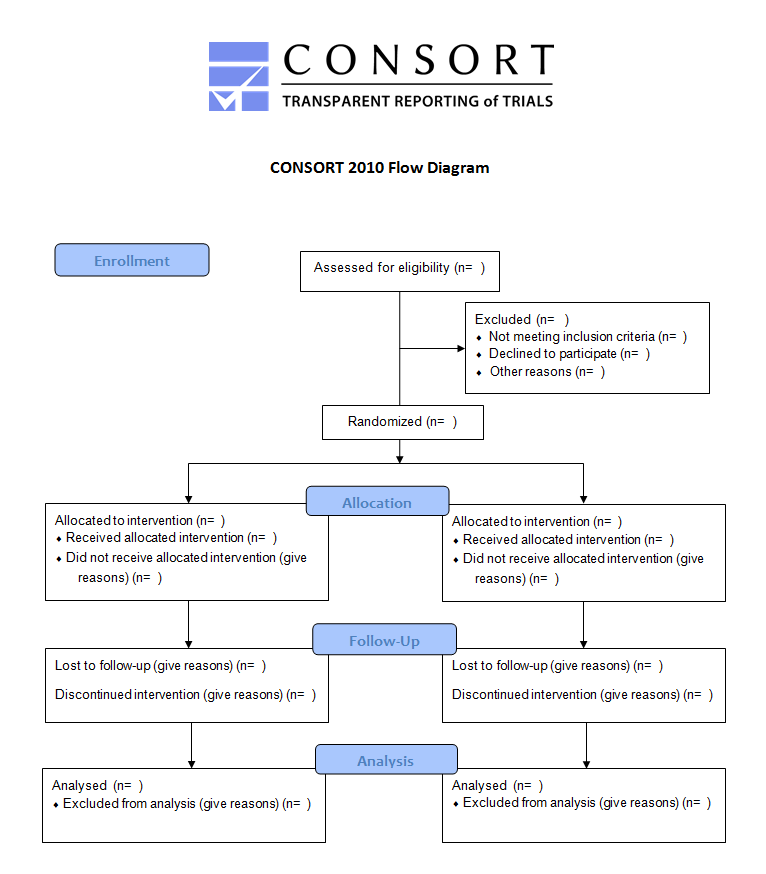

Supplement: Supplementary file 1 — Additional file 1. CONSORT Flow Diagram Template. [file 12874_2023_1923_MOESM1_ESM.docx]
