# Supplementary material for: Reporting of flow diagrams in randomised controlled trials published in periodontology and implantology: a survey
Source: BMC Med Res Methodol. 2023 Apr 27;23:105. doi: 10.1186/s12874-023-01923-7 (PMC10134555; doi:10.1186/s12874-023-01923-7)
Supplement: Supplementary file 2 — Additional file 2. Literature search. [file 12874_2023_1923_MOESM2_ESM.docx]

**Additional file 2**

**Search PubMed 12th January 2021**

#6 Search: #1 AND #2 Filters: in the last 1 year Sort by: Most Recent

221 03:27:56

#5 Search: #1 AND #2 Filters: in the last 5 years Sort by: Most Recent

1,006 03:27:52

#4 Search: #1 AND #2 Filters: in the last 10 years Sort by: Most Recent

1,791 03:27:45

#3 Search: #1 AND #2 Sort by: Most Recent

2,734 03:27:38

#2 Search: randomised[Title/Abstract] OR randomized[Title/Abstract] Sort by: Most Recent

653,327 03:27:16

#1 Search: 1600-051X OR 1943-3670 OR 1600-0501 OR 1708-8208 OR 1600-0765 OR 1756-2414 OR 1942-4434 OR 2198-4034 OR 2093-2286 OR 1945-3388 OR 1056-6163 OR 1548-1336 Sort by: Most Recent

36,545 03:26:48

**Updated Search PubMed 30th March 2022**

| **Search number** | **Query** | **Sort By** | **Filters** | **Search Details** | **Results** | **Time** |
| --- | --- | --- | --- | --- | --- | --- |
| **4** | #1 AND #2 | Most Recent | from 2021/1/12 - 2022/1/12 | (("j clin periodontol"[Journal] OR "j periodontol"[Journal] OR "clin oral implants res"[Journal] OR "clin implant dent relat res"[Journal] OR "j periodontal res"[Journal] OR "eur j oral implantol"[Journal] OR "int j oral maxillofac implants"[Journal] OR "int j implant dent"[Journal] OR "j periodontal implant sci"[Journal] OR "int j periodontics restorative dent"[Journal] OR "implant dent"[Journal] OR "j oral implantol"[Journal]) AND ("randomised"[Title/Abstract] OR "randomized"[Title/Abstract])) AND (2021/1/12:2022/1/12[pdat]) | 238 | 15:37:57 |
| **3** | #1 AND #2 | Most Recent |  | ("j clin periodontol"[Journal] OR "j periodontol"[Journal] OR "clin oral implants res"[Journal] OR "clin implant dent relat res"[Journal] OR "j periodontal res"[Journal] OR "eur j oral implantol"[Journal] OR "int j oral maxillofac implants"[Journal] OR "int j implant dent"[Journal] OR "j periodontal implant sci"[Journal] OR "int j periodontics restorative dent"[Journal] OR "implant dent"[Journal] OR "j oral implantol"[Journal]) AND ("randomised"[Title/Abstract] OR "randomized"[Title/Abstract]) | 2,968 | 15:36:46 |
| **2** | randomised[Title/Abstract] OR randomized[Title/Abstract] | Most Recent |  | "randomised"[Title/Abstract] OR "randomized"[Title/Abstract] | 720,235 | 15:36:19 |
| **1** | 1600-051X OR 1943-3670 OR 1600-0501 OR 1708-8208 OR 1600-0765 OR 1756-2414 OR 1942-4434 OR 2198-4034 OR 2093-2286 OR 1945-3388 OR 1056-6163 OR 1548-1336 | Most Recent |  | "j clin periodontol"[Journal] OR "j periodontol"[Journal] OR "clin oral implants res"[Journal] OR "clin implant dent relat res"[Journal] OR "j periodontal res"[Journal] OR "eur j oral implantol"[Journal] OR "int j oral maxillofac implants"[Journal] OR "int j implant dent"[Journal] OR "j periodontal implant sci"[Journal] OR "int j periodontics restorative dent"[Journal] OR "implant dent"[Journal] OR "j oral implantol"[Journal] | 37,884 | 15:35:29 |
