# Supplementary material for: Reporting of flow diagrams in randomised controlled trials published in periodontology and implantology: a survey
Source: BMC Med Res Methodol. 2023 Apr 27;23:105. doi: 10.1186/s12874-023-01923-7 (PMC10134555; doi:10.1186/s12874-023-01923-7)
Supplement: Supplementary file 3 — Additional file 3. List of included articles. [file 12874_2023_1923_MOESM3_ESM.docx]

**Additional file 3, List of included articles**

1. de Resende GP, Jordão LMR, de Souza JAC, Schimmel M, Leles CR. Single versus two-implant mandibular overdentures using early-loaded titanium-zirconium implants with hydrophilic surface and ball attachments: 1-year randomized clinical trial. Clin Oral Implants Res. 2021;32:359–68.

2. Bushahri A, Kripfgans OD, George F, Wang I-C, Wang H-L, Chan H-L. Facial mucosal level of single immediately placed implants with either immediate provisionalization or delayed restoration: An intermediate-term study. J Periodontol. 2021. https://doi.org/10.1002/JPER.20-0746.

3. Özcan E, Saygun I, Kantarcı A, Özarslantürk S, Serdar MA, Özgürtaş T. The effects of a novel non-invasive application of platelet-rich fibrin on periodontal clinical parameters and gingival crevicular fluid transforming growth factor-β and collagen-1 levels: A randomized controlled clinical study. J Periodontol. 2020. https://doi.org/10.1002/JPER.20-0713.

4. McGuire MK, Scheyer ET, Lipton DI, Gunsolley JC. Randomized, controlled clinical trial to evaluate a xenogeneic collagen matrix as an alternative to free gingival grafting for oral soft tissue augmentation: 6 to 8 year follow-up. J Periodontol. 2020. https://doi.org/10.1002/JPER.20-0627.

5. Kaur M, Geurs NC, Cobb CM, Otomo-Corgel J, Takesh T, Lee JH, et al. Evaluating efficacy of a novel dentifrice in reducing probing depths in stage I and II periodontitis maintenance patients: A randomized, double-blind, positive controlled clinical trial. J Periodontol. 2020. https://doi.org/10.1002/JPER.20-0721.

6. Ben Amara H, Kim J-J, Kim H-Y, Lee J, Song H-Y, Koo K-T. Is ridge preservation effective in the extraction sockets of periodontally compromised teeth? A randomized controlled trial. J Clin Periodontol. 2021;48:464–77.

7. Camarda AJ, Durand R, Benkarim M, Rompré PH, Guertin G, Ciaburro H. Prospective randomized clinical trial evaluating the effects of two different implant collar designs on peri-implant healing and functional osseointegration after 25 years. Clin Oral Implants Res. 2021;32:285–96.

8. Velasco-Ortega E, Valente NA, Iezzi G, Petrini M, Derchi G, Barone A. Maxillary sinus augmentation with three different biomaterials: Histological, histomorphometric, clinical, and patient-reported outcomes from a randomized controlled trial. Clin Implant Dent Relat Res. 2021;23:86–95.

9. Bienz SP, Hilbe M, Hüsler J, Thoma DS, Hämmerle CHF, Jung RE. Clinical and histological comparison of the soft tissue morphology between zirconia and titanium dental implants under healthy and experimental mucositis conditions-A randomized controlled clinical trial. J Clin Periodontol. 2021;48:721–33.

10. Mihali S, Wang H-L, Karancsi O, Bratu EA. Internal hexagon versus conical implant-abutment connections: evaluation of 3-year postloading outcomes. J Oral Implantol. 2020. https://doi.org/10.1563/aaid-joi-D-19-00160.

11. Kern M, Behrendt C, Fritzer E, Kohal RJ, Luthardt RG, Maltzahn NFV, et al. 5-year randomized multicenter clinical trial on single dental implants placed in the midline of the edentulous mandible. Clin Oral Implants Res. 2021;32:212–21.

12. Wongpairojpanich J, Kijartorn P, Suwanprateeb J, Buranawat B. Effectiveness of bilayer porous polyethylene membrane for alveolar ridge preservation: A randomized controlled trial. Clin Implant Dent Relat Res. 2021;23:73–85.

13. Borges T, Montero J, Leitão B, Pereira M, Galindo-Moreno P. Periimplant bone changes in different abutment heights and insertion timing in posterior mandibular areas: Three-year results from a randomized prospective clinical trial. Clin Oral Implants Res. 2021;32:203–11.

14. Zhou Y, Shi Y, Si M, Wu M, Xie Z. The comparative evaluation of transcrestal and lateral sinus floor elevation in sites with residual bone height ≤6 mm: A two-year prospective randomized study. Clin Oral Implants Res. 2021;32:180–91.

15. Jonker BP, Gil A, Naenni N, Jung RE, Wolvius EB, Pijpe J. Soft tissue contour and radiographic evaluation of ridge preservation in early implant placement: A randomized controlled clinical trial. Clin Oral Implants Res. 2021;32:123–33.

16. Galindo-Moreno P, Gutierrez-Garrido L, Lopez-Chaichio L, Guerra-Lorenzo C, Rodriguez-Alvarez R, Padial-Molina M. Crestal bone changes around early vs. conventionally loaded implants with a multi-phosphonate coated surface: A randomized pilot clinical trial. Clin Oral Implants Res. 2021;32:75–87.

17. Zhang W, Wang W, Chu C, Jing J, Yao NA, Sun Q, et al. Clinical, inflammatory and microbiological outcomes of full-mouth scaling with adjunctive glycine powder air-polishing: A randomized trial. J Clin Periodontol. 2021;48:389–99.

18. Zazou N, Diab N, Bahaa S, El Arab AE, Aziz OA, El Nahass H. Clinical comparison of different flap advancement techniques to periosteal releasing incision in guided bone regeneration: A randomized controlled trial. Clin Implant Dent Relat Res. 2021;23:107–16.

19. Stefanelli LV, Graziani U, Pranno N, Di Carlo S, Mandelaris GA. Accuracy of Dynamic Navigation Surgery in the Placement of Pterygoid Implants. Int J Periodontics Restorative Dent. 2020;40:825–34.

20. Schinini G, Sales D, Gómez MV, Romanelli HJ, Chambrone L. Healing of donor sites of connective tissue grafts harvested by the single incision technique: A randomized clinical trial evaluating the use of collagen hemostatic sponge with or without sutures. J Periodontol. 2021;92:629–36.

21. Machtei EE, Romanos G, Kang P, Travan S, Schmidt S, Papathanasiou E, et al. Repeated delivery of chlorhexidine chips for the treatment of peri-implantitis: A multicenter, randomized, comparative clinical trial. J Periodontol. 2021;92:11–20.

22. Stumbras A, Galindo-Moreno P, Januzis G, Juodzbalys G. Three-dimensional analysis of dimensional changes after alveolar ridge preservation with bone substitutes or plasma rich in growth factors: Randomized and controlled clinical trial. Clin Implant Dent Relat Res. 2021;23:96–106.

23. Moroi A, Saito Y, Takayama A, Ueki K. Comparison of nonself-tapping tapered implant and self-tapping hybrid implant in terms of implant stability at initial and second fixation: A prospective randomized clinical trial. Clin Implant Dent Relat Res. 2020;22:679–88.

24. Faraj SA, Kutkut A, Taylor R, Villasante-Tezanos A, Huja S, Dawson D, et al. Comparison of Dehydrated Human Amnion-Chorion and Type 1 Bovine Collagen Membranes in Alveolar Ridge Preservation: A Clinical and Histological Study. J Oral Implantol. 2020. https://doi.org/10.1563/aaid-joi-D-19-00335.

25. Aoki H, Bizenjima T, Seshima F, Sato M, Irokawa D, Yoshikawa K, et al. Periodontal surgery using rhFGF-2 with deproteinized bovine bone mineral or rhFGF-2 alone: 2-year follow-up of a randomized controlled trial. J Clin Periodontol. 2021;48:91–9.

26. Guljé FL, Meijer HJA, Abrahamsson I, Barwacz CA, Chen S, Palmer PJ, et al. Comparison of 6-mm and 11-mm dental implants in the posterior region supporting fixed dental prostheses: 5-year results of an open multicenter randomized controlled trial. Clin Oral Implants Res. 2021;32:15–22.

27. Cucchi A, Vignudelli E, Fiorino A, Pellegrino G, Corinaldesi G. Vertical ridge augmentation (VRA) with Ti-reinforced d-PTFE membranes or Ti meshes and collagen membranes: 1-year results of a randomized clinical trial. Clin Oral Implants Res. 2021;32:1–14.

28. González-Martín O, Carbajo G, Rodrigo M, Montero E, Sanz M. One- versus two-stage crown lengthening surgical procedure for aesthetic restorative purposes: A randomized controlled trial. J Clin Periodontol. 2020;47:1511–21.

29. Zangrando MSR, Eustachio RR, de Rezende MLR, Sant’ana ACP, Damante CA, Greghi SLA. Clinical and patient-centered outcomes using two types of subepithelial connective tissue grafts: A split-mouth randomized clinical trial. J Periodontol. 2020. https://doi.org/10.1002/JPER.19-0646.

30. Schallhorn RA, McClain PK, Benhamou V, Doobrow JH, Grandin HM, Kasaj A. Application of enamel matrix derivative in conjunction with non-surgical therapy for treatment of moderate to severe periodontitis: A 12-month, randomized prospective, multicenter study. J Periodontol. 2021;92:619–28.

31. Saito H, Couso-Queiruga E, Shiau HJ, Stuhr S, Prasad H, Allareddy TV, et al. Evaluation of poly lactic-co-glycolic acid-coated β-tricalcium phosphate for alveolar ridge preservation: A multicenter randomized controlled trial. J Periodontol. 2021;92:524–35.

32. Kraus RD, Stricker A, Thoma DS, Jung RE. Sinus Floor Elevation with Biphasic Calcium Phosphate or Deproteinized Bovine Bone Mineral: Clinical and Histomorphometric Outcomes of a Randomized Controlled Clinical Trial. Int J Oral Maxillofac Implants. 2020;35:1005–12.

33. Merli M, Nieri M, Mariotti G, Merli M, Franchi L, Quiroga Souki B. The fence technique: Autogenous bone graft versus 50% deproteinized bovine bone matrix / 50% autogenous bone graft-A clinical double-blind randomized controlled trial. Clin Oral Implants Res. 2020;31:1223–31.

34. Srinivasan M, Schimmel M, Buser R, Maniewicz S, Herrmann FR, Müller F. Mandibular two-implant overdentures with CAD-CAM milled bars with distal extensions or retentive anchors: A randomized controlled trial. Clin Oral Implants Res. 2020;31:1207–22.

35. Donos N, Suvan JE, Calciolari E, Nibali L, Rollnick S. The effect of a behavioural management tool in adults with mild to moderate periodontitis. A single-blind, randomized controlled trial. J Periodontal Res. 2021;56:46–57.

36. Yimarj P, Subbalekha K, Dhanesuan K, Siriwatana K, Mattheos N, Pimkhaokham A. Comparison of the accuracy of implant position for two-implants supported fixed dental prosthesis using static and dynamic computer-assisted implant surgery: A randomized controlled clinical trial. Clin Implant Dent Relat Res. 2020;22:672–8.

37. Bevilacqua L, Fonzar A, Olivier S, De Biasi M, Visintin M, Angerame D, et al. Outcome of Different Surgical Approaches in the Treatment of Class II Furcation Defects in Mandibular Molars: A Randomized Clinical Trial. Int J Periodontics Restorative Dent. 2020;40:693–701.

38. Al-Hezaimi K, Naghshbandi J, Alhuzaimi R, Alonaizan F, AlQwizany I, Rotstein I. Evaluation of Recombinant Human Platelet-Derived Growth Factor or Enamel Matrix Derivative Plus Calcium Hydroxide for Pulp Capping: A Randomized Controlled Human Clinical Trial. Int J Periodontics Restorative Dent. 2020;40:645–54.

39. Zuiderveld EG, van Nimwegen WG, Meijer HJA, Jung RE, Mühlemann S, Vissink A, et al. Effect of connective tissue grafting on buccal bone changes based on cone beam computed tomography scans in the esthetic zone of single immediate implants: A 1-year randomized controlled trial. J Periodontol. 2021;92:553–61.

40. Cruz DF da, Duarte PM, Figueiredo LC, da Silva HDP, Retamal-Valdes B, Feres M, et al. Metronidazole and amoxicillin for patients with periodontitis and diabetes mellitus: 5-year secondary analysis of a randomized controlled trial. J Periodontol. 2021;92:479–87.

41. Hashemipoor M, Asghari N, Mohammadi M, Kalantari M, Arabsolghar M, Ranjbar H. Radiological and histological evaluation of horizontal ridge augmentation using corticocancellous freeze-dried bone allograft with and without autogenous bone: A randomized controlled clinical trial. Clin Implant Dent Relat Res. 2020;22:582–92.

42. Lee J-H, Jeong S-N. Effect of enamel matrix derivative on alveolar ridge preservation in the posterior maxilla: A randomized controlled clinical trial. Clin Implant Dent Relat Res. 2020;22:622–30.

43. Noelken R, Pausch T, Wagner W, Al-Nawas B. Peri-implant defect grafting with autogenous bone or bone graft material in immediate implant placement in molar extraction sites-1- to 3-year results of a prospective randomized study. Clin Oral Implants Res. 2020;31:1138–48.

44. Kraft B, Frizzera F, de Freitas RM, de Oliveira GJLP, Marcantonio Junior E. Impact of fully or partially guided surgery on the position of single implants immediately placed in maxillary incisor sockets: A randomized controlled clinical trial. Clin Implant Dent Relat Res. 2020;22:631–7.

45. Jung RE, Mihatovic I, Cordaro L, Windisch P, Friedmann A, Blanco Carrion J, et al. Comparison of a polyethylene glycol membrane and a collagen membrane for the treatment of bone dehiscence defects at bone level implants-A prospective, randomized, controlled, multicenter clinical trial. Clin Oral Implants Res. 2020;31:1105–15.

46. Cho Y-S, Hwang K-G, Jun SH, Tallarico M, Kwon AM, Park C-J. Radiologic comparative analysis between saline and platelet-rich fibrin filling after hydraulic transcrestal sinus lifting without adjunctive bone graft: A randomized controlled trial. Clin Oral Implants Res. 2020;31:1087–93.

47. Padhye NM, Mehta LK, Yadav N. Buccally displaced flap versus sub-epithelial connective tissue graft for peri-implant soft tissue augmentation: a pilot double-blind randomized controlled trial. Int J Implant Dent. 2020;6:48.

48. AAl MA, El Far M, Sheta NM, Fayyad A, El Desouky E, Nabi NA, et al. Correlation of Implant Stability Between Two Noninvasive Methods Using Submerged and Nonsubmerged Healing Protocols: A Randomized Clinical Trial. Journal of Oral Implantology. 2020;46:571–9.

49. Polymeri A, Anssari-Moin D, van der Horst J, Wismeijer D, Laine ML, Loos BG. Surgical treatment of peri-implantitis defects with two different xenograft granules: A randomized clinical pilot study. Clin Oral Implants Res. 2020;31:1047–60.

50. Shahdad S, Gamble E, Matani J, Zhang L, Gambôa A. Randomized clinical trial comparing PEG-based synthetic to porcine-derived collagen membrane in the preservation of alveolar bone following tooth extraction in anterior maxilla. Clin Oral Implants Res. 2020;31:1010–24.

51. Miguel MMV, Mathias-Santamaria IF, Rossato A, Ferraz LFF, Figueiredo-Neto AM, de Marco AC, et al. Microcurrent electrotherapy improves palatal wound healing: Randomized clinical trial. J Periodontol. 2021;92:244–53.

52. Koldsland OC, Aass AM. Supportive treatment following peri-implantitis surgery: An RCT using titanium curettes or chitosan brushes. J Clin Periodontol. 2020;47:1259–67.

53. Wang C-W, Ashnagar S, Gianfilippo RD, Arnett M, Kinney J, Wang H-L. Laser-assisted regenerative surgical therapy for peri-implantitis: A randomized controlled clinical trial. J Periodontol. 2021;92:378–88.

54. Wang J, Lerman G, Bittner N, Fan W, Lalla E, Papapanou PN. Immediate versus delayed temporization at posterior single implant sites: A randomized controlled trial. J Clin Periodontol. 2020;47:1281–91.

55. Abd-Elrahman A, Shaheen M, Askar N, Atef M. Socket shield technique vs conventional immediate implant placement with immediate temporization. Randomized clinical trial. Clin Implant Dent Relat Res. 2020;22:602–11.

56. Gulnahar Y, Kupeli I. Effect of Different Kinds of Music on Anxiety During Implant Surgery in Turkey: Randomized Controlled Study. Int J Oral Maxillofac Implants. 2020;35:762–6.

57. Bunk D, Eisenburger M, Häckl S, Eberhard J, Stiesch M, Grischke J. The effect of adjuvant oral irrigation on self-administered oral care in the management of peri-implant mucositis: A randomized controlled clinical trial. Clin Oral Implants Res. 2020;31:946–58.

58. Abou-Ayash S, Schimmel M, Kraus D, Mericske-Stern R, Albrecht D, Enkling N. Platform switching in two-implant bar-retained mandibular overdentures: 1-year results from a split-mouth randomized controlled clinical trial. Clin Oral Implants Res. 2020;31:968–79.

59. Arzouman A, Deas DE, Mills MP, Huynh-Ba G, Prihoda TJ, Mealey BL. Impact of different surgical protocols on dimensional changes of free soft tissue autografts: A randomized controlled trial. J Periodontol. 2021;92:45–53.

60. De Bruyckere T, Cabeza RG, Eghbali A, Younes F, Cleymaet R, Cosyn J. A randomized controlled study comparing guided bone regeneration with connective tissue graft to reestablish buccal convexity at implant sites: A 1-year volumetric analysis. Clin Implant Dent Relat Res. 2020;22:468–76.

61. Merli M, Bernardelli F, Giulianelli E, Carinci F, Mariotti G, Merli M, et al. Short-term comparison of two non-surgical treatment modalities of peri-implantitis: Clinical and microbiological outcomes in a two-factorial randomized controlled trial. J Clin Periodontol. 2020;47:1268–80.

62. Salles MM, de Cássia Oliveira V, Macedo AP, Silva-Lovato CH, de Freitas de Oliveira Paranhos H. Effectiveness of Brushing Associated With Oral Irrigation in Maintenance of Peri-Implant Tissues and Overdentures: Clinical Parameters and Patient Satisfaction. J Oral Implantol. 2021;47:117–23.

63. Kwon C, Lee JM, Suh JY, Seo SJ, Lee Y, Kim YG. Effects of an electric toothbrush combined with 3-color light-emitting diodes on antiplaque and bleeding control: a randomized controlled study. J Periodontal Implant Sci. 2020;50:251–9.

64. Rivara F, Macaluso GM, Toffoli A, Calciolari E, Goldoni M, Lumetti S. The effect of a 2-mm inter-implant distance on esthetic outcomes in immediately non-occlusally loaded platform shifted implants in healed ridges: 12-month results of a randomized clinical trial. Clin Implant Dent Relat Res. 2020;22:486–96.

65. Johnston W, Paterson M, Piela K, Davison E, Simpson A, Goulding M, et al. The systemic inflammatory response following hand instrumentation versus ultrasonic instrumentation-A randomized controlled trial. J Clin Periodontol. 2020;47:1087–97.

66. Clem D, Heard R, McGuire M, Scheyer ET, Richardson C, Toback G, et al. Comparison of Er,Cr:YSGG laser to minimally invasive surgical technique in the treatment of intrabony defects: Six-month results of a multicenter, randomized, controlled study. J Periodontol. 2021;92:496–506.

67. Whitt J, Al-Sabbagh M, Dawson D, Shehata E, Housley-Smith M, Tezanos A, et al. Efficacy of stem cell allograft in maxillary sinus bone regeneration: a randomized controlled clinical and blinded histomorphometric study. Int J Implant Dent. 2020;6:25.

68. Cockerham BL, Patel A, Greenwell H, Hill M, Shumway B, Hsu HT. Ridge Augmentation Comparing a Cancellous Block Allograft to an Osteoinductive Demineralized Bone Matrix Allograft: A Randomized, Controlled, Blinded Clinical Trial. Int J Periodontics Restorative Dent. 2020;40:571–8.

69. Al-Hezaimi K, Naghshbandi J, Alhuzaimi R, Alonizan F, AlQwizany I, Rotstein I. Regeneration of Secondary Dentin Using Recombinant Human Platelet-Derived Growth Factor and MTA for Pulp Capping: A Randomized Controlled Human Clinical Trial. Int J Periodontics Restorative Dent. 2020;40:477–85.

70. Sivolella S, Botticelli D, Prasad S, Ricci S, Bressan E, Prasad H. Evaluation and comparison of histologic changes and implant survival in extraction sites immediately grafted with two different xenografts: A randomized clinical pilot study. Clin Oral Implants Res. 2020;31:825–35.

71. Jiang X, Di P, Ren S, Zhang Y, Lin Y. Hard and soft tissue alterations during the healing stage of immediate implant placement and provisionalization with or without connective tissue graft: A randomized clinical trial. J Clin Periodontol. 2020;47:1006–15.

72. Zuhr O, Rebele SF, Vach K, Petsos H, Hürzeler MB, Research Group for Oral Soft Tissue Biology & Wound Healing. Tunnel technique with connective tissue graft versus coronally advanced flap with enamel matrix derivate for root coverage: 2-year results of an RCT using 3D digital measuring for volumetric comparison of gingival dimensions. J Clin Periodontol. 2020;47:1144–58.

73. Li Manni L, Lecloux G, Rompen E, Aouini W, Shapira L, Lambert F. Clinical and radiographic assessment of circular versus triangular cross-section neck Implants in the posterior maxilla: A 1-year randomized controlled trial. Clin Oral Implants Res. 2020;31:814–24.

74. Cheng G-L, Leblebicioglu B, Li J, Chien H-H. Soft tissue healing around platform-switching and platform-matching single implants: A randomized clinical trial. J Periodontol. 2020;91:1609–20.

75. Pamato S, Honório HM, da Costa JA, Traebert JL, Bonfante EA, Pereira JR. The influence of titanium base abutments on peri-implant soft tissue inflammatory parameters and marginal bone loss: A randomized clinical trial. Clin Implant Dent Relat Res. 2020;22:542–8.

76. Harrison PL, Stuhr S, Shaddox LM. The impact of a modified electronic probe tip design on patient perception of discomfort during periodontal probing using standardized probing force: A randomized controlled trial. J Clin Periodontol. 2020;47:933–40.

77. Jasser E, Salami Z, El Hage F, Makzoumé J, Boulos PJ. Masticatory Efficiency in Implant-Supported Fixed Complete Dentures Compared with Conventional Dentures: A Randomized Clinical Trial by Color-Mixing Analysis Test. Int J Oral Maxillofac Implants. 2020;35:599–606.

78. Imai H, Iezzi G, Piattelli A, Ferri M, Apaza Alccayhuaman KA, Botticelli D. Influence of the Dimensions of the Antrostomy on Osseointegration of Mini-implants Placed in the Grafted Region After Sinus Floor Elevation: A Randomized Clinical Trial. Int J Oral Maxillofac Implants. 2020;35:591–8.

79. Lee JY, Choi YY, Choi Y, Jin BH. Efficacy of non-surgical treatment accompanied by professional toothbrushing in the treatment of chronic periodontitis in patients with type 2 diabetes mellitus: a randomized controlled clinical trial. J Periodontal Implant Sci. 2020;50:83–96.

80. Tao D, Ling MR, Feng X-P, Gallob J, Souverain A, Yang W, et al. Efficacy of an anhydrous stannous fluoride toothpaste for relief of dentine hypersensitivity: A randomized clinical study. J Clin Periodontol. 2020;47:962–9.

81. Levine JB, Goncalves J, Nguyen D, Nguyen O, Hasturk H. Efficacy of a novel post-foaming dental gel on gingival inflammation: A randomized controlled clinical trial. J Periodontol. 2020;91:1569–83.

82. Abduo J, Lee CL, Sarfarazi G, Xue B, Judge R, Darby I. Encode Protocol Versus Conventional Protocol for Single-Implant Restoration: A Prospective 2-Year Follow-Up Randomized Controlled Trial. J Oral Implantol. 2021;47:36–43.

83. Alqahtani F, Alqhtani N, Celur SL, Divakar DD, Al-Kheraif AA, Alkhtani F. Efficacy of Nonsurgical Mechanical Debridement With and Without Adjunct Low-Level Laser Therapy in the Treatment of Peri-Implantitis: A Randomized Controlled Trial. J Oral Implantol. 2020;46:526–31.

84. Natto Z, Parashis A, Jeong YN. Soft Tissue changes after using Collagen Matrix Seal or Collagen Sponge with Allograft in Ridge Preservation:A Randomized Controlled Volumetric Study. J Oral Implantol. 2020. https://doi.org/10.1563/aaid-joi-D-19-00080.

85. Schuster AJ, Marcello-Machado RM, Bielemann AM, Possebon AP da R, Chagas Júnior OL, Faot F. Immediate vs conventional loading of Facility-Equator system in mandibular overdenture wearers: 1-year RCT with clinical, biological, and functional evaluation. Clin Implant Dent Relat Res. 2020;22:270–80.

86. Wang Y, Liu HN, Zhen Z, Pelekos G, Wu MZ, Chen Y, et al. A randomized controlled trial of the effects of non-surgical periodontal therapy on cardiac function assessed by echocardiography in type 2 diabetic patients. J Clin Periodontol. 2020;47:726–36.

87. Hartlev J, Erik Nørholt S, Spin-Neto R, Kraft D, Schou S, Isidor F. Histology of augmented autogenous bone covered by a platelet-rich fibrin membrane or deproteinized bovine bone mineral and a collagen membrane: A pilot randomized controlled trial. Clin Oral Implants Res. 2020;31:694–704.

88. Lobato RPB, Kinalski M de A, Martins TM, Agostini BA, Bergoli CD, Dos Santos MBF. Influence of low-level laser therapy on implant stability in implants placed in fresh extraction sockets: A randomized clinical trial. Clin Implant Dent Relat Res. 2020;22:261–9.

89. Maier J, Reiniger APP, Sfreddo CS, Wikesjö UM, Kantorski KZ, Moreira CHC. Effect of self-performed mechanical plaque control frequency on gingival health in subjects with a history of periodontitis: A Randomized Clinical Trial. J Clin Periodontol. 2020;47:834–41.

90. Philip J, Laine ML, Wismeijer D. Adjunctive effect of mouthrinse on treatment of peri-implant mucositis using mechanical debridement: A randomized clinical trial. J Clin Periodontol. 2020;47:883–91.

91. Stumbras A, Januzis G, Gervickas A, Kubilius R, Juodzbalys G. Randomized and Controlled Clinical Trial of Bone Healing After Alveolar Ridge Preservation Using Xenografts and Allografts Versus Plasma Rich in Growth Factors. J Oral Implantol. 2020;46:515–25.

92. Rojo E, Stroppa G, Sanz-Martin I, Gonzalez-Martín O, Nart J. Soft tissue stability around dental implants after soft tissue grafting from the lateral palate or the tuberosity area - A randomized controlled clinical study. J Clin Periodontol. 2020;47:892–9.

93. Vincent-Bugnas S, Charbit Y, Charbit M, Dard M, Pippenger B. Maxillary Sinus Floor Elevation Surgery With BioOss Mixed With Enamel Matrix Derivative: A Human Randomized Controlled Clinical and Histologic Study. J Oral Implantol. 2020;46:507–13.

94. Paolantonio M, Di Tullio M, Giraudi M, Romano L, Secondi L, Paolantonio G, et al. Periodontal regeneration by leukocyte and platelet-rich fibrin with autogenous bone graft versus enamel matrix derivative with autogenous bone graft in the treatment of periodontal intrabony defects: A randomized non-inferiority trial. J Periodontol. 2020;91:1595–608.

95. Liu Y, Wang J, Chen F, Feng Y, Xie C, Li D. A reduced healing protocol for sinus floor elevation in a staged approach with deproteinized bovine bone mineral alone: A randomized controlled clinical trial of a 5-month healing in comparison to the 8-month healing. Clin Implant Dent Relat Res. 2020;22:281–91.

96. Linkevicius T, Puisys A, Linkevicius R, Alkimavicius J, Gineviciute E, Linkeviciene L. The influence of submerged healing abutment or subcrestal implant placement on soft tissue thickness and crestal bone stability. A 2-year randomized clinical trial. Clin Implant Dent Relat Res. 2020;22:497–506.

97. Cortellini P, Stalpers G, Mollo A, Tonetti MS. Periodontal regeneration versus extraction and dental implant or prosthetic replacement of teeth severely compromised by attachment loss to the apex: A randomized controlled clinical trial reporting 10-year outcomes, survival analysis and mean cumulative cost of recurrence. J Clin Periodontol. 2020;47:768–76.

98. Ovcharenko N, Greenwell H, Katwal D, Patel A, Hill M, Shumway B, et al. A Comparison of the Effect of Barrier Membranes on Clinical and Histologic Hard and Soft Tissue Healing with Ridge Preservation. Int J Periodontics Restorative Dent. 2020;40:365–71.

99. Sriwil M, Fakher MAA, Hasan K, Kasem T, Shwaiki T, Wassouf G. Comparison of Free Gingival Graft and Gingival Unit Graft for Treatment of Gingival Recession: A Randomized Controlled Trial. Int J Periodontics Restorative Dent. 2020;40:e103–10.

100. Atef M, Tarek A, Shaheen M, Alarawi RM, Askar N. Horizontal ridge augmentation using native collagen membrane vs titanium mesh in atrophic maxillary ridges: Randomized clinical trial. Clin Implant Dent Relat Res. 2020;22:156–66.

101. Jasa EE, Gradoville JM, Christiansen MM, Samson KK, Reinhardt RA, Payne JB, et al. Effects of enamel matrix derivative on clinical and inflammatory outcomes in periodontal maintenance patients: Randomized controlled clinical trial. J Periodontol. 2020;91:1400–8.

102. Graziani F, Peric M, Marhl U, Petrini M, Bettini L, Tonetti M, et al. Local application of enamel matrix derivative prevents acute systemic inflammation after periodontal regenerative surgery: A randomized controlled clinical trial. J Clin Periodontol. 2020;47:747–55.

103. Gou H, Fan R, Chen X, Li L, Wang X, Xu Y, et al. Adjunctive effects of laser therapy on somatosensory function and vasomotor regulation of periodontal tissues in patients with periodontitis: A randomized controlled clinical trial. J Periodontol. 2020;91:1307–17.

104. Merli M, Merli M, Mariotti G, Pagliaro U, Moscatelli M, Nieri M. Immediate versus early non-occlusal loading of dental implants placed flapless in partially edentulous patients: A 10-year randomized clinical trial. J Clin Periodontol. 2020;47:621–9.

105. Imai H, Lang NP, Ferri M, Hirota A, Apaza Alccayhuaman KA, Botticelli D. Tomographic Assessment on the Influence of the Use of a Collagen Membrane on Dimensional Variations to Protect the Antrostomy After Maxillary Sinus Floor Augmentation: A Randomized Clinical Trial. Int J Oral Maxillofac Implants. 2020;35:350–6.

106. Simonffy L, Minya F, Trimmel B, Lacza Z, Dobo-Nagy C. Albumin-Impregnated Allograft Filling of Surgical Extraction Sockets Achieves Better Bone Remodeling Than Filling with Either Blood Clot or Bovine Xenograft. Int J Oral Maxillofac Implants. 2020;35:297–304.

107. Jacobs BP, Zadeh HH, De Kok I, Cooper L. A Randomized Controlled Trial Evaluating Grafting the Facial Gap at Immediately Placed Implants. Int J Periodontics Restorative Dent. 2020;40:383–92.

108. Thoma DS, Bienz SP, Lim H-C, Lee WZ, Hämmerle CHF, Jung RE. Explorative randomized controlled study comparing soft tissue thickness, contour changes, and soft tissue handling of two ridge preservation techniques and spontaneous healing two months after tooth extraction. Clin Oral Implants Res. 2020;31:565–74.

109. Byun S-H, Kim S-Y, Lee H, Lim H-K, Kim J-W, Lee U-L, et al. Soft tissue expander for vertically atrophied alveolar ridges: Prospective, multicenter, randomized controlled trial. Clin Oral Implants Res. 2020;31:585–94.

110. Keceli HG, Ercan N, Karsiyaka Hendek M, Kisa U, Mesut B, Olgun E. The effect of the systemic folic acid intake as an adjunct to scaling and root planing on clinical parameters and homocysteine and C-reactive protein levels in gingival crevicular fluid of periodontitis patients: A randomized placebo-controlled clinical trial. J Clin Periodontol. 2020;47:602–13.

111. Castro Dos Santos NC, Andere NMRB, Araujo CF, de Marco AC, Kantarci A, Van Dyke TE, et al. Omega-3 PUFA and aspirin as adjuncts to periodontal debridement in patients with periodontitis and type 2 diabetes mellitus: Randomized clinical trial. J Periodontol. 2020;91:1318–27.

112. Thoma DS, Gasser TJW, Jung RE, Hämmerle CHF. Randomized controlled clinical trial comparing implant sites augmented with a volume-stable collagen matrix or an autogenous connective tissue graft: 3-year data after insertion of reconstructions. J Clin Periodontol. 2020;47:630–9.

113. Oh S-L, Ji C, Azad S. Free gingival grafts for implants exhibiting a lack of keratinized mucosa: Extended follow-up of a randomized controlled trial. J Clin Periodontol. 2020;47:777–85.

114. Abdal-Wahab M, Abdel Ghaffar KA, Ezzatt OM, Hassan AAA, El Ansary MMS, Gamal AY. Regenerative potential of cultured gingival fibroblasts in treatment of periodontal intrabony defects (randomized clinical and biochemical trial). J Periodontal Res. 2020;55:441–52.

115. Galindo-Moreno P, Padial-Molina M, Lopez-Chaichio L, Gutiérrez-Garrido L, Martín-Morales N, O’Valle F. Algae-derived hydroxyapatite behavior as bone biomaterial in comparison with anorganic bovine bone: A split-mouth clinical, radiological, and histologic randomized study in humans. Clin Oral Implants Res. 2020;31:536–48.

116. Nelson AC, Mealey BL. A randomized controlled trial on the impact of healing time on wound healing following ridge preservation using a 70%/30% combination of mineralized and demineralized freeze-dried bone allograft. J Periodontol. 2020;91:1256–63.

117. Çankaya ZT, Ünsal B, Gürbüz S, Bakirarar B, Tamam E. Efficiency of Concentrated Growth Factor in the Surgical Treatment of Multiple Adjacent Papillary Losses: A Randomized, Controlled, Examiner-Blinded Clinical Trial Using CAD/CAM. Int J Periodontics Restorative Dent. 2020;40:e73–83.

118. Çankaya ZT, Gürbüz S, Bakirarar B, Kurtiş B. Evaluation of the Effect of Hyaluronic Acid Application on the Vascularization of Free Gingival Graft for Both Donor and Recipient Sites with Laser Doppler Flowmetry: A Randomized, Examiner-Blinded, Controlled Clinical Trial. Int J Periodontics Restorative Dent. 2020;40:233–43.

119. Parween S, George JP, Prabhuji M. Treatment of Multiple Mandibular Gingival Recession Defects Using MCAT Technique and SCTG With and Without rhPDGF-BB: A Randomized Controlled Clinical Trial. Int J Periodontics Restorative Dent. 2020;40:e43–51.

120. Merli M, Mariotti G, Pagliaro U, Mazzoni A, Moscatelli M, Nieri M. The Fence Technique: 100% Autogenous Bone Graft vs 50% Deproteinized Bovine Bone Matrix and 50% Autogenous Bone Graft. A Histologic Randomized Controlled Trial. Int J Periodontics Restorative Dent. 2020;40:181–90.

121. Afacan B, Çınarcık S, Gürkan A, Özdemir G, İlhan HA, Vural C, et al. Full-mouth disinfection effects on gingival fluid calprotectin, osteocalcin, and N-telopeptide of Type I collagen in severe periodontitis. J Periodontol. 2020;91:638–50.

122. Schlagenhauf U, Rehder J, Gelbrich G, Jockel-Schneider Y. Consumption of Lactobacillus reuteri-containing lozenges improves periodontal health in navy sailors at sea: A randomized controlled trial. J Periodontol. 2020;91:1328–38.

123. Wittneben J-G, Gavric J, Sailer I, Buser D, Wismeijer D. Clinical and esthetic outcomes of two different prosthetic workflows for implant-supported all-ceramic single crowns-3 year results of a randomized multicenter clinical trail. Clin Oral Implants Res. 2020;31:495–505.

124. De Bruyckere T, Cosyn J, Younes F, Hellyn J, Bekx J, Cleymaet R, et al. A randomized controlled study comparing guided bone regeneration with connective tissue graft to re-establish buccal convexity: One-year aesthetic and patient-reported outcomes. Clin Oral Implants Res. 2020;31:507–16.

125. Waller T, Herzog M, Thoma DS, Hüsler J, Hämmerle CHF, Jung RE. Long-term clinical and radiographic results after treatment or no treatment of small buccal bone dehiscences at posterior dental implants: A randomized, controlled clinical trial. Clin Oral Implants Res. 2020;31:517–25.

126. Sluka B, Naenni N, Jung RE, Attin T, Schmidlin PR, Sahrmann P. Changes of radiopacity around implants of different lengths: Five-year follow-up data of a randomized clinical trial. Clin Oral Implants Res. 2020;31:488–94.

127. Vohra F, Bukhari IA, Sheikh SA, Albaijan R, Naseem M, Hussain M. Effectiveness of scaling and root planing with and without adjunct probiotic therapy in the treatment of chronic periodontitis among shamma users and non-users: A randomized controlled trial. J Periodontol. 2020;91:1177–85.

128. Qian S-J, Mo J-J, Si M-S, Qiao S-C, Shi J-Y, Lai H-C. Long-term outcomes of osteotome sinus floor elevation with or without bone grafting: The 10-year results of a randomized controlled trial. J Clin Periodontol. 2020;47:1016–25.

129. Gao W, Tang H, Wang D, Zhou X, Song Y, Wang Z. Effect of short-term vitamin D supplementation after nonsurgical periodontal treatment: A randomized, double-masked, placebo-controlled clinical trial. J Periodontal Res. 2020;55:354–62.

130. Varga E, Antal M, Major L, Kiscsatári R, Braunitzer G, Piffkó J. Guidance means accuracy: A randomized clinical trial on freehand versus guided dental implantation. Clin Oral Implants Res. 2020;31:417–30.

131. Koller M, Steyer E, Theisen K, Stagnell S, Jakse N, Payer M. Two-piece zirconia versus titanium implants after 80 months: Clinical outcomes from a prospective randomized pilot trial. Clin Oral Implants Res. 2020;31:388–96.

132. Praça L de FG, Teixeira RC, Rego RO. Influence of abutment disconnection on peri-implant marginal bone loss: A randomized clinical trial. Clin Oral Implants Res. 2020;31:341–51.

133. Aslan S, Buduneli N, Cortellini P. Clinical outcomes of the entire papilla preservation technique with and without biomaterials in the treatment of isolated intrabony defects: A randomized controlled clinical trial. J Clin Periodontol. 2020;47:470–8.

134. Lasserre JF, Brecx MC, Toma S. Implantoplasty Versus Glycine Air Abrasion for the Surgical Treatment of Peri-implantitis: A Randomized Clinical Trial. Int J Oral Maxillofac Implants. 2020;35:197–206.

135. Sakuma S, Piattelli A, Baldi N, Ferri M, Iezzi G, Botticelli D. Bone Healing at Implants Placed in Sites Prepared Either with a Sonic Device or Drills: A Split-Mouth Histomorphometric Randomized Controlled Trial. Int J Oral Maxillofac Implants. 2020;35:187–95.

136. Mundt T, Schwahn C, Heinemann F, Schimmel M, Lucas C, Al Jaghsi A. Stabilizing Removable Partial Dentures by Immediate or Delayed Loading of Mini-implants: Chewing Efficiency in a Randomized Controlled Clinical Trial. Int J Oral Maxillofac Implants. 2020;35:178–86.

137. Ozsagir ZB, Saglam E, Sen Yilmaz B, Choukroun J, Tunali M. Injectable platelet-rich fibrin and microneedling for gingival augmentation in thin periodontal phenotype: A randomized controlled clinical trial. J Clin Periodontol. 2020;47:489–99.

138. Salvi GE, Moëne R, Wallkamm B, Hicklin SP, Bischof M, Nedir R, et al. Clinical and radiographic changes at tissue level implants with either a machined or a modified transmucosal neck surface: A 3-year multicentre randomized controlled proof-of-concept study. J Clin Periodontol. 2020;47:500–8.

139. Sapata VM, Llanos AH, Cesar Neto JB, Jung RE, Thoma DS, Hämmerle CHF, et al. Deproteinized bovine bone mineral is non-inferior to deproteinized bovine bone mineral with 10% collagen in maintaining the soft tissue contour post-extraction: A randomized trial. Clin Oral Implants Res. 2020;31:294–301.

140. de Siqueira RAC, Savaget Gonçalves Junior R, Dos Santos PGF, de Mattias Sartori IA, Wang H-L, Fontão FNGK. Effect of different implant placement depths on crestal bone levels and soft tissue behavior: A 5-year randomized clinical trial. Clin Oral Implants Res. 2020;31:282–93.

141. Ahmad N, Tewari S, Narula SC, Sharma RK, Tanwar N. Platelet-rich fibrin along with a modified minimally invasive surgical technique for the treatment of intrabony defects: a randomized clinical trial. J Periodontal Implant Sci. 2019;49:355–65.

142. Neves FL da S, Augusto Silveira C, Mathias-Santamaria IF, Miguel MMV, Ferraz LFF, Casarin RCV, et al. Randomized clinical trial evaluating single maxillary gingival recession treatment with connective tissue graft and tunnel or trapezoidal flap: 2-year follow-up. J Periodontol. 2019. https://doi.org/10.1002/JPER.19-0436.

143. Wang I-C, Chan H-L, Kinney J, Wang H-L. Volumetric facial contour changes of immediately placed implants with and without immediate provisionalization. J Periodontol. 2020;91:906–16.

144. Aydemir CA, Arısan V. Accuracy of dental implant placement via dynamic navigation or the freehand method: A split-mouth randomized controlled clinical trial. Clin Oral Implants Res. 2020;31:255–63.

145. Raj SC, Praharaj K, Barik AK, Patnaik K, Mahapatra A, Mohanty D, et al. Retraction With and Without Piezocision-Facilitated Orthodontics: A Randomized Controlled Trial. Int J Periodontics Restorative Dent. 2020;40:e19–26.

146. Bittner N, Planzos L, Volchonok A, Tarnow D, Schulze-Späte U. Evaluation of Horizontal and Vertical Buccal Ridge Dimensional Changes After Immediate Implant Placement and Immediate Temporization With and Without Bone Augmentation Procedures: Short-Term, 1-Year Results. A Randomized Controlled Clinical Trial. Int J Periodontics Restorative Dent. 2020;40:83–93.

147. Cairo F, Cortellini P, Nieri M, Pilloni A, Barbato L, Pagavino G, et al. Coronally advanced flap and composite restoration of the enamel with or without connective tissue graft for the treatment of single maxillary gingival recession with non-carious cervical lesion. A randomized controlled clinical trial. J Clin Periodontol. 2020;47:362–71.

148. Lee J-H, Kim D-H, Jeong S-N. Adjunctive use of enamel matrix derivatives to porcine-derived xenograft for the treatment of one-wall intrabony defects: Two-year longitudinal results of a randomized controlled clinical trial. J Periodontol. 2020;91:880–9.

149. Uraz A, Isler SC, Cula S, Tunc S, Yalim M, Cetiner D. Platform-switched implants vs platform-matched implants placed in different implant-abutment interface positions: A prospective randomized clinical and microbiological study. Clin Implant Dent Relat Res. 2020;22:59–68.

150. Amari Y, Piattelli A, Apaza Alccayhuaman KA, Mesa NF, Ferri M, Iezzi G, et al. Bone healing at non-submerged implants installed with different insertion torques: a split-mouth histomorphometric randomized controlled trial. Int J Implant Dent. 2019;5:39.

151. Mousquer CR, Della Bona A, Milani DC, Callegari-Jacques SM, Ishikawa K, Mayer MPA, et al. Are Lactobacillus salivarius G60 and inulin more efficacious to treat patients with oral halitosis and tongue coating than the probiotic alone and placebo? A randomized clinical trial. J Periodontol. 2020;91:775–83.

152. Perez A, Caiazzo A, Valente NA, Toti P, Alfonsi F, Barone A. Standard vs customized healing abutments with simultaneous bone grafting for tissue changes around immediate implants. 1-year outcomes from a randomized clinical trial. Clin Implant Dent Relat Res. 2020;22:42–53.

153. Gursoytrak B, Ataoglu H. Use of resonance frequency analysis to evaluate the effects of surface properties on the stability of different implants. Clin Oral Implants Res. 2020;31:239–45.

154. Lim H-C, Seo S, Thoma DS, Park J-C, Hong J-Y, Shin S-Y. Late implant placement following ridge preservation versus early implant placement: A pilot randomized clinical trial for periodontally compromised non-molar extraction sites. J Clin Periodontol. 2020;47:247–56.

155. Khaled H, Atef M, Hakam M. Maxillary sinus floor elevation using hydroxyapatite nano particles vs tenting technique with simultaneous implant placement: A randomized clinical trial. Clin Implant Dent Relat Res. 2019;21:1241–52.

156. El Zahwy M, Taha SAAK, Mounir R, Mounir M. Assessment of vertical ridge augmentation and marginal bone loss using autogenous onlay vs inlay grafting techniques with simultaneous implant placement in the anterior maxillary esthetic zone: A randomized clinical trial. Clin Implant Dent Relat Res. 2019;21:1140–7.

157. Koo T-H, Song YW, Cha J-K, Jung U-W, Kim C-S, Lee J-S. Histologic analysis following grafting of damaged extraction sockets using deproteinized bovine or porcine bone mineral: A randomized clinical trial. Clin Oral Implants Res. 2020;31:93–102.

158. de Campos CG, Francischone CE, Souza Picorelli Assis NM, Devito KL, Sotto-Maior BS. Neurosensory Function and Implant Survival Rate Following Implant Placement With or Without an Interposed Bone Graft Between the Implant and Nerve: Prospective Clinical Trial. Int J Oral Maxillofac Implants. 2019;33:1450–6.

159. Laleman I, Pauwels M, Quirynen M, Teughels W. The usage of a lactobacilli probiotic in the non-surgical therapy of peri-implantitis: A randomized pilot study. Clin Oral Implants Res. 2020;31:84–92.

160. Sun C, Zhao J, Liu Z, Tan L, Huang Y, Zhao L, et al. Comparing conventional flap-less immediate implantation and socket-shield technique for esthetic and clinical outcomes: A randomized clinical study. Clin Oral Implants Res. 2020;31:181–91.

161. Areewong K, Chantaramungkorn M, Khongkhunthian P. Platelet-rich fibrin to preserve alveolar bone sockets following tooth extraction: A randomized controlled trial. Clin Implant Dent Relat Res. 2019;21:1156–63.

162. Camacho-Alonso F, Vilaplana-Vivo J, Caballero-Guerrero PM, Pato-Mourelo J, Sánchez-Siles M. Impact of audiovisual information on anxiety and fear in patients undergoing dental implant treatment. Clin Implant Dent Relat Res. 2019;21:1189–98.

163. Magrin GL, Rafael SNF, Passoni BB, Magini RS, Benfatti CAM, Gruber R, et al. Clinical and tomographic comparison of dental implants placed by guided virtual surgery versus conventional technique: A split-mouth randomized clinical trial. J Clin Periodontol. 2020;47:120–8.

164. Gandhi KK, Pavaskar R, Cappetta EG, Drew HJ. Effectiveness of Adjunctive Use of Low-Level Laser Therapy and Photodynamic Therapy After Scaling and Root Planing in Patients with Chronic Periodontitis. Int J Periodontics Restorative Dent. 2019;39:837–43.

165. Cagidiaco EF, Discepoli N, Goracci C, Carboncini F, Vigolo P, Ferrari M. Randomized Clinical Trial on Single Zirconia Crowns with Feather-Edge vs Chamfer Finish Lines: Four-Year Results. Int J Periodontics Restorative Dent. 2019;39:817–26.

166. Schnabl D, Goebel G, Kadletz A, Gaenzer H, Steiner R, Laimer J, et al. Cleansing efficacy of waist-shaped inter-dental brushes. A randomized-controlled crossover study. J Clin Periodontol. 2020;47:30–5.

167. Guida L, Annunziata M, Esposito U, Sirignano M, Torrisi P, Cecchinato D. 6-mm-short and 11-mm-long implants compared in the full-arch rehabilitation of the edentulous mandible: A 3-year multicenter randomized controlled trial. Clin Oral Implants Res. 2020;31:64–73.

168. Preus HR, Al-Lami Q, Baelum V. Oral hygiene revisited. The clinical effect of a prolonged oral hygiene phase prior to periodontal therapy in periodontitis patients. A randomized clinical study. J Clin Periodontol. 2020;47:36–42.

169. Temmerman A, Cortellini S, Van Dessel J, De Greef A, Jacobs R, Dhondt R, et al. Bovine-derived xenograft in combination with autogenous bone chips versus xenograft alone for the augmentation of bony dehiscences around oral implants: A randomized, controlled, split-mouth clinical trial. J Clin Periodontol. 2020;47:110–9.

170. Soriano-Lerma A, Magán-Fernández A, Gijón J, Sánchez-Fernández E, Soriano M, García-Salcedo JA, et al. Short-term effects of hyaluronic acid on the subgingival microbiome in peri-implantitis: A randomized controlled clinical trial. J Periodontol. 2020;91:734–45.

171. Ucan Yarkac F, Ogrum A, Gokturk O. Effects of non-surgical periodontal therapy on inflammatory markers of psoriasis: A randomized controlled trial. J Clin Periodontol. 2020;47:193–201.

172. Trinh HA, Dam VV, Le B, Pittayapat P, Thunyakitpisal P. Indirect Sinus Augmentation With and Without the Addition of a Biomaterial: A Randomized Controlled Clinical Trial. Implant Dent. 2019;28:571–7.

173. Wortmann DE, Boven CG, Schortinghuis J, Vissink A, Raghoebar GM. Patients’ appreciation of pre-implant augmentation of the severely resorbed maxilla with calvarial or anterior iliac crest bone:a randomized controlled trial. Int J Implant Dent. 2019;5:36.

174. Mercado F, Hamlet S, Ivanovski S. Subepithelial connective tissue graft with or without enamel matrix derivative for the treatment of multiple Class III-IV recessions in lower anterior teeth: A 3-year randomized clinical trial. J Periodontol. 2020;91:473–83.

175. Stewart B, Shibli JA, Araujo M, Figueiredo LC, Panagakos F, Matarazzo F, et al. Effects of a toothpaste containing 0.3% triclosan on periodontal parameters of subjects enrolled in a regular maintenance program: A secondary analysis of a 2-year randomized clinical trial. J Periodontol. 2020;91:596–605.

176. Bertl K, Neuner H, Meran A, Bertl MH, Reich I, Nemec M, et al. Does the time-point of orthodontic space closure initiation after tooth extraction affect the incidence of gingival cleft development? A randomized controlled clinical trial. J Periodontol. 2020;91:572–81.

177. Beretta M, Maiorana C, Cortinovis I, Poli PP. Tapered Screw Implants With Different Machined Neck Designs: A 3-Year Split-Mouth Randomized Controlled Prospective Study. J Oral Implantol. 2019;45:416–20.

178. Nedir R, Nurdin N, Huynh-Ba G, Bischof M. Change in Crown-to-Implant Ratio of Implants Placed in Grafted and Nongrafted Posterior Maxillary Sites: A 5-year Prospective Randomized Study. Int J Oral Maxillofac Implants. 2019;34:1231–6.

179. Laleman I, Pauwels M, Quirynen M, Teughels W. A dual-strain Lactobacilli reuteri probiotic improves the treatment of residual pockets: A randomized controlled clinical trial. J Clin Periodontol. 2020;47:43–53.

180. Eshkol-Yogev I, Tandlich M, Shapira L. Effect of implant neck design on primary and secondary implant stability in the posterior maxilla: A prospective randomized controlled study. Clin Oral Implants Res. 2019;30:1220–8.

181. Ucak Turer O, Ozcan M, Alkaya B, Surmeli S, Seydaoglu G, Haytac MC. Clinical evaluation of injectable platelet-rich fibrin with connective tissue graft for the treatment of deep gingival recession defects: A controlled randomized clinical trial. J Clin Periodontol. 2020;47:72–80.

182. Pelekos G, Ho SN, Acharya A, Leung WK, McGrath C. A double-blind, paralleled-arm, placebo-controlled and randomized clinical trial of the effectiveness of probiotics as an adjunct in periodontal care. J Clin Periodontol. 2019;46:1217–27.

183. Lee J-H, Kim D-H, Jeong S-N. Comparative assessment of anterior maxillary alveolar ridge preservation with and without adjunctive use of enamel matrix derivative: A randomized clinical trial. Clin Oral Implants Res. 2020;31:1–9.

184. Grassi FR, Grassi R, Rapone B, Alemanno G, Balena A, Kalemaj Z. Dimensional changes of buccal bone plate in immediate implants inserted through open flap, open flap and bone grafting and flapless techniques: A cone-beam computed tomography randomized controlled clinical trial. Clin Oral Implants Res. 2019;30:1155–64.

185. Lowy J, Kwon HS, Patel A, Greenwell H, Hill M, Katwal D, et al. The Effect of Platform-Switching Plus Laser Grooving on Peri-implant Hard and Soft Tissue Level: A Randomized, Controlled, Blinded Clinical Trial. Int J Periodontics Restorative Dent. 2019;39:669–74.

186. Shah KK, Kolte RA. Evaluation of Demineralized Freeze-Dried Bone Allograft in Combination with Chorion Membrane in the Treatment of Grade II Furcation Defects: A Randomized Controlled Trial. Int J Periodontics Restorative Dent. 2019;39:659–67.

187. Alkanan A, Greenwell H, Patel A, Hill M, Shumway B, Lowy J. Ridge Preservation Comparing the Clinical and Histologic Healing of Membrane vs No-Membrane Approach to Buccal Overlay Grafting. Int J Periodontics Restorative Dent. 2019;39:643–50.

188. Barootchi S, Tavelli L, Di Gianfilippo R, Byun H-Y, Oh T-J, Barbato L, et al. Long term assessment of root coverage stability using connective tissue graft with or without an epithelial collar for gingival recession treatment. A 12-year follow-up from a randomized clinical trial. J Clin Periodontol. 2019;46:1124–33.

189. Lim H-C, Shin H-S, Cho I-W, Koo K-T, Park J-C. Ridge preservation in molar extraction sites with an open-healing approach: A randomized controlled clinical trial. J Clin Periodontol. 2019;46:1144–54.

190. Ioannidis A, Heierle L, Hämmerle CHF, Hüsler J, Jung RE, Thoma DS. Prospective randomized controlled clinical study comparing two types of two-piece dental implants supporting fixed reconstructions-Results at 5 years of loading. Clin Oral Implants Res. 2019;30:1126–33.

191. Solakoglu Ö, Götz W, Heydecke G, Schwarzenbach H. Histological and immunohistochemical comparison of two different allogeneic bone grafting materials for alveolar ridge reconstruction: A prospective randomized trial in humans. Clin Implant Dent Relat Res. 2019;21:1002–16.

192. Hirota A, Lang NP, Ferri M, Fortich Mesa N, Apaza Alccayhuaman KA, Botticelli D. Tomographic evaluation of the influence of the placement of a collagen membrane subjacent to the sinus mucosa during maxillary sinus floor augmentation: a randomized clinical trial. Int J Implant Dent. 2019;5:31.

193. G F Tresguerres F, Cortes ARG, Hernandez Vallejo G, Cabrejos-Azama J, Tamimi F, Torres J. Clinical and radiographic outcomes of allogeneic block grafts for maxillary lateral ridge augmentation: A randomized clinical trial. Clin Implant Dent Relat Res. 2019;21:1087–98.

194. Tinto M, Sartori M, Pizzi I, Verga A, Longoni S. Melatonin as host modulating agent supporting nonsurgical periodontal therapy in patients affected by untreated severe periodontitis: A preliminary randomized, triple-blind, placebo-controlled study. J Periodontal Res. 2020;55:61–7.

195. Kutkut A, Rezk M, Zephyr D, Dawson D, Frazer R, Al-Sabbagh M. Immediate Loading of Unsplinted Implant Retained Mandibular Overdenture: A Randomized Controlled Clinical Study. J Oral Implantol. 2019;45:378–89.

196. Park J-H, Shin S-W, Lee J-Y. Bar versus ball attachments for maxillary four-implant retained overdentures: A randomized controlled trial. Clin Oral Implants Res. 2019;30:1076–84.

197. Lai VJ, Michalek JE, Liu Q, Mealey BL. Ridge preservation following tooth extraction using bovine xenograft compared with porcine xenograft: A randomized controlled clinical trial. J Periodontol. 2020;91:361–8.

198. Angst PDM, Finger Stadler A, Mendez M, Oppermann RV, van der Velden U, Gomes SC. Supportive periodontal therapy in moderate-to-severe periodontitis patients: A two-year randomized clinical trial. J Clin Periodontol. 2019;46:1083–93.

199. Lu H, Zhao Y, Feng X, He L, Meng H. Microbiome in maintained periodontitis and its shift over a single maintenance interval of 3 months. J Clin Periodontol. 2019;46:1094–104.

200. Jentsch HFR, Heusinger T, Weickert A, Eick S. Professional tooth cleaning prior to non-surgical periodontal therapy: A randomized clinical trial. J Periodontol. 2020;91:174–82.

201. Zhang Y, Tian J, Wei D, Di P, Lin Y. Quantitative clinical adjustment analysis of posterior single implant crown in a chairside digital workflow: A randomized controlled trial. Clin Oral Implants Res. 2019;30:1059–66.

202. Ahmedbeyli C, Dirikan Ipçi S, Cakar G, Yılmaz S, Chambrone L. Coronally advanced flap and envelope type of flap plus acellular dermal matrix graft for the treatment of thin phenotype multiple recession defects. A randomized clinical trial. J Clin Periodontol. 2019;46:1024–9.

203. Benic GI, Eisner BM, Jung RE, Basler T, Schneider D, Hämmerle CHF. Hard tissue changes after guided bone regeneration of peri-implant defects comparing block versus particulate bone substitutes: 6-month results of a randomized controlled clinical trial. Clin Oral Implants Res. 2019;30:1016–26.

204. Ko K-A, Kim S, Choi S-H, Lee J-S. Randomized controlled clinical trial on calcium phosphate coated and conventional SLA surface implants: 1-year study on survival rate and marginal bone level. Clin Implant Dent Relat Res. 2019;21:995–1001.

205. Husejnagic S, Lettner S, Laky M, Georgopoulos A, Moritz A, Rausch-Fan X. Photoactivated disinfection in periodontal treatment: A randomized controlled clinical split-mouth trial. J Periodontol. 2019;90:1260–9.

206. de Tapia B, Mozas C, Valles C, Nart J, Sanz M, Herrera D. Adjunctive effect of modifying the implant-supported prosthesis in the treatment of peri-implant mucositis. J Clin Periodontol. 2019;46:1050–60.

207. Rotundo R, Genzano L, Patel D, D’Aiuto F, Nieri M. Adjunctive benefit of a xenogenic collagen matrix associated with coronally advanced flap for the treatment of multiple gingival recessions: A superiority, assessor-blind, randomized clinical trial. J Clin Periodontol. 2019;46:1013–23.

208. Chan H-L, George F, Wang I-C, Suárez López Del Amo F, Kinney J, Wang H-L. A randomized controlled trial to compare aesthetic outcomes of immediately placed implants with and without immediate provisionalization. J Clin Periodontol. 2019;46:1061–9.

209. Nakano M, Yoshida A, Wakabayashi H, Tanaka M, Yamauchi K, Abe F, et al. Effect of tablets containing lactoferrin and lactoperoxidase on gingival health in adults: A randomized, double-blind, placebo-controlled clinical trial. J Periodontal Res. 2019;54:702–8.

210. Yashima A, Morozumi T, Yoshie H, Hokari T, Izumi Y, Akizuki T, et al. Biological responses following one-stage full-mouth scaling and root planing with and without azithromycin: Multicenter randomized trial. J Periodontal Res. 2019;54:709–19.

211. Reis R, Nicolau P, Calha N, Messias A, Guerra F. Immediate versus early loading protocols of titanium-zirconium narrow-diameter implants for mandibular overdentures in edentulous patients: 1-year results from a randomized controlled trial. Clin Oral Implants Res. 2019;30:953–61.

212. Araujo CF, Andere NMRB, Castro Dos Santos NC, Mathias-Santamaria IF, Reis AA, de Oliveira LD, et al. Two different antibiotic protocols as adjuncts to one-stage full-mouth ultrasonic debridement to treat generalized aggressive periodontitis: A pilot randomized controlled clinical trial. J Periodontol. 2019;90:1431–40.

213. Patel A, Greenwell H, Hill M, Shumway B, Radmall A. Ridge Augmentation Comparing an Allograft Plus Autogenous Bone Chips to an Osteoinductive Demineralized Bone Matrix: A Clinical and Histologic Study in Humans. Implant Dent. 2019;28:613–20.

214. Tavelli L, Barootchi S, Di Gianfilippo R, Modarressi M, Cairo F, Rasperini G, et al. Acellular dermal matrix and coronally advanced flap or tunnel technique in the treatment of multiple adjacent gingival recessions. A 12-year follow-up from a randomized clinical trial. J Clin Periodontol. 2019;46:937–48.

215. Smitkarn P, Subbalekha K, Mattheos N, Pimkhaokham A. The accuracy of single-tooth implants placed using fully digital-guided surgery and freehand implant surgery. J Clin Periodontol. 2019;46:949–57.

216. Farina R, Franceschetti G, Travaglini D, Consolo U, Minenna L, Schincaglia GP, et al. Radiographic outcomes of transcrestal and lateral sinus floor elevation: One-year results of a bi-center, parallel-arm randomized trial. Clin Oral Implants Res. 2019;30:910–9.

217. Cucchi A, Sartori M, Aldini NN, Vignudelli E, Corinaldesi G. A Proposal of Pseudo-periosteum Classification After GBR by Means of Titanium-Reinforced d-PTFE Membranes or Titanium Meshes Plus Cross-Linked Collagen Membranes. Int J Periodontics Restorative Dent. 2019;39:e157–65.

218. Guarnieri R, Di Nardo D, Di Giorgio G, Miccoli G, Testarelli L. Influence of the Microgap/Interface Vertical Position on Early Marginal Bone Remodeling Around One-Stage Implants with Laser-Microtextured Collar Surface: A Randomized Clinical Study. Int J Periodontics Restorative Dent. 2019;39:553–60.

219. Schneider D, Sancho-Puchades M, Mir-Marí J, Mühlemann S, Jung R, Hämmerle C. A Randomized Controlled Clinical Trial Comparing Conventional and Computer-Assisted Implant Planning and Placement in Partially Edentulous Patients. Part 4: Accuracy of Implant Placement. Int J Periodontics Restorative Dent. 2019;39:e111–22.

220. Sancho-Puchades M, Alfaro FH, Naenni N, Jung R, Hämmerle C, Schneider D. A Randomized Controlled Clinical Trial Comparing Conventional And Computer-Assisted Implant Planning and Placement in Partially Edentulous Patients. Part 2: Patient Related Outcome Measures. Int J Periodontics Restorative Dent. 2019;39:e99–110.

221. Papapetros D, Karagiannis V, Konstantinidis A, Apatzidou DA. Interim tissue changes following connective tissue grafting and two-stage implant placement. A randomized clinical trial. J Clin Periodontol. 2019;46:958–68.

222. Ouyyamwongs W, Leepong N, Suttapreyasri S. Alveolar Ridge Preservation Using Autologous Demineralized Tooth Matrix and Platelet-Rich Fibrin Versus Platelet-Rich Fibrin Alone: A Split-Mouth Randomized Controlled Clinical Trial. Implant Dent. 2019;28:455–62.

223. ElSyad MA, Alameldeen HE, Elsaih EA. Four-implant-supported fixed prosthesis and milled bar overdentures for rehabilitation of the edentulous mandible: A 1-year randomized controlled clinical and radiographic study. Int J Oral Maxillofac Implants. 2019;34:1493–503.

224. Koutouzis T, Adeinat B, Ali A. The influence of abutment macro-design on clinical and radiographic peri-implant tissue changes for guided, placed, and restored implants: A 1-year randomized controlled trial. Clin Oral Implants Res. 2019;30:882–91.

225. Furze D, Byrne A, Alam S, Brägger U, Wismeijer D, Wittneben J-G. Influence of the fixed implant-supported provisional phase on the esthetic final outcome of implant-supported crowns: 3-year results of a randomized controlled clinical trial. Clin Implant Dent Relat Res. 2019;21:649–55.

226. Thoma DS, Bienz SP, Payer M, Hüsler J, Schmidlin PR, Hämmerle CHF, et al. Randomized clinical study using xenograft blocks loaded with bone morphogenetic protein-2 or autogenous bone blocks for ridge augmentation - A three-dimensional analysis. Clin Oral Implants Res. 2019;30:872–81.

227. Salman A, Thacker S, Rubin S, Dhingra A, Ioannidou E, Schincaglia GP. Immediate versus delayed loading of mandibular implant-retained overdentures: A 60-month follow-up of a randomized clinical trial. J Clin Periodontol. 2019;46:863–71.

228. Shi J-Y, Li Y, Qiao S-C, Gu Y-X, Xiong Y-Y, Lai H-C. Short versus longer implants with osteotome sinus floor elevation for moderately atrophic posterior maxillae: A 1-year randomized clinical trial. J Clin Periodontol. 2019;46:855–62.

229. Huynh-Ba G, Hoders AB, Meister DJ, Prihoda TJ, Mills MP, Mealey BL, et al. Esthetic, clinical, and radiographic outcomes of two surgical approaches for single implant in the esthetic area: 1-year results of a randomized controlled trial with parallel design. Clin Oral Implants Res. 2019;30:745–59.

230. Carvalho Dutra B, Oliveira AMSD, Oliveira PAD, Miranda Cota LO, Silveira JO, Costa FO. Effects of topical application of 1% sodium alendronate gel in the surgical treatment of periodontal intrabony defects: A 6-month randomized controlled clinical trial. J Periodontol. 2019;90:1079–87.

231. Zhou X, Lin M, Zhang D, Song Y, Wang Z. Efficacy of Er:YAG laser on periodontitis as an adjunctive non-surgical treatment: A split-mouth randomized controlled study. J Clin Periodontol. 2019;46:539–47.

232. Vogl S, Stopper M, Hof M, Theisen K, Wegscheider WA, Lorenzoni M. Immediate occlusal vs nonocclusal loading of implants: A randomized prospective clinical pilot study and patient centered outcome after 36 months. Clin Implant Dent Relat Res. 2019;21:766–74.

233. Kaewsiri D, Panmekiate S, Subbalekha K, Mattheos N, Pimkhaokham A. The accuracy of static vs. dynamic computer-assisted implant surgery in single tooth space: A randomized controlled trial. Clin Oral Implants Res. 2019;30:505–14.

234. Clementini M, Agostinelli A, Castelluzzo W, Cugnata F, Vignoletti F, De Sanctis M. The effect of immediate implant placement on alveolar ridge preservation compared to spontaneous healing after tooth extraction: Radiographic results of a randomized controlled clinical trial. J Clin Periodontol. 2019;46:776–86.

235. Liu H, Liu R, Wang M, Yang J. Immediate implant placement combined with maxillary sinus floor elevation utilizing the transalveolar approach and nonsubmerged healing for failing teeth in the maxillary molar area: A randomized controlled trial clinical study with one-year follow-up. Clin Implant Dent Relat Res. 2019;21:462–72.

236. Spinato S, Stacchi C, Lombardi T, Bernardello F, Messina M, Zaffe D. Biological width establishment around dental implants is influenced by abutment height irrespective of vertical mucosal thickness: A cluster randomized controlled trial. Clin Oral Implants Res. 2019;30:649–59.

237. Thompson DM, Lee H-M, Stoner JA, Golub LM, Nummikoski PV, Payne JB. Loss of alveolar bone density in postmenopausal, osteopenic women is associated with circulating levels of gelatinases. J Periodontal Res. 2019;54:525–32.

238. Messias A, Rocha S, Wagner W, Wiltfang J, Moergel M, Behrens E, et al. Peri-implant marginal bone loss reduction with platform-switching components: 5-Year post-loading results of an equivalence randomized clinical trial. J Clin Periodontol. 2019;46:678–87.

239. Pico A, Martín-Lancharro P, Caneiro L, Nóvoa L, Batalla P, Blanco J. Influence of abutment height and implant depth position on interproximal peri-implant bone in sites with thin mucosa: A 1-year randomized clinical trial. Clin Oral Implants Res. 2019;30:595–602.

240. Moisa DH, Connolly JA, Cheng B, Lalla E. Impact of connective tissue graft thickness on surgical outcomes: A pilot randomized clinical trial. J Periodontol. 2019;90:966–72.

241. Asimakopoulou K, Nolan M, McCarthy C, Newton JT. The effect of risk communication on periodontal treatment outcomes: A randomized controlled trial. J Periodontol. 2019;90:948–56.

242. AlZarea BK. Randomized controlled clinical investigation on the association between personality profiles and the impacts of two types of maxillary anterior implant-supported crown restorations on daily living and dental satisfaction. Clin Implant Dent Relat Res. 2019;21:602–12.

243. Cha J-K, Song YW, Park S-H, Jung RE, Jung U-W, Thoma DS. Alveolar ridge preservation in the posterior maxilla reduces vertical dimensional change: A randomized controlled clinical trial. Clin Oral Implants Res. 2019;30:515–23.

244. Petsos H, Ratka-Krüger P, Neukranz E, Raetzke P, Eickholz P, Nickles K. Infrabony defects 20 years after open flap debridement and guided tissue regeneration. J Clin Periodontol. 2019;46:552–63.

245. Woelber JP, Gärtner M, Breuninger L, Anderson A, König D, Hellwig E, et al. The influence of an anti-inflammatory diet on gingivitis. A randomized controlled trial. J Clin Periodontol. 2019;46:481–90.

246. Ghazal SS, Huynh-Ba G, Aghaloo T, Dibart S, Froum S, O’Neal R, et al. A Randomized, Controlled, Multicenter Clinical Study Evaluating The Crestal Bone Level Change Of SLActive Bone Level Ø 3.3 mm Implants Compared To SLActive Bone Level Ø 4.1 mm Implants For Single-Tooth Replacement. Int J Oral Maxillofac Implants. 2019;34:708–18.

247. Aimetti M, Mariani GM, Ferrarotti F, Ercoli E, Liu CC, Romano F. Adjunctive efficacy of diode laser in the treatment of peri-implant mucositis with mechanical therapy: A randomized clinical trial. Clin Oral Implants Res. 2019;30:429–38.

248. Urban IA, Wessing B, Alández N, Meloni S, González-Martin O, Polizzi G, et al. A multicenter randomized controlled trial using a novel collagen membrane for guided bone regeneration at dehisced single implant sites: Outcome at prosthetic delivery and at 1-year follow-up. Clin Oral Implants Res. 2019;30:487–97.

249. Flichy-Fernández AJ, Blaya-Tárraga JA, O’Valle F, Padial-Molina M, Peñarrocha-Diago M, Galindo-Moreno P. Sinus floor elevation using particulate PLGA-coated biphasic calcium phosphate bone graft substitutes: A prospective histological and radiological study. Clin Implant Dent Relat Res. 2019;21:895–902.

250. Mounir M, Shalash M, Mounir S, Nassar Y, El Khatib O. Assessment of three dimensional bone augmentation of severely atrophied maxillary alveolar ridges using prebent titanium mesh vs customized poly-ether-ether-ketone (PEEK) mesh: A randomized clinical trial. Clin Implant Dent Relat Res. 2019;21:960–7.

251. Gil MS, Ishikawa-Nagai S, Elani HW, Da Silva JD, Kim DM, Tarnow D, et al. Comparison of the Color Appearance of Peri-implant Soft Tissue with Natural Gingiva Using Anodized Pink-Neck Implants and Pink Abutments: A Prospective Clinical Trial. Int J Oral Maxillofac Implants. 2019;34:752–8.

252. Mendoza-Azpur G, de la Fuente A, Chavez E, Valdivia E, Khouly I. Horizontal ridge augmentation with guided bone regeneration using particulate xenogenic bone substitutes with or without autogenous block grafts: A randomized controlled trial. Clin Implant Dent Relat Res. 2019;21:521–30.

253. Jo D-W, Cho Y-D, Seol Y-J, Lee Y-M, Lee H-J, Kim Y-K. A randomized controlled clinical trial evaluating efficacy and adverse events of different types of recombinant human bone morphogenetic protein-2 delivery systems for alveolar ridge preservation. Clin Oral Implants Res. 2019;30:396–409.

254. de Santana RB, de Mello Fonseca E, Furtado MB, de Santana CMM, Dibart S. Single-stage advanced versus rotated flaps in the treatment of gingival recessions: A 5-year longitudinal randomized clinical trial. J Periodontol. 2019;90:941–7.

255. Pelekos G, Lu JZ, Ho DKL, Graziani F, Cairo F, Cortellini P, et al. Aesthetic assessment after root coverage of multiple adjacent recessions with coronally advanced flap with adjunctive collagen matrix or connective tissue graft: Randomized clinical trial. J Clin Periodontol. 2019;46:564–71.

256. Ferraz BFR, Stuani VT, Passanezi E, Damante CA, Greghi SLA, de Rezende MLR, et al. Osteogenic cells transfer improving root coverage: A randomized clinical trial. J Periodontal Res. 2019;54:506–12.

257. Kraus RD, Epprecht A, Hämmerle CHF, Sailer I, Thoma DS. Cemented vs screw-retained zirconia-based single implant reconstructions: A 3-year prospective randomized controlled clinical trial. Clin Implant Dent Relat Res. 2019;21:578–85.

258. Hall J, Neilands J, Davies JR, Ekestubbe A, Friberg B. A randomized, controlled, clinical study on a new titanium oxide abutment surface for improved healing and soft tissue health. Clin Implant Dent Relat Res. 2019;21 Suppl 1:55–68.

259. Li Y, Qiao S-C, Gu Y-X, Zhang X-M, Shi J-Y, Lai H-C. A novel semiautomatic segmentation protocol to evaluate guided bone regeneration outcomes: A pilot randomized, controlled clinical trial. Clin Oral Implants Res. 2019;30:344–52.

260. Kashani H, Hilon J, Rasoul MH, Friberg B. Influence of a single preoperative dose of antibiotics on the early implant failure rate. A randomized clinical trial. Clin Implant Dent Relat Res. 2019;21:278–83.

261. de Tapia B, Valles C, Ribeiro-Amaral T, Mor C, Herrera D, Sanz M, et al. The adjunctive effect of a titanium brush in implant surface decontamination at peri-implantitis surgical regenerative interventions: A randomized controlled clinical trial. J Clin Periodontol. 2019;46:586–96.

262. Randall EF, Abou-Arraj RV, Geurs N, Griffin R, Reddy M, Geisinger M. The Effect of Dental Implant Collar Design on Crestal Bone Loss at 1 Year After Implant Placement. Int J Periodontics Restorative Dent. 2019;39:165–73.

263. Salem D, Alshihri A, Arguello E, Jung RE, Mohmed HA, Friedland B. Volumetric Analysis of Allogenic and Xenogenic Bone Substitutes Used in Maxillary Sinus Augmentations Utilizing Cone Beam CT: A Prospective Randomized Pilot Study. Int J Oral Maxillofac Implants. 2019;34:920–6.

264. Pulcini A, Bollaín J, Sanz-Sánchez I, Figuero E, Alonso B, Sanz M, et al. Clinical effects of the adjunctive use of a 0.03% chlorhexidine and 0.05% cetylpyridinium chloride mouth rinse in the management of peri-implant diseases: A randomized clinical trial. J Clin Periodontol. 2019;46:342–53.

265. Montenegro MM, Ribeiro IWJ, Kampits C, Saffi MAL, Furtado MV, Polanczyk CA, et al. Randomized controlled trial of the effect of periodontal treatment on cardiovascular risk biomarkers in patients with stable coronary artery disease: Preliminary findings of 3 months. J Clin Periodontol. 2019;46:321–31.

266. Saito A, Bizenjima T, Takeuchi T, Suzuki E, Sato M, Yoshikawa K, et al. Treatment of intrabony periodontal defects using rhFGF-2 in combination with deproteinized bovine bone mineral or rhFGF-2 alone: A 6-month randomized controlled trial. J Clin Periodontol. 2019;46:332–41.

267. Boven GC, Speksnijder CM, Meijer HJA, Vissink A, Raghoebar GM. Masticatory ability improves after maxillary implant overdenture treatment: A randomized controlled trial with 1-year follow-up. Clin Implant Dent Relat Res. 2019;21:369–76.

268. Hartlev J, Spin-Neto R, Schou S, Isidor F, Nørholt SE. Cone beam computed tomography evaluation of staged lateral ridge augmentation using platelet-rich fibrin or resorbable collagen membranes in a randomized controlled clinical trial. Clin Oral Implants Res. 2019;30:277–84.

269. Llanos AH, Sapata VM, Jung RE, Hämmerle CH, Thoma DS, César Neto JB, et al. Comparison between two bone substitutes for alveolar ridge preservation after tooth extraction: Cone-beam computed tomography results of a non-inferiority randomized controlled trial. J Clin Periodontol. 2019;46:373–81.

270. Pichotano EC, de Molon RS, de Souza RV, Austin RS, Marcantonio E, Zandim-Barcelos DL. Evaluation of L-PRF combined with deproteinized bovine bone mineral for early implant placement after maxillary sinus augmentation: A randomized clinical trial. Clin Implant Dent Relat Res. 2019;21:253–62.

271. Toia M, Stocchero M, Becktor JP, Chrcanovic B, Wennerberg A. Implant vs abutment level connection in implant supported screw-retained fixed partial dentures with cobalt-chrome framework: 1-year interim results of a randomized clinical study. Clin Implant Dent Relat Res. 2019;21:238–46.

272. Graziani F, Gennai S, Petrini M, Bettini L, Tonetti M. Enamel matrix derivative stabilizes blood clot and improves clinical healing in deep pockets after flapless periodontal therapy: A Randomized Clinical Trial. J Clin Periodontol. 2019;46:231–40.

273. Peres Pimentel S, Vieira Ribeiro F, Correa Casarin R, Ribeiro Cirano F, Haguihara Luchesi V, Gallego Arias Pecorari V, et al. Triclosan-containing fluoride toothpaste on clinical parameters and osteo-inflammatory mediators when applied in a stent during experimental peri-implant mucositis in smokers. Clin Oral Implants Res. 2019;30:187–95.

274. Joshi AA, Padhye AM, Gupta HS. Platelet derived growth factor-BB levels in gingival crevicular fluid of localized intrabony defect sites treated with platelet rich fibrin membrane or collagen membrane containing recombinant human platelet derived growth factor-BB: A randomized clinical and biochemical study. J Periodontol. 2019;90:701–8.

275. Slot W, Raghoebar GM, Cune MS, Vissink A, Meijer HJA. Four or six implants in the maxillary posterior region to support an overdenture: 5-year results from a randomized controlled trial. Clin Oral Implants Res. 2019;30:169–77.

276. Machtei EE, Mayer Y, Horwitz J, Zigdon-Giladi H. Prospective randomized controlled clinical trial to compare hard tissue changes following socket preservation using alloplasts, xenografts vs no grafting: Clinical and histological findings. Clin Implant Dent Relat Res. 2019;21:14–20.

277. Donos N, Horvath A, Calciolari E, Mardas N. Immediate provisionalization of bone level implants with a hydrophilic surface. A five-year follow-up of a randomized controlled clinical trial. Clin Oral Implants Res. 2019;30:139–49.

278. Younes F, Eghbali A, De Bruyckere T, Cleymaet R, Cosyn J. A randomized controlled trial on the efficiency of free-handed, pilot-drill guided and fully guided implant surgery in partially edentulous patients. Clin Oral Implants Res. 2019;30:131–8.

279. Pang K-M, Lee J-K, Choi S-H, Kim Y-K, Kim B-J, Lee J-H. Maxillary Sinus Augmentation With Calcium Phosphate Double-Coated Anorganic Bovine Bone: Comparative Multicenter Randomized Clinical Trial With Histological and Radiographic Evaluation. Implant Dent. 2019;28:39–45.

280. Abtahi J, Henefalk G, Aspenberg P. Impact of a zoledronate coating on early post-surgical implant stability and marginal bone resorption in the maxilla-A split-mouth randomized clinical trial. Clin Oral Implants Res. 2019;30:49–58.

281. Gürkan A, Tekdal GP, Bostancı N, Belibasakis GN. Cytokine, chemokine, and growth factor levels in peri-implant sulcus during wound healing and osseointegration after piezosurgical versus conventional implant site preparation: Randomized, controlled, split-mouth trial. J Periodontol. 2019;90:616–26.

282. Laass A, Sailer I, Hüsler J, Hämmerle CH, Thoma DS. Randomized Controlled Clinical Trial of All-Ceramic Single-Tooth Implant Reconstructions Using Modified Zirconia Abutments: Results at 5 Years After Loading. Int J Periodontics Restorative Dent. 2019;39:17–27.

283. Allocca G, Pudylyk D, Signorino F, Grossi GB, Maiorana C. Effectiveness and compliance of an oscillating-rotating toothbrush in patients with dental implants: a randomized clinical trial. Int J Implant Dent. 2018;4:38.

284. Weerapong K, Sirimongkolwattana S, Sastraruji T, Khongkhunthian P. Comparative study of immediate loading on short dental implants and conventional dental implants in the posterior mandible: A randomized clinical trial. Int J Oral Maxillofac Implants. 2019;34:141–9.

285. Oh J-S, Seo Y-S, Lee G-J, You J-S, Kim S-G. A Comparative Study with Biphasic Calcium Phosphate to Deproteinized Bovine Bone in Maxillary Sinus Augmentation: A Prospective Randomized and Controlled Clinical Trial. Int J Oral Maxillofac Implants. 2019;34:233–42.

286. Kawakami S, Lang NP, Ferri M, Apaza Alccayhuaman KA, Botticelli D. Influence of the height of the antrostomy in sinus floor elevation assessed by cone beam computed tomography- a randomized clinical trial. Int J Oral Maxillofac Implants. 2019;34:223–32.

287. Stavropoulou C, Atout RN, Brownlee M, Schroth RJ, Kelekis-Cholakis A. A randomized clinical trial of cyanoacrylate tissue adhesives in donor site of connective tissue grafts. J Periodontol. 2019;90:608–15.

288. Merli M, Moscatelli M, Mariotti G, Pagliaro U, Raffaelli E, Nieri M. Comparing membranes and bone substitutes in a one-stage procedure for horizontal bone augmentation. Three-year post-loading results of a double-blind randomised controlled trial. Eur J Oral Implantol. 2018;11:441–52.

289. Esposito M, Trullenque-Eriksson A, Tallarico M. Endodontic retreatment versus dental implants of teeth with an uncertain endodontic prognosis: 3-year results from a randomised controlled trial. Eur J Oral Implantol. 2018;11:423–38.

290. Meloni SM, Baldoni E, Duvina M, Pisano M, De Riu G, Tallarico M. Immediate non-occlusal versus delayed loading of mandibular first molars. Five-year results from a randomised controlled trial. Eur J Oral Implantol. 2018;11:409–18.

291. Todisco M, Sbricoli L, Ippolito DR, Esposito M. Do we need abutments at immediately loaded implants supporting cross-arch fixed prostheses? Results from a 5-year randomised controlled trial. Eur J Oral Implantol. 2018;11:397–407.

292. Felice P, Barausse C, Pistilli R, Ippolito DR, Esposito M. Short implants versus longer implants in vertically augmented posterior mandibles: result at 8 years after loading from a randomised controlled trial. Eur J Oral Implantol. 2018;11:385–95.

293. Maniewicz S, Duvernay E, Srinivasan M, Perneger T, Schimmel M, Müller F. Effect of implant-supported mandibular overdentures versus reline on masticatory performance and salivary flow rates in very old adults-A randomized clinical trial. Clin Oral Implants Res. 2019;30:59–67.

294. Bodhare GH, Kolte AP, Kolte RA, Shirke PY. Clinical and radiographic evaluation and comparison of bioactive bone alloplast morsels when used alone and in combination with platelet-rich fibrin in the treatment of periodontal intrabony defects-A randomized controlled trial. J Periodontol. 2019;90:584–94.

295. Bielemann AM, Marcello-Machado RM, Schuster AJ, Chagas Júnior OL, Del Bel Cury AA, Faot F. Healing differences in narrow diameter implants submitted to immediate and conventional loading in mandibular overdentures: A randomized clinical trial. J Periodontal Res. 2019;54:241–50.

296. Park E-J, Kwon E-Y, Kim H-J, Lee J-Y, Choi J, Joo J-Y. Clinical and microbiological effects of the supplementary use of an erythritol powder air-polishing device in non-surgical periodontal therapy: a randomized clinical trial. J Periodontal Implant Sci. 2018;48:295–304.

297. Sun D-J, Lim H-C, Lee D-W. Alveolar ridge preservation using an open membrane approach for sockets with bone deficiency: A randomized controlled clinical trial. Clin Implant Dent Relat Res. 2019;21:175–82.

298. Cadore UB, Reis MBL, Martins SHL, Invernici M de M, Novaes AB, Taba M, et al. Multiple sessions of antimicrobial photodynamic therapy associated with surgical periodontal treatment in patients with chronic periodontitis. J Periodontol. 2019;90:339–49.

299. de Albuquerque RF, Fromentin O, Lassauzay C, Conceição Pereira Saraiva M da. Patient satisfaction versus retention of implant overdentures with two attachment systems: A randomized trial. Clin Implant Dent Relat Res. 2019;21:21–31.

300. Hong HR, Chen C-Y, Kim DM, Machtei EE. Ridge preservation procedures revisited: A randomized controlled trial to evaluate dimensional changes with two different surgical protocols. J Periodontol. 2019;90:331–8.

301. Mau JL, Grodin E, Lin J-J, Chen MC-J, Ho C-H, Cochran D. A comparative, randomized, prospective, two-center clinical study to evaluate the clinical and esthetic outcomes of two different bone grafting techniques in early implant placement. J Periodontol. 2019;90:247–55.

302. Thoma DS, Haas R, Sporniak-Tutak K, Garcia A, Taylor TD, Hämmerle CHF. Randomized controlled multicentre study comparing short dental implants (6 mm) versus longer dental implants (11-15 mm) in combination with sinus floor elevation procedures: 5-Year data. J Clin Periodontol. 2018;45:1465–74.

303. Thoma DS, Sailer I, Mühlemann S, Gil A, Jung RE, Hämmerle CHF. Randomized controlled clinical study of veneered zirconia abutments for single implant crowns: Clinical, histological, and microbiological outcomes. Clin Implant Dent Relat Res. 2018;20:988–96.

304. Stewart B, Shibli JA, Araujo M, Figueiredo LC, Panagakos F, Matarazzo F, et al. Effects of a toothpaste containing 0.3% triclosan in the maintenance phase of peri-implantitis treatment: 2-Year randomized clinical trial. Clin Oral Implants Res. 2018;29:973–85.

305. Kruse AB, Akakpo DL, Maamar R, Woelber JP, Al-Ahmad A, Vach K, et al. Trehalose powder for subgingival air-polishing during periodontal maintenance therapy: A randomized controlled trial. J Periodontol. 2019;90:263–70.

306. Marconcini S, Giammarinaro E, Derchi G, Alfonsi F, Covani U, Barone A. Clinical outcomes of implants placed in ridge-preserved versus nonpreserved sites: A 4-year randomized clinical trial. Clin Implant Dent Relat Res. 2018;20:906–14.

307. Rokn AR, Monzavi A, Panjnoush M, Hashemi HM, Kharazifard MJ, Bitaraf T. Comparing 4-mm dental implants to longer implants placed in augmented bones in the atrophic posterior mandibles: One-year results of a randomized controlled trial. Clin Implant Dent Relat Res. 2018;20:997–1002.

308. Tavelli L, Asa’ad F, Acunzo R, Pagni G, Consonni D, Rasperini G. Minimizing Patient Morbidity Following Palatal Gingival Harvesting: A Randomized Controlled Clinical Study. Int J Periodontics Restorative Dent. 2018;38:e127–34.

309. El-Sharkawy H, Elmeadawy S, Elshinnawi U, Anees M. Is dietary melatonin supplementation a viable adjunctive therapy for chronic periodontitis?-A randomized controlled clinical trial. J Periodontal Res. 2019;54:190–7.

310. Ranaan J, Bassir SH, Andrada L, Shamshiri AR, Maksoud M, Raanan R, et al. Clinical efficacy of the graft free slit-window sinus floor elevation procedure: A 2-year randomized controlled clinical trial. Clin Oral Implants Res. 2018;29:1107–19.

311. Lago L, da Silva L, Martinez-Silva I, Rilo B. Radiographic Assessment Of Crestal Bone Loss In Tissue-Level Implants Restored By Platform Matching Compared With Bone-Level Implants Restored By Platform Switching: A Randomized, Controlled, Split-Mouth Trial With 3-Year Follow-Up. Int J Oral Maxillofac Implants. 2019;34:179–86.

312. Mastrangelo F, Gastaldi G, Vinci R, Troiano G, Tettamanti L, Gherlone E, et al. Immediate Postextractive Implants With and Without Bone Graft: 3-year Follow-up Results From a Multicenter Controlled Randomized Trial. Implant Dent. 2018;27:638–45.

313. Borges T, Leitão B, Pereira M, Carvalho Á, Galindo-Moreno P. Influence of the abutment height and connection timing in early peri-implant marginal bone changes: A prospective randomized clinical trial. Clin Oral Implants Res. 2018;29:907–14.

314. Meloni SM, Baldoni E, Pisano M, Tullio A, De Riu G, Tallarico M. 1-year results from a split-mouth randomised controlled pilot trial comparing implants with 0.75 mm of machined collar placed at bone level or supracrestally. Eur J Oral Implantol. 2018;11:353–9.

315. Abi-Aad H, Daher F, Dimassi H, Cordioli G, Majzoub Z. Immediate vs conventional loading of variable-thread tapered implants supporting three- to four-unit fixed partial dentures in the posterior maxilla: 1-year interim results of a split-mouth randomised controlled trial. Eur J Oral Implantol. 2018;11:337–50.

316. Cannizzaro G, Cavallari M, Lazzarini M, Purello D’ambrosio G, Scialpi G, Audino S, et al. Immediate loading of three (fixed-on-3) vs four (fixed-on-4) implants supporting cross-arch fixed prostheses: 1-year results from a multicentre randomised controlled trial. Eur J Oral Implantol. 2018;11:323–33.

317. Esposito M, Grufferty B, Papavasiliou G, Dominiak M, Trullenque-Eriksson A, Heinemann F. Immediate loading of occluding definitive partial fixed prostheses vs non-occluding provisional restorations - 3-year post-loading results from a pragmatic multicentre randomised controlled trial. Eur J Oral Implantol. 2018;11:309–20.

318. Cannizzaro G, Felice P, Ippolito DR, Velasco-Ortega E, Esposito M. Immediate loading of fixed cross-arch prostheses supported by flapless-placed 5 mm or 11.5 mm long implants: 5-year results from a randomised controlled trial. Eur J Oral Implantol. 2018;11:295–306.

319. Storelli S, Abbà A, Scanferla M, Botticelli D, Romeo E. 6 mm vs 10 mm-long implants in the rehabilitation of posterior jaws: A 10-year follow-up of a randomised controlled trial. Eur J Oral Implantol. 2018;11:283–92.

320. Iero PT, Mulherin DR, Jensen O, Berry T, Danesi H, Razook SJ. A Prospective, Randomized, Open-Label Study Comparing an Opioid-Sparing Postsurgical Pain Management Protocol With and Without Liposomal Bupivacaine for Full-Arch Implant Surgery. Int J Oral Maxillofac Implants. 2018;33:1155–64.

321. Barwacz CA, Stanford CM, Diehl UA, Cooper LF, Feine J, McGuire M, et al. Pink Esthetic Score Outcomes Around Three Implant-Abutment Configurations: 3-Year Results. Int J Oral Maxillofac Implants. 2018;33:1126–35.

322. ElSyad MA, Denewar BA, Elsaih EA. Clinical and Radiographic Evaluation of Bar, Telescopic, and Locator Attachments for Implant-Stabilized Overdentures in Patients with Mandibular Atrophied Ridges: A Randomized Controlled Clinical Trial. Int J Oral Maxillofac Implants. 2018;33:1103–11.

323. Schnutenhaus S, Doering I, Dreyhaupt J, Rudolph H, Luthardt RG. Alveolar ridge preservation with a collagen material: a randomized controlled trial. J Periodontal Implant Sci. 2018;48:236–50.

324. Shim J-Y, Lee Y, Lim J-H, Jin M-U, Lee J-M, Suh J-Y, et al. Comparative Evaluation of Recombinant Human Bone Morphogenetic Protein-2/Hydroxyapatite and Bovine Bone for New Bone Formation in Alveolar Ridge Preservation. Implant Dent. 2018;27:623–9.

325. Corning PJ, Mealey BL. Ridge preservation following tooth extraction using mineralized freeze-dried bone allograft compared to mineralized solvent-dehydrated bone allograft: A randomized controlled clinical trial. J Periodontol. 2019;90:126–33.

326. Joda T, Ferrari M, Bragger U, Zitzmann NU. Patient Reported Outcome Measures (PROMs) of posterior single-implant crowns using digital workflows: A randomized controlled trial with a three-year follow-up. Clin Oral Implants Res. 2018;29:954–61.

327. Vergnes J-N, Canceill T, Vinel A, Laurencin-Dalicieux S, Maupas-Schwalm F, Blasco-Baqué V, et al. The effects of periodontal treatment on diabetic patients: The DIAPERIO randomized controlled trial. J Clin Periodontol. 2018;45:1150–63.

328. De Bruyckere T, Eeckhout C, Eghbali A, Younes F, Vandekerckhove P, Cleymaet R, et al. A randomized controlled study comparing guided bone regeneration with connective tissue graft to re-establish convexity at the buccal aspect of single implants: A one-year CBCT analysis. J Clin Periodontol. 2018;45:1375–87.

329. Isler SC, Soysal F, Ceyhanlı T, Bakırarar B, Unsal B. Regenerative surgical treatment of peri-implantitis using either a collagen membrane or concentrated growth factor: A 12-month randomized clinical trial. Clin Implant Dent Relat Res. 2018;20:703–12.

330. Schneider D, Sancho-Puchades M, Benic GI, Hämmerle CH, Jung RE. A Randomized Controlled Clinical Trial Comparing Conventional and Computer-Assisted Implant Planning and Placement in Partially Edentulous Patients. Part 1: Clinician-Related Outcome Measures. Int J Periodontics Restorative Dent. 2018;38 Suppl:s49–57.

331. Eisner B, Naenni N, Hüsler J, Hämmerle C, Thoma D, Sailer I. Three-Year Results of a Randomized Controlled Clinical Trial Using Submucosally Veneered and Unveneered Zirconia Abutments Supporting All-Ceramic Single-Implant Crowns. Int J Periodontics Restorative Dent. 2018;38:645–52.

332. Vianna TT, Taiete T, Casarin RCV, Giorgi MCC, Aguiar FHB, Silvério KG, et al. Evaluation of peri-implant marginal tissues around tissue-level and bone-level implants in patients with a history of chronic periodontitis. J Clin Periodontol. 2018;45:1255–65.

333. Clementini M, Discepoli N, Danesi C, de Sanctis M. Biologically guided flap stability: the role of flap thickness including periosteum retention on the performance of the coronally advanced flap-A double-blind randomized clinical trial. J Clin Periodontol. 2018;45:1238–46.

334. Invernici MM, Salvador SL, Silva PHF, Soares MSM, Casarin R, Palioto DB, et al. Effects of Bifidobacterium probiotic on the treatment of chronic periodontitis: A randomized clinical trial. J Clin Periodontol. 2018;45:1198–210.

335. Threeburuth W, Aunmeungtong W, Khongkhunthian P. Comparison of immediate-load mini dental implants and conventional-size dental implants to retain mandibular Kennedy class I removable partial dentures: A randomized clinical trial. Clin Implant Dent Relat Res. 2018;20:785–92.

336. Theodoro LH, Rocha GS, Ribeiro Junior VL, Sakakura CE, de Mello Neto JM, Garcia VG, et al. Bone Formed After Maxillary Sinus Floor Augmentation by Bone Autografting With Hydroxyapatite and Low-Level Laser Therapy: A Randomized Controlled Trial With Histomorphometrical and Immunohistochemical Analyses. Implant Dent. 2018;27:547–54.

337. Lee J-S, Cha J-K, Kim C-S. Alveolar ridge regeneration of damaged extraction sockets using deproteinized porcine versus bovine bone minerals: A randomized clinical trial. Clin Implant Dent Relat Res. 2018;20:729–37.

338. Glibert M, Matthys C, Maat R-J, De Bruyn H, Vervaeke S. A randomized controlled clinical trial assessing initial crestal bone remodeling of implants with a different surface roughness. Clin Implant Dent Relat Res. 2018;20:824–8.

339. Nguyen SV, Nguyen MTH, Tran BC, Ho MTQ, Umeda K, Rahman S. Evaluation of lozenges containing egg yolk antibody against Porphyromonas gingivalis gingipains as an adjunct to conventional non-surgical therapy in periodontitis patients: A randomized controlled clinical trial. J Periodontol. 2018;89:1334–9.

340. Chowdhary R, Kumararama SS. “Simpli5y” a noval concept for fixed rehabilitation of completely edentulous maxillary and mandibular edentulous arches: A 3-year randomized clinical trial, supported by a numerical analysis. Clin Implant Dent Relat Res. 2018;20:749–55.

341. Li X, Tang L, Lin YF, Xie GF. Role of vitamin C in wound healing after dental implant surgery in patients treated with bone grafts and patients with chronic periodontitis. Clin Implant Dent Relat Res. 2018;20:793–8.

342. Lima RG, Lima TG, Francischone CE, Turssi C, Souza Picorelli Assis NM, Sotto-Maior BS. Bone Volume Dynamics and Implant Placement Torque in Horizontal Bone Defects Reconstructed with Autologous or Xenogeneic Block Bone: A Randomized, Controlled, Split-Mouth, Prospective Clinical Trial. Int J Oral Maxillofac Implants. 2018;33:888–94.

343. Quintero AJ, Chaparro A, Quirynen M, Ramirez V, Prieto D, Morales H, et al. Effect of two periodontal treatment modalities in patients with uncontrolled type 2 diabetes mellitus: A randomized clinical trial. J Clin Periodontol. 2018;45:1098–106.

344. El Hadidy MS, Mounir M, Abou-Elfetouh A, Barakat A. Assessment of vertical ridge augmentation and labial prominence using buccal versus palatal approaches for maxillary segmental sandwich osteotomy (inlay technique): A randomized clinical trial. Clin Implant Dent Relat Res. 2018;20:722–8.

345. Isler SC, Eraydin N, Akkale H, Ozdemir B. Oral flurbiprofen spray for mucosal graft harvesting at the palatal area: A randomized placebo-controlled study. J Periodontol. 2018;89:1174–83.

346. Tawfik OK, Naiem SN, Tawfik LK, Yussif N, Meghil MM, Cutler CW, et al. Lip repositioning with or without myotomy: A randomized clinical trial. J Periodontol. 2018;89:815–23.

347. Zadeh HH, Guljé F, Palmer PJ, Abrahamsson I, Chen S, Mahallati R, et al. Marginal bone level and survival of short and standard-length implants after 3 years: An Open Multi-Center Randomized Controlled Clinical Trial. Clin Oral Implants Res. 2018;29:894–906.

348. Renvert S, Roos-Jansåker A-M, Persson GR. Surgical treatment of peri-implantitis lesions with or without the use of a bone substitute-a randomized clinical trial. J Clin Periodontol. 2018;45:1266–74.

349. Farina R, Franceschetti G, Travaglini D, Consolo U, Minenna L, Schincaglia GP, et al. Morbidity following transcrestal and lateral sinus floor elevation: A randomized trial. J Clin Periodontol. 2018;45:1128–39.

350. Laky M, Anscheringer I, Wolschner L, Heber S, Haririan H, Schrottmaier WC, et al. Periodontal treatment limits platelet activation in patients with periodontitis-a controlled-randomized intervention trial. J Clin Periodontol. 2018;45:1090–7.

351. Sapata VM, Sanz-Martín I, Hämmerle CHF, Cesar Neto JB, Jung RE, Thoma DS. Profilometric changes of peri-implant tissues over 5 years: A randomized controlled trial comparing a one- and two-piece implant system. Clin Oral Implants Res. 2018;29:864–72.

352. Bartols A, Kasprzyk S, Walther W, Korsch M. Lateral alveolar ridge augmentation with autogenous block grafts fixed at a distance versus resorbable Poly-D-L-Lactide foil fixed at a distance: A single-blind, randomized, controlled trial. Clin Oral Implants Res. 2018;29:843–54.

353. Maino GNE, Valles C, Santos A, Pascual A, Esquinas C, Nart J. Influence of suturing technique on wound healing and patient morbidity after connective tissue harvesting. A randomized clinical trial. J Clin Periodontol. 2018;45:977–85.

354. Fischer KR, Mühlemann S, Jung RE, Friedmann A, Fickl S. Dimensional Evaluation of Different Ridge Preservation Techniques with a Bovine Xenograft: A Randomized Controlled Clinical Trial. Int J Periodontics Restorative Dent. 2018;38:549–56.

355. Temmerman A, Cleeren GJ, Castro AB, Teughels W, Quirynen M. L-PRF for increasing the width of keratinized mucosa around implants: A split-mouth, randomized, controlled pilot clinical trial. J Periodontal Res. 2018;53:793–800.

356. Tallarico M, Esposito M, Xhanari E, Caneva M, Meloni SM. Computer-guided vs freehand placement of immediately loaded dental implants: 5-year postloading results of a randomised controlled trial. Eur J Oral Implantol. 2018;11:203–13.

357. Felice P, Barausse C, Pistilli V, Piattelli M, Ippolito DR, Esposito M. Posterior atrophic jaws rehabilitated with prostheses supported by 6 mm long × 4 mm wide implants or by longer implants in augmented bone. 3-year post-loading results from a randomised controlled trial. Eur J Oral Implantol. 2018;11:175–87.

358. Cannizzaro G, Felice P, Trullenque-Eriksson A, Lazzarini M, Velasco-Ortega E, Esposito M. Immediate vs early loading of 6.6 mm flapless-placed single implants: 9 years after-loading report of a split-mouth randomised controlled trial. Eur J Oral Implantol. 2018;11:163–73.

359. Davó R, Felice P, Pistilli R, Barausse C, Marti-Pages C, Ferrer-Fuertes A, et al. Immediately loaded zygomatic implants vs conventional dental implants in augmented atrophic maxillae: 1-year post-loading results from a multicentre randomised controlled trial. Eur J Oral Implantol. 2018;11:145–61.

360. van Nimwegen WG, Raghoebar GM, Zuiderveld EG, Jung RE, Meijer HJA, Mühlemann S. Immediate placement and provisionalization of implants in the aesthetic zone with or without a connective tissue graft: A 1-year randomized controlled trial and volumetric study. Clin Oral Implants Res. 2018;29:671–8.

361. Pankaj D, Sahu I, Kurian IG, Pradeep AR. Comparative evaluation of subgingivally delivered 1.2% rosuvastatin and 1% metformin gel in treatment of intrabony defects in chronic periodontitis: A randomized controlled clinical trial. J Periodontol. 2018;89:1318–25.

362. Ferrarotti F, Romano F, Gamba MN, Quirico A, Giraudi M, Audagna M, et al. Human intrabony defect regeneration with micrografts containing dental pulp stem cells: A randomized controlled clinical trial. J Clin Periodontol. 2018;45:841–50.

363. Rasperini G, Acunzo R, Pellegrini G, Pagni G, Tonetti M, Pini Prato GP, et al. Predictor factors for long-term outcomes stability of coronally advanced flap with or without connective tissue graft in the treatment of single maxillary gingival recessions: 9 years results of a randomized controlled clinical trial. J Clin Periodontol. 2018;45:1107–17.

364. Jokstad A, Winnett B, Fava J, Powell D, Somogyi-Ganss E. Investigational Clinical Trial of a Prototype Optoelectronic Computer-Aided Navigation Device for Dental Implant Surgery. Int J Oral Maxillofac Implants. 2018;33:679–92.

365. Amaliya A, Risdiana AS, Van der Velden U. Effect of guava and vitamin C supplementation on experimental gingivitis: A randomized clinical trial. J Clin Periodontol. 2018;45:959–67.

366. Zuiderveld EG, Meijer HJA, Vissink A, Raghoebar GM. The influence of different soft-tissue grafting procedures at single implant placement on esthetics: A randomized controlled trial. J Periodontol. 2018;89:903–14.

367. Mazhari F, Boskabady M, Moeintaghavi A, Habibi A. The effect of toothbrushing and flossing sequence on interdental plaque reduction and fluoride retention: A randomized controlled clinical trial. J Periodontol. 2018;89:824–32.

368. Schwarz F, Becker J, Civale S, Sahin D, Iglhaut T, Iglhaut G. Influence of the width of keratinized tissue on the development and resolution of experimental peri-implant mucositis lesions in humans. Clin Oral Implants Res. 2018;29:576–82.

369. Clark D, Rajendran Y, Paydar S, Ho S, Cox D, Ryder M, et al. Advanced platelet-rich fibrin and freeze-dried bone allograft for ridge preservation: A randomized controlled clinical trial. J Periodontol. 2018;89:379–87.

370. Santamaria MP, Silveira CA, Mathias IF, Neves FL da S, Dos Santos LM, Jardini MAN, et al. Treatment of single maxillary gingival recession associated with non-carious cervical lesion: Randomized clinical trial comparing connective tissue graft alone to graft plus partial restoration. J Clin Periodontol. 2018;45:968–76.

371. Basler T, Naenni N, Schneider D, Hämmerle CHF, Jung RE, Thoma DS. Randomized controlled clinical study assessing two membranes for guided bone regeneration of peri-implant bone defects: 3-year results. Clin Oral Implants Res. 2018;29:499–507.

372. Hutton CG, Johnson GK, Barwacz CA, Allareddy V, Avila-Ortiz G. Comparison of two different surgical approaches to increase peri-implant mucosal thickness: A randomized controlled clinical trial. J Periodontol. 2018;89:807–14.

373. Raes M, D’hondt R, Teughels W, Coucke W, Quirynen M. A 5-year randomized clinical trial comparing minimally with moderately rough implants in patients with severe periodontitis. J Clin Periodontol. 2018;45:711–20.

374. Utyuzh AS, Yumashev AV, Lang HW, Zekiy AO, Lushkov RM. Comprehensive Treatment and Rehabilitation of Patients With Osteosarcoma of the Mandible. Implant Dent. 2018;27:332–41.

375. Younes F, Cosyn J, De Bruyckere T, Cleymaet R, Bouckaert E, Eghbali A. A randomized controlled study on the accuracy of free-handed, pilot-drill guided and fully guided implant surgery in partially edentulous patients. J Clin Periodontol. 2018;45:721–32.

376. Jung RE, Sapata VM, Hämmerle CHF, Wu H, Hu X-L, Lin Y. Combined use of xenogeneic bone substitute material covered with a native bilayer collagen membrane for alveolar ridge preservation: A randomized controlled clinical trial. Clin Oral Implants Res. 2018;29:522–9.

377. Fouad W, Osman A, Atef M, Hakam M. Guided maxillary sinus floor elevation using deproteinized bovine bone versus graftless Schneiderian membrane elevation with simultaneous implant placement: Randomized clinical trial. Clin Implant Dent Relat Res. 2018;20:424–33.

378. Glibert M, Vervaeke S, Jacquet W, Vermeersch K, Östman P-O, De Bruyn H. A randomized controlled clinical trial to assess crestal bone remodeling of four different implant designs. Clin Implant Dent Relat Res. 2018;20:455–62.

379. Isehed C, Svenson B, Lundberg P, Holmlund A. Surgical treatment of peri-implantitis using enamel matrix derivative, an RCT: 3- and 5-year follow-up. J Clin Periodontol. 2018;45:744–53.

380. Donati M, Ekestubbe A, Lindhe J, Wennström JL. Marginal bone loss at implants with different surface characteristics - A 20-year follow-up of a randomized controlled clinical trial. Clin Oral Implants Res. 2018;29:480–7.

381. Nunes FAS, Pignaton TB, Novaes AB, Taba M, Messora MR, Palioto DB, et al. Evaluation of a bone substitute covered with a collagen membrane for ridge preservation after tooth extraction. Clinical and tomographic randomized controlled study in humans. Clin Oral Implants Res. 2018;29:424–33.

382. Esposito M, Cardaropoli D, Gobbato L, Scutellà F, Fabianelli A, Mascellani S, et al. The role of dental implant abutment design on the aesthetic outcome: preliminary 3-month post-loading results from a multicentre split-mouth randomised controlled trial comparing two different abutment designs. Eur J Oral Implantol. 2018;11:77–87.

383. Mitsias M, Siormpas K, Pistilli V, Trullenque-Eriksson A, Esposito M. Immediate, early (6 weeks) and delayed loading (3 months) of single, partial and full fixed implant supported prostheses: 1-year post-loading data from a multicentre randomised controlled trial. Eur J Oral Implantol. 2018;11:63–75.

384. Gastaldi G, Felice P, Pistilli V, Barausse C, Ippolito DR, Esposito M. Posterior atrophic jaws rehabilitated with prostheses supported by 5 × 5 mm implants with a nanostructured calcium-incorporated titanium surface or by longer implants in augmented bone. 3-year results from a randomised controlled trial. Eur J Oral Implantol. 2018;11:49–61.

385. Bolle C, Felice P, Barausse C, Pistilli V, Trullenque-Eriksson A, Esposito M. 4 mm long vs longer implants in augmented bone in posterior atrophic jaws: 1-year post-loading results from a multicentre randomised controlled trial. Eur J Oral Implantol. 2018;11:31–47.

386. Esposito M, Davó R, Marti-Pages C, Ferrer-Fuertes A, Barausse C, Pistilli R, et al. Immediately loaded zygomatic implants vs conventional dental implants in augmented atrophic maxillae: 4 months post-loading results from a multicentre randomised controlled trial. Eur J Oral Implantol. 2018;11:11–28.

387. Arabaci T, Albayrak M. Titanium-prepared platelet-rich fibrin provides advantages on periodontal healing: A randomized split-mouth clinical study. J Periodontol. 2018;89:255–64.

388. Xu Y, Selerio-Poely T, Ye X. Clinical and microbiological effects of egg yolk antibody against Porphyromonas gingivalis as an adjunct in the treatment of moderate to severe chronic periodontitis: a randomized placebo-controlled clinical trial. J Periodontal Implant Sci. 2018;48:47–59.

389. Park S-H, Cho S-H, Han J-Y. Effective professional intraoral tooth brushing instruction using the modified plaque score: a randomized clinical trial. J Periodontal Implant Sci. 2018;48:22–33.

390. Lago L, da Silva L, Martinez-Silva I, Rilo B. Crestal Bone Level Around Tissue-Level Implants Restored with Platform Matching and Bone-Level Implants Restored with Platform Switching: A 5-Year Randomized Controlled Trial. Int J Oral Maxillofac Implants. 2018;33:448–56.

391. Hanser T, Doliveux R. MicroSaw and Piezosurgery in Harvesting Mandibular Bone Blocks from the Retromolar Region: A Randomized Split-Mouth Prospective Clinical Trial. Int J Oral Maxillofac Implants. 2018;33:365–72.

392. Ribeiro FV, Casati MZ, Casarin RC, Corrêa MG, Cirano FR, Negri BM, et al. Impact of a triclosan-containing toothpaste during the progression of experimental peri-implant mucositis: Clinical parameters and local pattern of osteo-immunoinflammatory mediators in peri-implant fluid. J Periodontol. 2018;89:203–12.

393. Schlee M, Rathe F, Bommer C, Bröseler F, Kind L. Self-assembling peptide matrix for treatment of dentin hypersensitivity: A randomized controlled clinical trial. J Periodontol. 2018;89:653–60.

394. Graziani F, Discepoli N, Gennai S, Karapetsa D, Nisi M, Bianchi L, et al. The effect of twice daily kiwifruit consumption on periodontal and systemic conditions before and after treatment: A randomized clinical trial. J Periodontol. 2018;89:285–93.

395. Froum SJ, Cho S-C, Suzuki T, Yu P, Corby P, Khouly I. Epicrestal and subcrestal placement of platform-switched implants: 18 month-result of a randomized, controlled, split-mouth, prospective clinical trial. Clin Oral Implants Res. 2018;29:353–66.

396. Girbés-Ballester P, Viña-Almunia J, Balaguer-Martí JC, Peñarrocha-Diago M, Peñarrocha-Oltra D. Effect of incision design on interproximal bone loss of teeth adjacent to single implants. A randomized controlled clinical trial comparing intrasulcular vs paramarginal incision. Clin Oral Implants Res. 2018;29:367–74.

397. Bernardi S, Gatto R, Severino M, Botticelli G, Caruso S, Rastelli C, et al. Short Versus Longer Implants in Mandibular Alveolar Ridge Augmented Using Osteogenic Distraction: One-Year Follow-up of a Randomized Split-Mouth Trial. J Oral Implantol. 2018;44:184–91.

398. Marconcini S, Giammarinaro E, Toti P, Alfonsi F, Covani U, Barone A. Longitudinal analysis on the effect of insertion torque on delayed single implants: A 3-year randomized clinical study. Clin Implant Dent Relat Res. 2018;20:322–32.

399. Sánchez-Pérez A, Muñoz-Peñalver J, Moya-Villaescusa MJ, Sánchez-Matás C. Effects of the Preoperative Administration of Dexketoprofen Trometamol on Pain and Swelling After Implant Surgery: A Randomized, Double-Blind Controlled Trial. J Oral Implantol. 2018;44:122–9.

400. Galofré M, Palao D, Vicario M, Nart J, Violant D. Clinical and microbiological evaluation of the effect of Lactobacillus reuteri in the treatment of mucositis and peri-implantitis: A triple-blind randomized clinical trial. J Periodontal Res. 2018;53:378–90.

401. Zygogiannis K, Aartman IH, Wismeijer D. Implant Mandibular Overdentures Retained by Immediately Loaded Implants: A 1-Year Randomized Trial Comparing Patient-Based Outcomes Between Mini Dental Implants and Standard-Sized Implants. Int J Oral Maxillofac Implants. 2018;33:197–205.

402. Rojo E, Stroppa G, Sanz-Martin I, Gonzalez-Martín O, Alemany AS, Nart J. Soft tissue volume gain around dental implants using autogenous subepithelial connective tissue grafts harvested from the lateral palate or tuberosity area. A randomized controlled clinical study. J Clin Periodontol. 2018;45:495–503.

403. Pietruska M, Skurska A, Podlewski Ł, Milewski R, Pietruski J. Clinical evaluation of Miller class I and II recessions treatment with the use of modified coronally advanced tunnel technique with either collagen matrix or subepithelial connective tissue graft: A randomized clinical study. J Clin Periodontol. 2019;46:86–95.

404. de Oliveira Fernandes G, Santos N, de Sousa M, Fernandes J. Liquid Platelet-Rich Fibrin Coating Implant Surface to Enhance Osseointegration: A Double-Blinded, Randomized Split-Mouth Trial with 1-Year Follow-up. Int J Oral Maxillofac Implants. 2022;37:159–70.

405. Barausse C, Pistilli R, Canullo L, Bonifazi L, Ferri A, Felice P. A 5‐year randomized controlled clinical trial comparing 4‐mm ultrashort to longer implants placed in regenerated bone in the posterior atrophic jaw. Clin Implant Dent Rel Res. 2022;24:4–12.

406. Gur AT, Guncu GN, Akman AC, Pinar A, Karabulut E, Nohutcu RM. Evaluation of GCF IL‐17, IL‐10, TWEAK, and sclerostin levels after scaling and root planing and adjunctive use of diode laser application in patients with periodontitis. Journal of Periodontology. 2022;:JPER.21-0494.

407. Eeckhout C, Ackerman J, Glibert M, Cosyn J. A randomized controlled trial evaluating hyaluronic acid gel as wound healing agent in alveolar ridge preservation. J Clinic Periodontology. 2022;49:280–91.

408. Jung RE, Kovacs MN, Thoma DS, Hämmerle CHF. Informative title: Guided bone regeneration with and without rhBMP‐2: 17‐year results of a randomized controlled clinical trial. Clinical Oral Implants Res. 2022;33:302–12.

409. Markovic A, Mišić T, Janjić B, Šćepanović M, Trifković B, Ilić B, et al. Immediate Vs. Early Loading of Bone Level Tapered Dental Implants with Hydrophilic Surface in Fully Edentulous Maxilla:Clinical and Patient-Centered Outcomes. Journal of Oral Implantology. 2021. https://doi.org/10.1563/aaid-joi-D-21-00045.

410. Kunavisarut C, Santivitoonvong A, Chaikantha S, Pornprasertsuk‐Damrongsri S, Joda T. Patient‐reported outcome measures comparing static computer‐aided implant surgery and conventional implant surgery for single‐tooth replacement: A randomized controlled trial. Clinical Oral Implants Res. 2022;33:278–90.

411. Tabassum A, Kazmi F, Wismeijer D, Siddiqui I, Tahmaseb A. A Prospective Randomized Clinical Trial on Radiographic Crestal Bone Loss Around Dental Implants Placed Using Two Different Drilling Protocols: 12-Month Follow-up. Int J Oral Maxillofac Implants. 2021;36:e175–82.

412. Guldiken I, Gurler G, Delilbasi C. Comparison of Dexmedetomidine and Midazolam in Conscious Sedation During Dental Implant Surgery: A Randomized Clinical Trial. Int J Oral Maxillofac Implants. 2021;36:e159–65.

413. Serrano B, Sanz‐Sánchez I, Serrano K, Montero E, Sanz M. One‐year outcomes of dental implants with a hybrid surface macro‐design placed in patients with history of periodontitis: A randomized clinical trial. J Clinic Periodontology. 2022;49:90–100.

414. Schlagenhauf U, Hess JV, Stölzel P, Haubitz I, Jockel‐Schneider Y. Impact of a two‐stage subgingival instrumentation scheme involving air polishing on attachment gain after active periodontal therapy. Journal of Periodontology. 2022;:JPER.21-0351.

415. Nielsen HB, Schou S, Bruun NH, Starch‐Jensen T. Professional and patient‐reported outcomes of two surgical approaches for implant‐supported single‐crown restoration: 1‐year results of a randomized controlled clinical trial. Clinical Oral Implants Res. 2022;33:197–208.

416. Lin S, Li X, Liu H, Wu F, Yang L, Su Y, et al. Clinical applications of concentrated growth factors combined with bone substitutes for alveolar ridge preservation in maxillary molar area: a randomized controlled trial. Int J Implant Dent. 2021;7:115.

417. Bartha V, Exner L, Schweikert D, Woelber JP, Vach K, Meyer A, et al. Effect of the Mediterranean diet on gingivitis: A randomized controlled trial. J Clinic Periodontology. 2022;49:111–22.

418. Hentenaar DFM, De Waal YCM, Stewart RE, Van Winkelhoff AJ, Meijer HJA, Raghoebar GM. Erythritol air polishing in the surgical treatment of peri‐implantitis: A randomized controlled trial. Clinical Oral Implants Res. 2022;33:184–96.

419. Papace C, Büsch C, Ristow O, Keweloh M, Hoffmann J, Mertens C. The effect of different soft-tissue management techniques for alveolar ridge preservation: a randomized controlled clinical trial. Int J Implant Dent. 2021;7:113.

420. Blanco C, Pico A, Dopico J, Gándara P, Blanco J, Liñares A. Adjunctive benefits of systemic metronidazole on non‐surgical treatment of peri‐implantitis. A randomized placebo‐controlled clinical trial. J Clinic Periodontology. 2022;49:15–27.

421. Hussain B, Karaca EO, Kuru BE, Gursoy H, Haugen HJ, Wohlfahrt JC. Treatment of residual pockets using an oscillating chitosan device versus regular curettes alone—A randomized, feasibility parallel‐arm clinical trial. Journal of Periodontology. 2022;93:780–9.

422. Anoixiadou S, Parashis A, Vouros I. Enamel matrix derivative as an adjunct to minimally invasive non‐surgical treatment of intrabony defects: A randomized clinical trial. J Clinic Periodontology. 2022;49:134–43.

423. Silva CGB, Sapata VM, Llanos AH, Romano MM, Jung RE, Hämmerle CHF, et al. Peri‐implant tissue changes at sites treated with alveolar ridge preservation in the aesthetic zone: Twenty‐two months follow‐up of a randomized clinical trial. J Clinic Periodontology. 2022;49:39–47.

424. Shuster A, Kleinman S, Reiser V, Ianculovici C, Peleg O, Ben-Ami R. Short Versus Extended Antibiotic Prophylaxis for Maxillary Sinus Floor Augmentation Via a Lateral Window Approach: A Randomized Controlled Trial. Int J Oral Maxillofac Implants. 2021;36:992–8.

425. Wang L, Wang T, Lu Y, Fan Z. Comparing the Clinical Outcome of Peri-implant Hard and Soft Tissue Treated with Immediate Individualized CAD/CAM Healing Abutments and Conventional Healing Abutments for Single-Tooth Implants in Esthetic Areas Over 12 Months: A Randomized Clinical Trial. Int J Oral Maxillofac Implants. 2021;36:977–84.

426. Enkling N, Kokoschka F, Schumacher D, Kraus D, Schimmel M, Abou‐Ayash S. Influence of the loading protocol and platform switching in two‐implant bar‐retained overdentures: 3‐year results from a randomized controlled equivalence clinical trial. Clinical Oral Implants Res. 2022;33:120–9.

427. Rathe F, Junker R, Gröger S, Meyle J, Schlee M. Inflammatory effects of individualized abutments bonded onto t itanium base on peri‐implant tissue health: A randomized controlled clinical trial. Clin Implant Dent Rel Res. 2021;23:874–82.

428. Philip J, Buijs MJ, Pappalardo VY, Crielaard W, Brandt BW, Zaura E. The microbiome of dental and peri‐implant subgingival plaque during peri‐implant mucositis therapy: A randomized clinical trial. J Clinic Periodontology. 2022;49:28–38.

429. Lie SAN, Leung CAW, Claessen RMMA, Merten H-A, Kessler PAWH. Implant survival after graftless sinus floor augmentation in highly atrophic maxillae: a randomized controlled trial in a split mouth study. Int J Implant Dent. 2021;7:107.

430. Solonko M, Regidor E, Ortiz‐Vigón A, Montero E, Vilchez B, Sanz M. Efficacy of keratinized mucosal augmentation with a collagen matrix concomitant to the surgical treatment of peri‐implantitis: A dual‐center randomized clinical trial. Clinical Oral Implants Res. 2022;33:105–19.

431. Toia M, Stocchero M, Galli S, Papia E, Wennerberg A, Becktor JP. The use of implant‐level connection in screw‐retained fixed partial dentures: A 3‐year randomised clinical trial. Clinical Oral Implants Res. 2022;33:78–93.

432. Feng C, Ding Y, Tang L, Gui Y, Shen X, He L, et al. Adjunctive Er:YAG laser in non‐surgical periodontal therapy of patients with inadequately controlled type 2 diabetes mellitus: A split‐mouth randomized controlled study. J of Periodontal Research. 2022;57:63–74.

433. Nazzal SQ, Al‐Dubai M, Mounir R, Ali S, Mounir M. Maxillary vertical alveolar ridge augmentation using computer‐guided sandwich osteotomy technique with simultaneous implant placement versus conventional technique: A pilot study. Clin Implant Dent Rel Res. 2021;23:842–50.

434. Starch‐Jensen T, Bruun NH. Patient’s perception of recovery after sinus membrane elevation and blood coagulum compared with 1:1 mixture of autogenous bone graft and deproteinized porcine bone mineral. Secondary outcomes from a single‐blinded randomized controlled trial. Clinical Oral Implants Res. 2022;33:65–77.

435. Cosyn J, Eeckhout C, Christiaens V, Eghbali A, Vervaeke S, Younes F, et al. A multi‐centre randomized controlled trial comparing connective tissue graft with collagen matrix to increase soft tissue thickness at the buccal aspect of single implants: 3‐month results. J Clinic Periodontology. 2021;48:1502–15.

436. Ahamed AS, Prakash PSG, Crena J, Victor DJ, Subramanian S, Appukuttan D. The influence of laser-microgrooved implant and abutment surfaces on mean crestal bone levels and peri-implant soft tissue healing: a 3-year longitudinal randomized controlled clinical trial. Int J Implant Dent. 2021;7:102.

437. Santamaria MP, Rossato A, Miguel MMV, Fonseca MB, Bautista CRG, de Marco AC, et al. Comparison of two types of xenogeneic matrices to treat single gingival recessions: A randomized clinical trial. Journal of Periodontology. 2022;93:709–20.

438. Starch-Jensen T, Ahmad M, Bruun NH, Becktor JP. Patient’s perception of recovery after maxillary sinus floor augmentation with autogenous bone graft compared with composite grafts: a single-blinded randomized controlled trial. Int J Implant Dent. 2021;7:99.

439. Cucchi A, Vignudelli E, Franceschi D, Randellini E, Lizio G, Fiorino A, et al. Vertical and horizontal ridge augmentation using customized CAD/CAM titanium mesh with versus without resorbable membranes. A randomized clinical trial. Clinical Oral Implants Res. 2021;32:1411–24.

440. Bielemann AM, Schuster AJ, Possebon AP da R, Schinestsck AR, Chagas‐Junior OL, Faot F. Clinical performance of narrow‐diameter implants with hydrophobic and hydrophilic surfaces with mandibular implant overdentures: 1‐year results of a randomized clinical trial. Clinical Oral Implants Res. 2022;33:21–32.

441. Wolfart S, Rittich A, Groß K, Hartkamp O, Stück A, Raith S, et al. Cemented versus screw‐retained posterior implant‐supported single crowns: A 24‐month randomized controlled clinical trial. Clinical Oral Implants Res. 2021;32:1484–95.

442. Barootchi S, Tavelli L, Di Gianfilippo R, Stefanini M, Zucchelli G, Rasperini G, et al. Gingival Phenotype Modification as a Result of Root Coverage Procedure with Two Human Dermal Matrices: Long-Term Assessment of a Randomized Clinical Trial. Int J Periodontics Restorative Dent. 2021;41:719–26.

443. Cardaropoli D, Albano M, Tamagnone L. Infection Control in Adult Periodontal Patients Using Ultrasonic Debridement and Erythritol Powder: A Randomized, Controlled, Split-Mouth Clinical Study. Int J Periodontics Restorative Dent. 2021;41:675–81.

444. Miguel MMV, Mathias‐Santamaria IF, Rossato A, Ferraz LFF, Rangel TP, Casarin RCV, et al. Enamel matrix derivative effects on palatal mucosa wound healing: Randomized clinical trial. J of Periodontal Research. 2021;56:1213–22.

445. Swami RK, Kolte AP, Kolte RA. Clinico‐radiographic comparative evaluation of 1% metformin gel plus platelet‐rich fibrin over platelet‐rich fibrin alone in the treatment of Grade II furcation defects: A randomized controlled double‐blind clinical trial. Journal of Periodontology. 2022;93:644–55.

446. Stein JM, Yekta‐Michael SS, Schittenhelm F, Reichert S, Kupietz D, Dommisch H, et al. Comparison of three full‐mouth concepts for the non‐surgical treatment of stage III and IV periodontitis: A randomized controlled trial. J Clinic Periodontology. 2021;48:1516–27.

447. Durand R, Kersheh I, Marcotte S, Boudrias P, Schmittbuhl M, Cresson T, et al. Do postoperative antibiotics influence one‐year peri‐implant crestal bone remodelling and morbidity? A double‐blinded randomized clinical trial. Clinical Oral Implants Res. 2021;32:1318–27.

448. Khouly I, Strauss FJ, Jung RE, Froum SJ. Effect of alveolar ridge preservation on clinical attachment level at adjacent teeth: A randomized clinical trial. Clin Implant Dent Relat Res. 2021;23:716–25.

449. Thoma DS, Wolleb K, Schellenberg R, Strauss F, Hämmerle CHF, Jung RE. Two short implants versus one short implant with a cantilever: 5‐Year results of a randomized clinical trial. J Clinic Periodontology. 2021;48:1480–90.

450. Ko K, Song YW, Park J, Park Y, Kim C, Lee J. Immediate loading protocols increase the risk of failure of implants placed by fully guided surgery in partially edentulous jaws: A randomized clinical trial. Clin Implant Dent Relat Res. 2021;23:735–44.

451. Fernandes D, Nunes S, López‐Castro G, Marques T, Montero J, Borges T. Effect of customized healing abutments on the peri‐implant linear and volumetric tissue changes at maxillary immediate implant sites: A 1‐year prospective randomized clinical trial. Clin Implant Dent Relat Res. 2021;23:745–57.

452. Dortaj D, Bassir SH, Hakimiha N, Hong H, Aslroosta H, Fekrazad R, et al. Efficacy of Nd:YAG laser‐assisted periodontal therapy for the management of periodontitis: A double‐blind split‐mouth randomized controlled clinical trial. Journal of Periodontology. 2022;93:662–72.

453. Barbosa P, Cruvinel T, Sakakura C, Lopes de Oliveira G, Zuza E. Primary and Secondary Stability of Implants with Hydrophilic Surfaces in the Posterior Maxilla: A Split-Mouth Randomized Controlled Clinical Trial. Int J Oral Maxillofac Implants. 2021;36:787–92.

454. Seleem A, Tawfik O, El-Nahass H. Evaluation of Oversized Drilling on Implant Survival and Stability Versus Traditional Drilling Technique: A Randomized Clinical Trial. Int J Oral Maxillofac Implants. 2021;36:771–8.

455. Frizzera F, Calazans N, Pascoal C, Martins M, Mendonça G. Flapless Guided Implant Surgeries Compared with Conventional Surgeries Performed by Nonexperienced Individuals: Randomized and Controlled Split-Mouth Clinical Trial. Int J Oral Maxillofac Implants. 2021;36:755–61.

456. Lv X, Qian S, Qiao S, Gu Y, Lai H, Shi J. Clinical, radiographic, and immunological evaluation of angulated screw‐retained and cemented single‐implant crowns in the esthetic region: A 1‐year randomized controlled clinical trial. Clin Implant Dent Relat Res. 2021;23:692–702.

457. Zafar F, Romano F, Citterio F, Ferrarotti F, Dellavia C, Chang M, et al. Chemical cleansing as an adjunct to subgingival instrumentation with ultrasonic and hand devices in deep periodontal pockets: a randomized controlled study. J Periodontal Implant Sci. 2021;51:276.

458. Asbi T, Hussein HA, Horwitz J, Gabay E, Machtei EE, Giladi HZ. A single application of chlorhexidine gel reduces gingival inflammation and interleukin 1‐β following one‐stage implant placement: A randomized controlled study. Clin Implant Dent Relat Res. 2021;23:726–34.

459. Doornewaard R, Sakani S, Matthys C, Glibert M, Bronkhorst E, Vandeweghe S, et al. Four‐implant‐supported overdenture treatment in the maxilla. Part I: A randomized controlled split mouth trial assessing the effect of microthreads and abutment connection type on 4 years peri‐implant health. Clin Implant Dent Relat Res. 2021;23:671–9.

460. Kim Y, Song YW, Kim MJ, Cha J, Park J, Kim J, et al. Immediate loading of fixed partial prostheses reconstructed using either tapered or straight implants in the posterior area: A randomized clinical trial. Clin Implant Dent Relat Res. 2021;23:703–15.

461. Lu H, He L, Jin D, Zhu Y, Meng H. Effect of adjunctive systemic antibiotics on microbial populations compared with scaling and root planing alone for the treatment of periodontitis: A pilot randomized clinical trial. Journal of Periodontology. 2022;93:570–83.

462. Enkling N, Nauli J, Kraus D, Wittneben JG, Schimmel M, Abou‐Ayash S. Short strategic implants for mandibular removable partial dentures: One‐year results from a pilot randomized crossover abutment type study. Clinical Oral Implants Res. 2021;32:1176–89.

463. Strauss G, Goteiner D, Murawski K, Singer S, Drew H, Sullivan A. Laser-Assisted Therapy for the Treatment of Peri-implantitis. Part I. Clinical Outcomes. Int J Periodontics Restorative Dent. 2021;41:563–8.

464. Tokuc B, Kan B. The effect of triangular cross‐section neck design on crestal bone stability in the anterior mandible: A randomized, controlled, split‐mouth clinical trial. Clinical Oral Implants Res. 2021;32:1241–50.

465. Cardaropoli D, Tamagnone L, Roffredo A, Gaveglio L. Influence of Abutment Design and Platform Switching on Peri-implant Marginal Bone Level: A Randomized Controlled Clinical Trial with 1-Year Results. Int J Periodontics Restorative Dent. 2021;41:547–53.

466. Jepsen K, Tietmann C, Kutschera E, Wüllenweber P, Jäger A, Cardaropoli D, et al. The effect of timing of orthodontic therapy on the outcomes of regenerative periodontal surgery in patients with stage IV periodontitis: A multicenter randomized trial. J Clin Periodontol. 2021;48:1282–92.

467. Mathias‐Santamaria IF, Silveira CA, Rossato A, Sampaio de Melo MA, Bresciani E, Santamaria MP. Single gingival recession associated with non‐carious cervical lesion treated by partial restoration and coronally advanced flap with or without xenogenous collagen matrix: A randomized clinical trial evaluating the coverage procedures and restorative protocol. Journal of Periodontology. 2022;93:504–14.

468. McGuire MK, Janakievski J, Scheyer ET, Velásquez D, Gunsolley JC, Heard RH, et al. Efficacy of a harvest graft substitute for recession coverage and soft tissue volume augmentation: A randomized controlled trial. Journal of Periodontology. 2022;93:333–42.

469. Gurpegui Abud D, Shariff JA, Linden E, Kang PY. Erbium‐doped: yttrium‐aluminum‐garnet (Er:YAG) versus scaling and root planing for the treatment of periodontal disease: A single‐blinded split‐mouth randomized clinical trial. Journal of Periodontology. 2022;93:493–503.

470. Nielsen HB, Schou S, Bruun NH, Starch-Jensen T. Single-crown restorations supported by short implants (6 mm) compared with standard-length implants (13 mm) in conjunction with maxillary sinus floor augmentation: a randomized, controlled clinical trial. Int J Implant Dent. 2021;7:66.

471. Engkawong S, Mattheos N, Pisarnturakit PP, Pimkhaokham A, Subbalekha K. Comparing p atient‐reported outcomes and experiences among static, dynamic c omputer‐aided , and conventional freehand dental implant placement: A randomized clinical trial. Clin Implant Dent Relat Res. 2021;23:660–70.

472. Pera F, Menini M, Bagnasco F, Mussano F, Ambrogio G, Pesce P. Evaluation of internal and external hexagon connections in immediately loaded full‐arch rehabilitations: A within‐person randomized split‐mouth controlled trial with a 3‐year follow‐up. Clin Implant Dent Rel Res. 2021;23:562–7.

473. Huang J, Liu J, Wu Y, Dai A, Hu H, He F, et al. Clinical evaluation of xenogeneic collagen matrix versus free gingival grafts for keratinized mucosa augmentation around dental implants: A randomized controlled clinical trial. J Clin Periodontol. 2021;48:1293–301.

474. Garcia‐Sanchez R, Mardas N, Buti J, Ortiz Ruiz AJ, Pardo Zamora G. Immediate implant placement in fresh alveolar sockets with a minimal split‐thickness envelope flap: A randomised controlled clinical trial. Clinical Oral Implants Res. 2021;32:1115–26.

475. Canullo L, Masucci L, Quaranta G, Patini R, Caponio VCA, Pesce P, et al. Culturomic and quantitative real‐time ‐ polymerase chain reaction analyses for early contamination of abutments with different surfaces: A randomized clinical trial. Clin Implant Dent Rel Res. 2021;23:568–78.

476. Katheng A, Kanazawa M, Komagamine Y, Miyayasu A, Uehara Y, Sato D, et al. Masticatory performances and maximum occlusal forces of immediate and conventional loaded two-implant supported overdentures retained by magnetic attachments: preliminary study of randomized controlled clinical trial. Int J Implant Dent. 2021;7:57.

477. Renvert S, Giovannoli J, Roos‐Jansåker A, Rinke S. Surgical treatment of peri‐implantitis with or without a deproteinized bovine bone mineral and a native bilayer collagen membrane: A randomized clinical trial. J Clin Periodontol. 2021;48:1312–21.

478. Søndergaard K, Hosseini M, Storgård Jensen S, Spin‐Neto R, Gotfredsen K. Fully versus conventionally guided implant placement by dental students: A randomized controlled trial. Clinical Oral Implants Res. 2021;32:1072–84.

479. Kappel S, Klotz A, Eberhard L, Lorenzo Bermejo J, Rammelsberg P, Giannakopoulos NN. Maxillary implant overdentures on two or four implants. A prospective randomized cross‐over clinical trial of implant and denture success and survival. Clinical Oral Implants Res. 2021;32:1061–71.

480. Ma F, Lin Y, Sun F, Jiang X, Wei T. The impact of autologous concentrated growth factors on the alveolar ridge preservation after posterior tooth extraction: A prospective, randomized controlled clinical trial. Clin Implant Dent Rel Res. 2021;23:579–92.

481. Park S, Song YW, Cha J, Lee J, Kim Y, Shin H, et al. Adjunctive use of metronidazole‐minocycline ointment in the nonsurgical treatment of peri‐implantitis: A multicenter randomized controlled trial. Clin Implant Dent Rel Res. 2021;23:543–54.

482. Rubino CV, Katz BG, Langlois K, Wang HH, Carrion JA, Walker SG, et al. Evaluation of different materials used for sealing of implant abutment access channel and the peri‐implant sulcus microbiota: A 6‐month, randomized controlled trial. Clinical Oral Implants Res. 2021;32:941–50.

483. Jonker BP, Strauss FJ, Naenni N, Jung RE, Wolvius EB, Pijpe J. Early implant placement with or without alveolar ridge preservation in single tooth gaps renders similar esthetic, clinical and patient‐reported outcome measures: One‐year results of a randomized clinical trial. Clinical Oral Implants Res. 2021;32:1041–51.

484. Kumar V, Singhal R, Rastogi P, Lal N, Pandey S, Mahdi AA. Localized probiotic-guided pocket recolonization in the treatment of chronic periodontitis: a randomized controlled clinical trial. J Periodontal Implant Sci. 2021;51:199.

485. Tresguerres FGF, Tresguerres IF, Iglesias O, Leco I, Tamimi F, Torres J. The role of cortical perforations in allogeneic block grafting for lateral augmentation in maxilla: A randomized clinical trial. Clin Implant Dent Rel Res. 2021;23:530–42.

486. Hamzah B, Mounir R, Ali S, Mounir M. Maxillary horizontal alveolar ridge augmentation using computer guided ridge splitting with simultaneous implant placement versus conventional technique: A randomized clinical trial. Clin Implant Dent Rel Res. 2021;23:555–61.

487. Magdy M, Abdelkader MA, Alloush S, Fawzy El‐Sayed KM, Nawwar AA, Shoeib M, et al. Ultra‐short versus standard‐length dental implants in conjunction with osteotome‐mediated sinus floor elevation: A randomized controlled clinical trial. Clin Implant Dent Rel Res. 2021;23:520–9.

488. Muñoz M, Busoms E, Vilarrasa J, Albertini M, Ruíz‐Magaz V, Nart J. Bone‐level changes around implants with 1‐ or 3‐mm‐high abutments and their relation to crestal mucosal thickness: A 1‐year randomized clinical trial. J Clin Periodontol. 2021;48:1302–11.

489. Derksen W, Tahmaseb A, Wismeijer D. Randomized Clinical Trial comparing clinical adjustment times of CAD/CAM screw‐retained posterior crowns on ti‐base abutments created with digital or conventional impressions. One‐year follow‐up. Clinical Oral Implants Res. 2021;32:962–70.

490. Sghaireen M, Ganji K, Alam M, Rahman S, Billah S. Mineralized Plasmatic Matrix in Ridge Preservation: A Randomized Controlled Clinical Trial. Int J Periodontics Restorative Dent. 2021;41:e103–12.

491. Ruiz Henao PA, Caneiro Queija L, Mareque S, Tasende Pereira A, Liñares González A, Blanco Carrión J. Titanium vs ceramic single dental implants in the anterior maxilla: A 12‐month randomized clinical trial. Clinical Oral Implants Res. 2021;32:clr.13788.

492. Ongphichetmetha N, Lertpimonchai A, Champaiboon C. Bioactive glass and arginine dentifrices immediately relieved dentine hypersensitivity following non‐surgical periodontal therapy: A randomized controlled trial. Journal of Periodontology. 2022;93:248–57.

493. Kinalski M de A, Agostini BA, Bergoli CD, dos Santos MBF. Influence of low-level laser therapy on implant stability in implants placed in healed sites: a randomized controlled trial. Int J Implant Dent. 2021;7:49.

494. Wortmann DE, Klein‐Nulend J, Ruijven LJ, Schortinghuis J, Vissink A, Raghoebar GM. Incorporation of anterior iliac crest or calvarial bone grafts in reconstructed atrophied maxillae: A randomized clinical trial with histomorphometric and micro‐CT analyses. Clin Implant Dent Relat Res. 2021;23:492–502.

495. Lee J, Jeong S. Long‐term stability of adjunctive use of enamel matrix protein derivative on porcine‐derived xenograft for the treatment of one‐wall intrabony defects: A 4‐year extended follow‐up of a randomized controlled trial. Journal of Periodontology. 2022;93:231–8.

496. Gunpinar S, Meraci B. Periodontal health education session can improve oral hygiene in patients with gingivitis: A masked randomized controlled clinical study. Journal of Periodontology. 2022;93:220–30.

497. Santos BFE, Costa FO, Vasconcelos AMA, Cyrino RM, Cota LOM. Preemptive effects of ibuprofen and nimesulide on postoperative pain control after open flap periodontal surgeries: A randomized placebo‐controlled split‐mouth clinical trial. Journal of Periodontology. 2022;93:300–9.

498. Atef M, El Barbary A, Dahrous MSE, Zahran AF. Comparison of the soft and hard peri‐implant tissue dimensional changes around single immediate implants in the esthetic zone with socket shield technique versus using xenograft: A randomized controlled clinical trial. Clin Implant Dent Relat Res. 2021;23:456–65.

499. Oliveira AM, Lourenço TGB, Colombo APV. Impact of systemic probiotics as adjuncts to subgingival instrumentation on the oral‐gut microbiota associated with periodontitis: A randomized controlled clinical trial. Journal of Periodontology. 2022;93:31–44.

500. Vieth MP, Deas DE, Archontia Palaiologou A, Diogenes A, Mader MJ, Mealey BL. Effect of intravenous dexamethasone on postoperative pain and swelling following periodontal flap surgery: A randomized controlled trial of patient‐centered outcomes. Journal of Periodontology. 2022;93:239–47.

501. Santos A, Botelho J, Machado V, Borrecho G, Proença L, Mendes JJ, et al. Autogenous Mineralized Dentin versus Xenograft granules in Ridge Preservation for Delayed Implantation in Post‐extraction Sites: A Randomized controlled clinical trial with an 18 months follow‐up. Clinical Oral Implants Res. 2021;32:905–15.

502. Choi B, Lee YC, Oh KC, Lee JH. Effects of photofunctionalization on early osseointegration of titanium dental implants in the maxillary posterior region: a randomized double-blinded clinical trial. Int J Implant Dent. 2021;7:37.

503. Monje A, Pérez A, Vera‐Rodriguez M, Nart J, Catena A, Petrova D. Comprehension and recall of information about factors associated with peri‐implantitis: A randomized controlled trial. Journal of Periodontology. 2022;93:89–99.

504. Vincent-Bugnas S, Laurent J, Naman E, Charbit M, Borie G. Treatment of multiple gingival recessions with xenogeneic acellular dermal matrix compared to connective tissue graft: a randomized split-mouth clinical trial. J Periodontal Implant Sci. 2021;51:77.

505. Naicker M, Ngo LH, Rosenberg AJ, Darby IB. The effectiveness of using the perioscope as an adjunct to non‐surgical periodontal therapy: Clinical and radiographic results. Journal of Periodontology. 2022;93:20–30.

506. Tirone F, Salzano S, Rodi D, Pozzatti L. Three-Year Evaluation of the Influence of Implant Surfaces on Implant Failure and Peri-implantitis: A Double-Blind Randomized Controlled Trial with Split-Mouth Design. Int J Oral Maxillofac Implants. 2021;36:e23–30.

507. Elsyad M, Alsabri M, Elgamal M, Al-Tonbary G. Resilient Stud Versus Bar Attachments for Immediately Loaded Implants Supporting Mandibular Overdentures: 1-year Randomized Controlled Clinical Trial. Int J Oral Maxillofac Implants. 2021;36:346–54.

508. de Resende GP, Dias AP, Leles JLR, de Souza JAC, Leles CR. Postsurgical oral symptoms after insertion of one or two implants for mandibular overdentures: short-term results of a randomized clinical trial. Int J Implant Dent. 2021;7:38.

509. González‐Serrano J, López‐Pintor RM, Serrano J, Torres J, Hernández G, Sanz M. Short‐term efficacy of a gel containing propolis extract, nanovitamin C and nanovitamin E on peri‐implant mucositis: A double‐blind, randomized, clinical trial. J Periodont Res. 2021;56:897–906.

510. Mendez M, Angst PDM, Oppermann RV, Velden U, Gomes SC. Oral health‐related quality of life during supportive periodontal therapy: results from a randomized clinical trial. J Clin Periodontol. 2021;48:1103–10.

511. Apatzidou DA, Bakopoulou AA, Kouzi‐Koliakou K, Karagiannis V, Konstantinidis A. A tissue‐engineered biocomplex for periodontal reconstruction. A proof‐of‐principle randomized clinical study. J Clin Periodontol. 2021;48:1111–25.

512. Garoushi IH, Elbeialy RR, Gibaly A, Atef M. Evaluation of the effect of the lateralized inferior alveolar nerve isolation and bone grafting on the nerve function and implant stability. (Randomized Clinical Trial). Clin Implant Dent Relat Res. 2021;23:423–31.

513. Teles FRF, Lynch MC, Patel M, Torresyap G, Martin L. Bacterial resistance to minocycline after adjunctive minocycline microspheres during periodontal maintenance: A randomized clinical trial. J Periodontol. 2021;92:1222–31.

514. Bozkaya S, Uraz A, Guler B, Kahraman SA, Turhan Bal B. The stability of implants and microbiological effects following photobiomodulation therapy with one‐stage placement: A randomized, controlled, single‐blinded, and split‐mouth clinical study. Clin Implant Dent Relat Res. 2021;23:329–40.

515. Zuhr O, Akakpo D, Eickholz P, Vach K, Hürzeler MB, Petsos H, et al. Tunnel technique with connective tissue graft versus coronally advanced flap with enamel matrix derivate for root coverage: 5‐year results of an RCT using 3D digital measurement technology for volumetric comparison of soft tissue changes. J Clin Periodontol. 2021;48:949–61.

516. Castro AB, Van Dessel J, Temmerman A, Jacobs R, Quirynen M. Effect of different platelet‐rich fibrin matrices for ridge preservation in multiple tooth extractions: A split‐mouth randomized controlled clinical trial. J Clin Periodontol. 2021;48:984–95.

517. Hentenaar DFM, De Waal YCM, Stewart RE, Van Winkelhoff AJ, Meijer HJA, Raghoebar GM. Erythritol airpolishing in the non‐surgical treatment of peri‐implantitis: A randomized controlled trial. Clin Oral Impl Res. 2021;32:840–52.

518. Ragghianti-Zangrando M, Brondino N, Damante C, de Fátima Balderrama Í, Veronesi G, Cardoso M, et al. Clinical Outcomes and Gingival Blood Flowmetry of Two Types of Subepithelial Connective Tissue Graft for Root Coverage in Multiple Gingival Recessions: A Preliminary Study. Int J Periodontics Restorative Dent. 2021;41:285–93.

519. Koutouzis T, Ali A. The Influence of Abutment Macrodesign on Facial Peri-implant Tissue Dimensions for Guided Placed and Restored Implants at Healed Sites: 1-Year CBCT Findings from a Randomized Controlled Clinical Trial. Int J Periodontics Restorative Dent. 2021;41:277–83.

520. Kadkhodazadeh M, Amid R, Shirvan H, Namdari M. Clinical Efficacy of Gingival Thickening Following the Use of Allogeneic Acellular and Xenogeneic Collagen Matrix: A 12-Month Randomized Clinical Trial. Int J Periodontics Restorative Dent. 2021;41:e63–71.

521. Tonetti MS, Cortellini P, Bonaccini D, Deng K, Cairo F, Allegri M, et al. Autologous connective tissue graft or xenogenic collagen matrix with coronally advanced flaps for coverage of multiple adjacent gingival recession. 36‐month follow‐up of a randomized multicentre trial. J Clin Periodontol. 2021;48:962–9.

522. Preshaw PM, Ide M, Bissett SM, Holliday R, Lansdowne N, Pickering K, et al. No benefit of an adjunctive phototherapy protocol in treatment of periodontitis: A split‐mouth randomized controlled trial. J Clin Periodontol. 2021;48:1093–102.

523. Sonnenschein SK, Ziegler P, Ciardo A, Ruetters M, Krisam J, Kim T. The impact of splinting mobile mandibular incisors on Oral Health‐Related Quality of Life—Preliminary observations from a randomized clinical trial. J Clin Periodontol. 2021;48:816–25.

524. Bakhishov H, Isler SC, Bozyel B, Yıldırım B, Tekindal MA, Ozdemir B. De‐epithelialized gingival graft versus subepithelial connective tissue graft in the treatment of multiple adjacent gingival recessions using the tunnel technique: 1‐year results of a randomized clinical trial. J Clin Periodontol. 2021;48:970–83.

525. Arghami A, Simmons D, St. Germain J, Maney P. Immediate and early loading of hydrothermally treated, hydroxyapatite-coated dental implants: a 7-year prospective randomized clinical study. Int J Implant Dent. 2021;7:21.

526. Starch-Jensen T, Bruun NH. Patient’s perception of recovery after osteotome-mediated sinus floor elevation with Bio-Oss collagen compared with no grafting material: a randomized single-blinded controlled trial. Int J Implant Dent. 2021;7:20.

527. Slagter KW, Meijer HJA, Hentenaar DFM, Vissink A, Raghoebar GM. Immediate single‐tooth implant placement with simultaneous bone augmentation versus delayed implant placement after alveolar ridge preservation in bony defect sites in the esthetic region: A 5‐year randomized controlled trial. Journal of Periodontology. 2021;92:1738–48.

528. Mercado F, Vaquette C, Hamlet S, Ivanovski S. Enamel matrix derivative promotes new bone formation in xenograft assisted maxillary anterior ridge preservation—A randomized controlled clinical trial. Clin Oral Impl Res. 2021;32:732–44.

529. Cruz R, Moraschini V, Calasans‐Maia MD, Almeida DCF, Sartoretto SC, Granjeiro JM. Clinical efficacy of simvastatin gel combined with polypropylene membrane on the healing of extraction sockets: A triple‐blind, randomized clinical trial. Clin Oral Impl Res. 2021;32:711–20.

530. Križaj Dumić A, Pajk F, Olivi G. The effect of post‐extraction socket preservation laser treatment on bone density 4 months after extraction: Randomized controlled trial. Clin Implant Dent Relat Res. 2021;23:309–16.

531. Ferrantino L, Camurati A, Gambino P, Marzolo M, Trisciuoglio D, Santoro G, et al. Aesthetic outcomes of non‐functional immediately restored single post‐extraction implants with and without connective tissue graft: A multicentre randomized controlled trial. Clin Oral Impl Res. 2021;32:684–94.

532. Sánchez-Fernández E, Magán-Fernández A, O’Valle F, Bravo M, Mesa F. Hyaluronic acid reduces inflammation and crevicular fluid IL-1β concentrations in peri-implantitis: a randomized controlled clinical trial. J Periodontal Implant Sci. 2021;51:63.

533. Aldahouk A, Elbeialy RR, Gibaly A, Shawky M, Atef M. The assessment of the effect of the size of lateral‐antrostomy in graftless balloon elevation of the maxillary sinus membrane with simultaneous implant placement (a randomized controlled clinical trial). Clin Implant Dent Relat Res. 2021;23:31–42.

534. Hasturk H, Steed D, Tosun E, Martins M, Floros C, Nguyen D, et al. Use of amnion‐derived cellular cytokine solution for the treatment of gingivitis: A 2‐week safety, dose‐ranging, proof‐of‐principle randomized trial. J Periodontol. 2021;92:1317–28.

535. Shi J, Lai Y, Qian S, Qiao S, Tonetti MS, Lai H. Clinical, radiographic and economic evaluation of short‐6‐mm implants and longer implants combined with osteotome sinus floor elevation in moderately atrophic maxillae: A 3‐year randomized clinical trial. J Clin Periodontol. 2021;48:695–704.

536. Liu K, Huang Z, Chen Z, Han B, Ouyang X. Treatment of periodontal intrabony defects using bovine porous bone mineral and guided tissue regeneration with/without platelet‐rich fibrin: A randomized controlled clinical trial. Journal of Periodontology. 2021;92:1546–53.

537. Hauck KE, Trentin MS, Skiba THI, Shibli JA, De Carli JP. Clinical and satisfaction outcomes of using one or two dental implants for mandibular overdentures: preliminary short-term follow-up of a randomized clinical trial. Int J Implant Dent. 2021;7:10.

538. Elfana A, El‐Kholy S, Saleh HA, Fawzy El‐Sayed K. Alveolar ridge preservation using autogenous whole‐tooth versus demineralized dentin grafts: A randomized controlled clinical trial. Clin Oral Impl Res. 2021;32:539–48.

539. Hartlev J, Schou S, Isidor F, Nørholt SE. A clinical and radiographic study of implants placed in autogenous bone grafts covered by either a platelet-rich fibrin membrane or deproteinised bovine bone mineral and a collagen membrane: a pilot randomised controlled clinical trial with a 2-year follow-up. Int J Implant Dent. 2021;7:8.

540. Rexhepi I, Paolantonio M, Romano L, Serroni M, Santamaria P, Secondi L, et al. Efficacy of inorganic bovine bone combined with leukocyte and platelet‐rich fibrin or collagen membranes for treating unfavorable periodontal infrabony defects: Randomized non‐inferiority trial. Journal of Periodontology. 2021;92:1576–87.

541. Kulkarni MR, Shettar LG, Bakshi PV, Nikhil K. Palatal pre‐suturing for perioperative hemostasis at free gingival graft donor sites: A randomized, controlled clinical trial. Journal of Periodontology. 2021;92:1441–7.

542. Jentsch HFR, Roccuzzo M, Pilloni A, Kasaj A, Fimmers R, Jepsen S. Flapless application of enamel matrix derivative in periodontal retreatment: A multicentre randomized feasibility trial. J Clin Periodontol. 2021;48:659–67.

543. Lou F, Rao P, Zhang M, Luo S, Lu S, Xiao J. Accuracy evaluation of partially guided and fully guided templates applied to implant surgery of anterior teeth: A randomized controlled trial. Clin Implant Dent Relat Res. 2021;23:117–30.

544. Linkevicius T, Linkevicius R, Gineviciute E, Alkimavicius J, Mazeikiene A, Linkeviciene L. The influence of new immediate tissue level abutment on crestal bone stability of subcrestally placed implants: A 1‐year randomized controlled clinical trial. Clin Implant Dent Relat Res. 2021;23:259–69.
