# Supplementary material for: Reporting of flow diagrams in randomised controlled trials published in periodontology and implantology: a survey
Source: BMC Med Res Methodol. 2023 Apr 27;23:105. doi: 10.1186/s12874-023-01923-7 (PMC10134555; doi:10.1186/s12874-023-01923-7)
Supplement: Supplementary file 4 — Additional file 4. Articles excluded after abstract assessment with reasons. [file 12874_2023_1923_MOESM4_ESM.docx]

| Article  **Additional file 4, Articles excluded after abstract assessment with reasons** | Reason for exclusion |
| --- | --- |
| 1. Fang Y, Bi Y, Mashrah M, Su Y, Ge L, Dong Y, et al. Does sinus floor elevation in the presence of Schneiderian membrane pathology increase therisk of membrane perforation and implant failure rate? J Oral Implantol. 2020. https://doi.org/10.1563/aaid-joi-D-20-00145.  2. Anitua E, Fernandez-de-Retana S, Anitua B, Alkhraisat MH. Long-Term Retrospective Study of 3.0-mm-Diameter Implants Supporting Fixed Multiple Prostheses: Immediate Versus Delayed Implant Loading. Int J Oral Maxillofac Implants. 2020;35:1229–38.  3. Gargallo-Albiol J, Barootchi S, Marqués-Guasch J, Wang H-L. Fully Guided Versus Half-Guided and Freehand Implant Placement: Systematic Review and Meta-analysis. Int J Oral Maxillofac Implants. 2020;35:1159–69.  4. Oh W-S, Oh J, Valcanaia AJ. Open Proximal Contact with Implant-Supported Fixed Prostheses Compared with Tooth-Supported Fixed Prostheses: A Systematic Review and Meta-analysis. Int J Oral Maxillofac Implants. 2020;30:e99–108.  5. Ye M, Liu W, Cheng S, Yan L. Immediate vs conventional loading of mandibular overdentures: A comprehensive systematic review and meta-analysis of randomized controlled trials. J Oral Implantol. 2020. https://doi.org/10.1563/aaid-joi-D-20-00265.  6. Pistilli R, Zucchelli G, Barausse C, Bonifazi L, Karaban M, Gasparro R, et al. Minimally Invasive Fixed Rehabilitation of an Extremely Atrophic Posterior Mandible Using 4-mm Ultrashort Implants: A Case Report with a 7-Year Follow-up. Int J Periodontics Restorative Dent. 2020;40:e235–40.  7. Mohajerani H, Irajian G, Latifi F, Masjedian F, Tabrizi R. Efficacy of low-dose local clindamycin in different times for microbial decontamination of autogenous particulate bone graft. Int J Implant Dent. 2020;6:70.  8. Dilber E, Hagenfeld D, Ehmke B, Faggion CM. A systematic review on bacterial community changes after periodontal therapy with and without systemic antibiotics: An analysis with a wider lens. J Periodontal Res. 2020;55:785–800.  9. Siqueira R, Chen Z, Galli M, Saleh I, Wang H-L, Chan H-L. Does a fully digital workflow improve the accuracy of computer-assisted implant surgery in partially edentulous patients? A systematic review of clinical trials. Clin Implant Dent Relat Res. 2020;22:660–71.  10. Sánchez N, Fierravanti L, Núñez J, Vignoletti F, González-Zamora M, Santamaría S, et al. Periodontal regeneration using a xenogeneic bone substitute seeded with autologous periodontal ligament-derived mesenchymal stem cells: A 12-month quasi-randomized controlled pilot clinical trial. J Clin Periodontol. 2020;47:1391–402.  11. Wessels R, Vervaeke S, Seyssens L, Eghbali A, Cosyn J. A 5-year cohort study on early implant placement with guided bone regeneration or alveolar ridge preservation with connective tissue graft. Clin Implant Dent Relat Res. 2020;22:697–705.  12. Iezzi G, Perrotti V, Felice P, Barausse C, Piattelli A, Del Fabbro M. Are <7-mm long implants in native bone as effective as longer implants in augmented bone for the rehabilitation of posterior atrophic jaws? A systematic review and meta-analysis. Clin Implant Dent Relat Res. 2020;22:552–66.  13. Blaschke C, Schwass DR. The socket-shield technique: a critical literature review. Int J Implant Dent. 2020;6:52.  14. Deeb JG, Frantar A, Deeb GR, Carrico CK, Rener-Sitar K. In Vitro Comparison of Time and Accuracy of Implant Placement Using Trephine and Conventional Drilling Techniques Under Dynamic Navigation. J Oral Implantol. 2020. https://doi.org/10.1563/aaid-joi-D-19-00125.  15. Wu X, Hu Q, Yan Q, Zhang T, Riley P, Hua F, et al. Trends in the level of evidence and impact of clinical studies published in leading oral implantology journals: 2008-2018. Clin Oral Implants Res. 2020;31:980–91.  16. Mongardini C, Zeza B, Pelagalli P, Blasone R, Scilla M, Berardini M. Radiographic bone level around particular laser-treated dental implants: 1 to 6 years multicenter retrospective study. Int J Implant Dent. 2020;6:29.  17. Tavelli L, Barootchi S, Avila-Ortiz G, Urban IA, Giannobile WV, Wang H-L. Peri-implant soft tissue phenotype modification and its impact on peri-implant health: A systematic review and network meta-analysis. J Periodontol. 2021;92:21–44.  18. Bedeloğlu E, Yalçın M, Koyuncuoğlu CZ. Is Perioperative Antibiotic Necessary in Straightforward Implant Placement Procedures? J Oral Implantol. 2021;47:135–9.  19. Tattan M, Chambrone L, González-Martín O, Avila-Ortiz G. Static computer-aided, partially guided, and free-handed implant placement: A systematic review and meta-analysis of randomized controlled trials. Clin Oral Implants Res. 2020;31:889–916.  20. Cairo F, Barootchi S, Tavelli L, Barbato L, Wang H-L, Rasperini G, et al. Aesthetic-And patient-related outcomes following root coverage procedures: A systematic review and network meta-analysis. J Clin Periodontol. 2020;47:1403–15.  21. Di Gianfilippo R, Valente NA, Toti P, Wang HL, Barone A. Influence of implant mucosal thickness on early bone loss: a systematic review with meta-analysis. J Periodontal Implant Sci. 2020;50:209–25.  22. Wu X, Yan Q, Fang X, Hua F, Shi B, Tu Y-K. Spin in the abstracts of randomized controlled trials in periodontology and oral implantology: A cross-sectional analysis. J Clin Periodontol. 2020;:1079–86.  23. Pelekos G, Acharya A, Eiji N, Hong G, Leung WK, McGrath C. Effects of adjunctive probiotic L. reuteri lozenges on S/RSD outcomes at molar sites with deep pockets. J Clin Periodontol. 2020;47:1098–107.  24. Saravi BE, Putz M, Patzelt S, Alkalak A, Uelkuemen S, Boeker M. Marginal bone loss around oral implants supporting fixed versus removable prostheses: a systematic review. Int J Implant Dent. 2020;6:20.  25. Monje A, Pons R, Roccuzzo A, Salvi GE, Nart J. Reconstructive therapy for the management of peri-implantitis via submerged guided bone regeneration: A prospective case series. Clin Implant Dent Relat Res. 2020;22:342–50.  26. Canullo L, Troiano G, Sbricoli L, Guazzo R, Laino L, Caiazzo A, et al. The Use of Antibiotics in Implant Therapy: A Systematic Review and Meta-Analysis with Trial Sequential Analysis on Early Implant Failure. Int J Oral Maxillofac Implants. 2020;35:485–94.  27. Sosakul T, Tuchpramuk P, Suvannapruk W, Srion A, Rungroungdouyboon B, Suwanprateeb J. Evaluation of tissue ingrowth and reaction of a porous polyethylene block as an onlay bone graft in rabbit posterior mandible. J Periodontal Implant Sci. 2020;50:106–20.  28. Barootchi S, Tavelli L, Zucchelli G, Giannobile WV, Wang H-L. Gingival phenotype modification therapies on natural teeth: A network meta-analysis. J Periodontol. 2020;91:1386–99.  29. Lee C-T, Sanz-Miralles E, Zhu L, Glick J, Heath A, Stoupel J. Predicting bone and soft tissue alterations of immediate implant sites in the esthetic zone using clinical parameters. Clin Implant Dent Relat Res. 2020;22:325–32.  30. Ponzoni D, Martins FEPB, Conforte JJ, Egas LS, Tonini KR, de Carvalho PSP. Evaluation of immediate cell viability and repair of osteotomies for implants using drills and piezosurgery. A randomized, prospective, and controlled rabbit study. Clin Implant Dent Relat Res. 2020;22:250–60.  31. Ackermann K-L, Barth T, Cacaci C, Kistler S, Schlee M, Stiller M. Clinical and patient-reported outcome of implant restorations with internal conical connection in daily dental practices: prospective observational multicenter trial with up to 7-year follow-up. Int J Implant Dent. 2020;6:14.  32. Rovai ES, Ambrosio LMB, Morillo CMR, Villar CC, Holzhausen M, Santamaria MP, et al. Root Coverage Procedures in Noncarious Cervical Lesions With and Without Restoration: A Systematic Review and Meta-Analysis. Int J Periodontics Restorative Dent. 2020;40:e127–35.  33. Jing W, Jiao J, Xu L, Hou J-X, Li X-T, Wang X-X, et al. Periodontal soft- and hard-tissue changes after augmented corticotomy in Chinese adult patients with skeletal Angle Class III malocclusion: A non-randomized controlled trial. J Periodontol. 2020;91:1419–28.  34. Ferrarotti F, Giraudi M, Citterio F, Fratini A, Gualini G, Piccoli GM, et al. Pocket elimination after osseous resective surgery: A systematic review and meta-analysis. J Clin Periodontol. 2020;47:756–67.  35. Trombelli L, Farina R, Pollard A, Claydon N, Franceschetti G, Khan I, et al. Efficacy of alternative or additional methods to professional mechanical plaque removal during supportive periodontal therapy: A systematic review and meta-analysis. J Clin Periodontol. 2020;47 Suppl 22:144–54.  36. Ye P, Wei T, Wang Y, Cai Y-J. Autologous Platelet Concentrates as Clinical Substitutes for Connective Tissue Graft in the Treatment of Miller Class I and II Gingival Recessions: An Updated Meta-Analysis. Int J Periodontics Restorative Dent. 2020;40:e53–63.  37. Teughels W, Feres M, Oud V, Martín C, Matesanz P, Herrera D. Adjunctive effect of systemic antimicrobials in periodontitis therapy: A systematic review and meta-analysis. J Clin Periodontol. 2020;47 Suppl 22:257–81.  38. Sanz-Sánchez I, Montero E, Citterio F, Romano F, Molina A, Aimetti M. Efficacy of access flap procedures compared to subgingival debridement in the treatment of periodontitis. A systematic review and meta-analysis. J Clin Periodontol. 2020;47 Suppl 22:282–302.  39. Cheng Q, Su Y-Y, Wang X, Chen S. Clinical Outcomes Following Immediate Loading of Single-Tooth Implants in the Esthetic Zone: A Systematic Review and Meta-Analysis. Int J Oral Maxillofac Implants. 2020;35:167–77.  40. Aizcorbe-Vicente J, Peñarrocha-Oltra D, Canullo L, Soto-Peñaloza D, Peñarrocha-Diago M. Influence of Facial Bone Thickness After Implant Placement into the Healed Ridges on the Remodeled Facial Bone and Considering Soft Tissue Recession: A Systematic Review. Int J Oral Maxillofac Implants. 2020;35:107–19.  41. Dommisch H, Walter C, Dannewitz B, Eickholz P. Resective surgery for the treatment of furcation involvement: A systematic review. J Clin Periodontol. 2020;47 Suppl 22:375–91.  42. Herrera D, Matesanz P, Martín C, Oud V, Feres M, Teughels W. Adjunctive effect of locally delivered antimicrobials in periodontitis therapy: A systematic review and meta-analysis. J Clin Periodontol. 2020;47 Suppl 22:239–56.  43. Carra MC, Detzen L, Kitzmann J, Woelber JP, Ramseier CA, Bouchard P. Promoting behavioural changes to improve oral hygiene in patients with periodontal diseases: A systematic review. J Clin Periodontol. 2020;47 Suppl 22:72–89.  44. Polak D, Wilensky A, Antonoglou GN, Shapira L, Goldstein M, Martin C. The efficacy of pocket elimination/reduction compared to access flap surgery: A systematic review and meta-analysis. J Clin Periodontol. 2020;47 Suppl 22:303–19.  45. Santiago JF, Lemos CAA, de Luna Gomes JM, Verri FR, Moraes SLD, Pellizzer EP. Quality Assessment of Systematic Reviews on Platform-Switching vs Platform-Matched Implants: An Overview. J Oral Implantol. 2020;46:153–62.  46. Suvan J, Leira Y, Moreno Sancho FM, Graziani F, Derks J, Tomasi C. Subgingival instrumentation for treatment of periodontitis. A systematic review. J Clin Periodontol. 2020;47 Suppl 22:155–75.  47. Figuero E, Roldán S, Serrano J, Escribano M, Martín C, Preshaw PM. Efficacy of adjunctive therapies in patients with gingival inflammation: A systematic review and meta-analysis. J Clin Periodontol. 2020;47 Suppl 22:125–43.  48. Nibali L, Koidou VP, Nieri M, Barbato L, Pagliaro U, Cairo F. Regenerative surgery versus access flap for the treatment of intra-bony periodontal defects: A systematic review and meta-analysis. J Clin Periodontol. 2020;47 Suppl 22:320–51.  49. Jepsen S, Gennai S, Hirschfeld J, Kalemaj Z, Buti J, Graziani F. Regenerative surgical treatment of furcation defects: A systematic review and Bayesian network meta-analysis of randomized clinical trials. J Clin Periodontol. 2020;47 Suppl 22:352–74.  50. Salvi GE, Stähli A, Schmidt JC, Ramseier CA, Sculean A, Walter C. Adjunctive laser or antimicrobial photodynamic therapy to non-surgical mechanical instrumentation in patients with untreated periodontitis: A systematic review and meta-analysis. J Clin Periodontol. 2020;47 Suppl 22:176–98.  51. Donos N, Calciolari E, Brusselaers N, Goldoni M, Bostanci N, Belibasakis GN. The adjunctive use of host modulators in non-surgical periodontal therapy. A systematic review of randomized, placebo-controlled clinical studies. J Clin Periodontol. 2020;47 Suppl 22:199–238.  52. Duttenhoefer F, Fuessinger MA, Beckmann Y, Schmelzeisen R, Groetz KA, Boeker M. Dental implants in immunocompromised patients: a systematic review and meta-analysis. Int J Implant Dent. 2019;5:43.  53. Lieber R, Pandis N, Faggion CM. Reporting and handling of incomplete outcome data in implant dentistry: A survey of randomized clinical trials. J Clin Periodontol. 2020;47:257–66.  54. Cairo F, Barbato L, Selvaggi F, Baielli MG, Piattelli A, Chambrone L. Surgical procedures for soft tissue augmentation at implant sites. A systematic review and meta-analysis of randomized controlled trials. Clin Implant Dent Relat Res. 2019;21:1262–70.  55. Ravidà A, Majzoub J, Alassadi M, Saleh MH, Askar H, Wang H-L. Impact of Implant Length on Survival of Rough-Surface Implants in Nonaugmented Posterior Areas: A Systematic Review and Meta-Regression Analysis. Int J Oral Maxillofac Implants. 2019;34:1359–69.  56. Sun J, Eberhard J, Glage S, Held N, Voigt H, Schwabe K, et al. Development of a peri-implantitis model in the rat. Clin Oral Implants Res. 2020;31:203–14.  57. Wang C-W, Yu S-H, Mandelaris GA, Wang H-L. Is periodontal phenotype modification therapy beneficial for patients receiving orthodontic treatment? An American Academy of Periodontology best evidence review. J Periodontol. 2020;91:299–310.  58. Varvara G, Sinjari B, Caputi S, Scarano A, Piattelli M. The Relationship Between Time of Retightening and Preload Loss of Abutment Screws for Two Different Implant Designs: An In Vitro Study. J Oral Implantol. 2020;46:13–7.  59. Biesbrock A, He T, DiGennaro J, Zou Y, Ramsey D, Garcia-Godoy F. The effects of bioavailable gluconate chelated stannous fluoride dentifrice on gingival bleeding: Meta-analysis of eighteen randomized controlled trials. J Clin Periodontol. 2019;46:1205–16.  60. Ma M, Qi M, Zhang D, Liu H. The Clinical Performance of Narrow Diameter Implants Versus Regular Diameter Implants: A Meta-Analysis. J Oral Implantol. 2019;45:503–8.  61. Monje A, Chappuis V, Monje F, Muñoz F, Wang H-L, Urban IA, et al. The Critical Peri-implant Buccal Bone Wall Thickness Revisited: An Experimental Study in the Beagle Dog. Int J Oral Maxillofac Implants. 2019;34:1328–36.  62. Al-Moraissi EA, Oginni FO, Mahyoub Holkom MA, Mohamed AAS, Al-Sharani HM. Tissue-engineered bone using mesenchymal stem cells versus conventional bone grafts in the regeneration of maxillary alveolar bone: A systematic review and meta-analysis. Int J Oral Maxillofac Implants. 2020;35:79–90.  63. Al Harthi SM, Prihoda TJ, Mealey BL, Lasho DJ, Noujeim M, Huynh-Ba G. Healing at Molar Extraction Sites Using Freeze-Dried Bone Allograft and Collagen Wound Dressing: Case Series and Three-Arm Analyses. Int J Oral Maxillofac Implants. 2019;34:1202–12.  64. Wen S-C, Huang W-X, Wang H-L. Regeneration of Peri-implantitis Infrabony Defects: Report on Three Cases. Int J Periodontics Restorative Dent. 2019;39:615–21.  65. Creeth J, Maclure R, Seong J, Gomez-Pereira P, Budhawant C, Sufi F, et al. Three randomized studies of dentine hypersensitivity reduction after short-term SnF2 toothpaste use. J Clin Periodontol. 2019;46:1105–15.  66. Novello S, Debouche A, Philippe M, Naudet F, Jeanne S. Clinical application of mesenchymal stem cells in periodontal regeneration: A systematic review and meta-analysis. J Periodontal Res. 2020;55:1–12.  67. Cortellini P, Cortellini S, Tonetti MS. Papilla preservation flaps for periodontal regeneration of molars severely compromised by combined furcation and intrabony defects: Retrospective analysis of a registry-based cohort. J Periodontol. 2020;91:165–73.  68. Chambrone L, Ortega MAS, Sukekava F, Rotundo R, Kalemaj Z, Buti J, et al. Root coverage procedures for treating single and multiple recession-type defects: An updated Cochrane systematic review. J Periodontol. 2019;90:1399–422.  69. Thoma DS, Jung U-W, Gil A, Kim MJ, Paeng K-W, Jung RE, et al. The effects of hard and soft tissue grafting and individualization of healing abutments at immediate implants: an experimental study in dogs. J Periodontal Implant Sci. 2019;49:171–84.  70. Ravidà A, Wang I-C, Sammartino G, Barootchi S, Tattan M, Troiano G, et al. Prosthetic Rehabilitation of the Posterior Atrophic Maxilla, Short (≤6 mm) or Long (≥10 mm) Dental Implants? A Systematic Review, Meta-analysis, and Trial Sequential Analysis: Naples Consensus Report Working Group A. Implant Dent. 2019;28:590–602.  71. Nibali L, Koidou VP, Hamborg T, Donos N. Empirical or microbiologically guided systemic antimicrobials as adjuncts to non-surgical periodontal therapy? A systematic review. J Clin Periodontol. 2019;46:999–1012.  72. Sarkis-Onofre R, Marchini L, Spazzin AO, Santos MBFD. Randomized Controlled Trials in Implant Dentistry: Assessment of the Last 20 Years of Contribution and Research Network Analysis. J Oral Implantol. 2019;45:327–33.  73. de Camargo L, da Silva SN, Chambrone L. Efficacy of toothbrushing procedures performed in intensive care units in reducing the risk of ventilator-associated pneumonia: A systematic review. J Periodontal Res. 2019;54:601–11.  74. Chen S, Ou Q, Lin X, Wang Y. Comparison Between a Computer-Aided Surgical Template and the Free-Hand Method: A Systematic Review and Meta-Analysis. Implant Dent. 2019;28:578–89.  75. Gargallo-Albiol J, Barootchi S, Tavelli L, Wang H-L. Efficacy of Xenogeneic Collagen Matrix to Augment Peri-Implant Soft Tissue Thickness Compared to Autogenous Connective Tissue Graft: A Systematic Review and Meta-Analysis. Int J Oral Maxillofac Implants. 2019;34:1059–69.  76. Lin C-Y, Chen Z, Pan W-L, Wang H-L. Effect of Platelet-Rich Fibrin on Ridge Preservation in Perspective of Bone Healing: A Systematic Review and Meta-analysis. Int J Oral Maxillofac Implants. 2019;34:845–54.  77. Sanz-Martín I, Cha J-K, Yoon S-W, Sanz-Sánchez I, Jung U-W. Long-term assessment of periodontal disease progression after surgical or non-surgical treatment: a systematic review. J Periodontal Implant Sci. 2019;49:60–75.  78. Sendyk DI, Rovai ES, Souza NV, Deboni MCZ, Pannuti CM. Selective outcome reporting in randomized clinical trials of dental implants. J Clin Periodontol. 2019;46:758–65.  79. Gargallo-Albiol J, Sinjab KH, Barootchi S, Chan H-L, Wang H-L. Microscope and micro-camera assessment of Schneiderian membrane perforation via transcrestal sinus floor elevation: A randomized ex vivo study. Clin Oral Implants Res. 2019;30:682–90.  80. Figuero E, Herrera D, Tobías A, Serrano J, Roldán S, Escribano M, et al. Efficacy of adjunctive anti-plaque chemical agents in managing gingivitis: A systematic review and network meta-analyses. J Clin Periodontol. 2019;46:723–39.  81. Bitaraf T, Keshtkar A, Rokn AR, Monzavi A, Geramy A, Hashemi K. Comparing short dental implant and standard dental implant in terms of marginal bone level changes: A systematic review and meta-analysis of randomized controlled trials. Clin Implant Dent Relat Res. 2019;21:796–812.  82. Gupta S, Del Fabbro M, Chang J. The impact of simvastatin intervention on the healing of bone, soft tissue, and TMJ cartilage in dentistry: a systematic review and meta-analysis. Int J Implant Dent. 2019;5:17.  83. Schneider D, Sancho-Puchades M, Schober F, Thoma D, Hämmerle C, Jung R. A Randomized Controlled Clinical Trial Comparing Conventional and Computer-Assisted Implant Planning and Placement in Partially Edentulous Patients. Part 3: Time and Cost Analyses. Int J Periodontics Restorative Dent. 2019;39:e71–82.  84. Dai A, Huang J-P, Ding P-H, Chen L-L. Long-term stability of root coverage procedures for single gingival recessions: A systematic review and meta-analysis. J Clin Periodontol. 2019;46:572–85.  85. Serino G, Hultin K. Periimplant Disease and Prosthetic Risk Indicators: A Literature Review. Implant Dent. 2019;28:125–37.  86. Ravidà A, Barootchi S, Alkanderi A, Tavelli L, Suárez-López Del Amo F. The Effect of Crown-to-Implant Ratio on the Clinical Outcomes of Dental Implants: A Systematic Review. Int J Oral Maxillofac Implants. 2019;34:1121–31.  87. Jawad S, Clarke PT. Survival of Mini Dental Implants Used to Retain Mandibular Complete Overdentures: Systematic Review. Int J Oral Maxillofac Implants. 2019;34:343–56.  88. Susin C, Finger Stadler A, Musskopf ML, de Sousa Rabelo M, Ramos UD, Fiorini T. Safety and efficacy of a novel, gradually anodized dental implant surface: A study in Yucatan mini pigs. Clin Implant Dent Relat Res. 2019;21 Suppl 1:44–54.  89. Susin C, Finger Stadler A, Fiorini T, de Sousa Rabelo M, Ramos UD, Schüpbach P. Safety and efficacy of a novel anodized abutment on soft tissue healing in Yucatan mini-pigs. Clin Implant Dent Relat Res. 2019;21 Suppl 1:34–43.  90. Dos Santos MBF, Agostini BA, de Moraes RR, Schwendicke F, Sarkis-Onofre R. Industry sponsorship bias in clinical trials in implant dentistry: Systematic review and meta-regression. J Clin Periodontol. 2019;46:510–9.  91. Romero-Millán J J, Aizcorbe-Vicente J, Peñarrocha-Diago M, Galindo-Moreno P, Canullo L, Peñarrocha-Oltra D. Implants in the Posterior Maxilla: Open Sinus Lift Versus Conventional Implant Placement. A Systematic Review. Int J Oral Maxillofac Implants. 2019;34:e65–76.  92. Toniazzo MP, Nodari D, Muniz FWMG, Weidlich P. Effect of mHealth in improving oral hygiene: A systematic review with meta-analysis. J Clin Periodontol. 2019;46:297–309.  93. Pauletto P, Ruales-Carrera E, Gonçalves TMSV, Philippi AG, Donos N, Mezzomo LA. Fixed and Removable Full-Arch Restorations Supported by Short (≤ 8-mm) Dental Implants In the Mandible: A Systematic Review and Meta-Analysis. Int J Oral Maxillofac Implants. 2019;34:873–85.  94. Dank A, Aartman IHA, Wismeijer D, Tahmaseb A. Effect of dental implant surface roughness in patients with a history of periodontal disease: a systematic review and meta-analysis. Int J Implant Dent. 2019;5:12.  95. Romandini M, De Tullio I, Congedi F, Kalemaj Z, D’Ambrosio M, Laforí A, et al. Antibiotic prophylaxis at dental implant placement: Which is the best protocol? A systematic review and network meta-analysis. J Clin Periodontol. 2019;46:382–95.  96. Cooper LF, Reside G, Stanford C, Barwacz C, Feine J, Nader SA, et al. Three-Year Prospective Randomized Comparative Assessment of Anterior Maxillary Single Implants with Different Abutment Interfaces. Int J Oral Maxillofac Implants. 2019;34:150–8.  97. Thoma DS, Bienz SP, Figuero E, Jung RE, Sanz-Martín I. Efficacy of lateral bone augmentation performed simultaneously with dental implant placement: A systematic review and meta-analysis. J Clin Periodontol. 2019;46 Suppl 21:257–76.  98. Hasuike A, Ueno D, Nagashima H, Kubota T, Tsukune N, Watanabe N, et al. Methodological quality and risk-of-bias assessments in systematic reviews of treatments for peri-implantitis. J Periodontal Res. 2019;54:374–87.  99. Urban IA, Montero E, Monje A, Sanz-Sánchez I. Effectiveness of vertical ridge augmentation interventions: A systematic review and meta-analysis. J Clin Periodontol. 2019;46 Suppl 21:319–39.  100. Chye RML, Perrotti V, Piattelli A, Iaculli F, Quaranta A. Effectiveness of Different Commercial Chlorhexidine-Based Mouthwashes After Periodontal and Implant Surgery: A Systematic Review. Implant Dent. 2019;28:74–85.  101. Ramanauskaite A, Obreja K, Sader R, Khoury F, Romanos G, Koo KT, et al. Surgical Treatment of Periimplantitis With Augmentative Techniques. Implant Dent. 2019;28:187–209.  102. Cosyn J, De Lat L, Seyssens L, Doornewaard R, Deschepper E, Vervaeke S. The effectiveness of immediate implant placement for single tooth replacement compared to delayed implant placement: A systematic review and meta-analysis. J Clin Periodontol. 2019;46 Suppl 21:224–41.  103. Naenni N, Lim H-C, Papageorgiou SN, Hämmerle CHF. Efficacy of lateral bone augmentation prior to implant placement: A systematic review and meta-analysis. J Clin Periodontol. 2019;46 Suppl 21:287–306.  104. Avila-Ortiz G, Chambrone L, Vignoletti F. Effect of alveolar ridge preservation interventions following tooth extraction: A systematic review and meta-analysis. J Clin Periodontol. 2019;46 Suppl 21:195–223.  105. El Kholy K, Janner SFM, Schimmel M, Buser D. The influence of guided sleeve height, drilling distance, and drilling key length on the accuracy of static Computer-Assisted Implant Surgery. Clin Implant Dent Relat Res. 2019;21:101–7.  106. Keeve PL, Koo KT, Ramanauskaite A, Romanos G, Schwarz F, Sculean A, et al. Surgical Treatment of Periimplantitis With Non-Augmentative Techniques. Implant Dent. 2019;28:177–86.  107. Messias A, Nicolau P, Guerra F. Titanium dental implants with different collar design and surface modifications: A systematic review on survival rates and marginal bone levels. Clin Oral Implants Res. 2019;30:20–48.  108. Gargallo-Albiol J, Tattan M, Sinjab KH, Chan H-L, Wang H-L. Schneiderian membrane perforation via transcrestal sinus floor elevation: A randomized ex vivo study with endoscopic validation. Clin Oral Implants Res. 2019;30:11–9.  109. Levin L, Barbu H, Kurgan S, Comăneanu RM, Referendaru D, Lorean A. Evaluation of 0.2% delmopinol mouth rinse for prevention of peri-implant mucositis and peri-implantitis: A randomized controlled canine study. Clin Implant Dent Relat Res. 2019;21:46–51.  110. Yao W, Shah B, Chan H-L, Wang H-L, Lin G-H. Bone Quality and Quantity Alterations After Socket Augmentation with rhPDGF-BB or BMPs: A Systematic Review. Int J Oral Maxillofac Implants. 2018;33:1255–65.  111. Ravidà A, Wang I-C, Barootchi S, Askar H, Tavelli L, Gargallo-Albiol J, et al. Meta-analysis of randomized clinical trials comparing clinical and patient-reported outcomes between extra-short (≤6 mm) and longer (≥10 mm) implants. J Clin Periodontol. 2019;46:118–42.  112. Jokstad A, Ganeles J. Systematic review of clinical and patient-reported outcomes following oral rehabilitation on dental implants with a tapered compared to a non-tapered implant design. Clin Oral Implants Res. 2018;29 Suppl 16:41–54.  113. Papaspyridakos P, De Souza A, Vazouras K, Gholami H, Pagni S, Weber H-P. Survival rates of short dental implants (≤6 mm) compared with implants longer than 6 mm in posterior jaw areas: A meta-analysis. Clin Oral Implants Res. 2018;29 Suppl 16:8–20.  114. Huynh-Ba G, Oates TW, Williams MAH. Immediate loading vs. early/conventional loading of immediately placed implants in partially edentulous patients from the patients’ perspective: A systematic review. Clin Oral Implants Res. 2018;29 Suppl 16:255–69.  115. Yao CJ, Cao C, Bornstein MM, Mattheos N. Patient-reported outcome measures of edentulous patients restored with implant-supported removable and fixed prostheses: A systematic review. Clin Oral Implants Res. 2018;29 Suppl 16:241–54.  116. Gallucci GO, Hamilton A, Zhou W, Buser D, Chen S. Implant placement and loading protocols in partially edentulous patients: A systematic review. Clin Oral Implants Res. 2018;29 Suppl 16:106–34.  117. Tahmaseb A, Wu V, Wismeijer D, Coucke W, Evans C. The accuracy of static computer-aided implant surgery: A systematic review and meta-analysis. Clin Oral Implants Res. 2018;29 Suppl 16:416–35.  118. Pjetursson BE, Valente NA, Strasding M, Zwahlen M, Liu S, Sailer I. A systematic review of the survival and complication rates of zirconia-ceramic and metal-ceramic single crowns. Clin Oral Implants Res. 2018;29 Suppl 16:199–214.  119. Jung RE, Al-Nawas B, Araujo M, Avila-Ortiz G, Barter S, Brodala N, et al. Group 1 ITI Consensus Report: The influence of implant length and design and medications on clinical and patient-reported outcomes. Clin Oral Implants Res. 2018;29 Suppl 16:69–77.  120. Sailer I, Strasding M, Valente NA, Zwahlen M, Liu S, Pjetursson BE. A systematic review of the survival and complication rates of zirconia-ceramic and metal-ceramic multiple-unit fixed dental prostheses. Clin Oral Implants Res. 2018;29 Suppl 16:184–98.  121. Salvi GE, Monje A, Tomasi C. Long-term biological complications of dental implants placed either in pristine or in augmented sites: A systematic review and meta-analysis. Clin Oral Implants Res. 2018;29 Suppl 16:294–310.  122. Strauss FJ, Stähli A, Gruber R. The use of platelet-rich fibrin to enhance the outcomes of implant therapy: A systematic review. Clin Oral Implants Res. 2018;29 Suppl 18:6–19.  123. Stavropoulos A, Bertl K, Pietschmann P, Pandis N, Schiødt M, Klinge B. The effect of antiresorptive drugs on implant therapy: Systematic review and meta-analysis. Clin Oral Implants Res. 2018;29 Suppl 18:54–92.  124. Stähli A, Strauss FJ, Gruber R. The use of platelet-rich plasma to enhance the outcomes of implant therapy: A systematic review. Clin Oral Implants Res. 2018;29 Suppl 18:20–36.  125. Pjetursson BE, Zarauz C, Strasding M, Sailer I, Zwahlen M, Zembic A. A systematic review of the influence of the implant-abutment connection on the clinical outcomes of ceramic and metal implant abutments supporting fixed implant reconstructions. Clin Oral Implants Res. 2018;29 Suppl 18:160–83.  126. Merli M, Moscatelli M, Pagliaro U, Mariotti G, Merli I, Nieri M. Implant prosthetic rehabilitation in partially edentulous patients with bone atrophy. An umbrella review based on systematic reviews of randomised controlled trials. Eur J Oral Implantol. 2018;11:261–80.  127. Chambrone L, Wang H-L, Romanos GE. Antimicrobial photodynamic therapy for the treatment of periodontitis and peri-implantitis: An American Academy of Periodontology best evidence review. J Periodontol. 2018;89:783–803.  128. Lin G-H, Suárez López Del Amo F, Wang H-L. Laser therapy for treatment of peri-implant mucositis and peri-implantitis: An American Academy of Periodontology best evidence review. J Periodontol. 2018;89:766–82.  129. da Silva JC, Muniz FWMG, Oballe HJR, Andrades M, Rösing CK, Cavagni J. The effect of periodontal therapy on oxidative stress biomarkers: A systematic review. J Clin Periodontol. 2018;45:1222–37.  130. Tolentino da Rosa de Souza P, Binhame Albini Martini M, Reis Azevedo-Alanis L. Do short implants have similar survival rates compared to standard implants in posterior single crown?: A systematic review and meta-analysis. Clin Implant Dent Relat Res. 2018;20:890–901.  131. Aludden H, Mordenfeld A, Hallman M, Christensen A-E, Starch-Jensen T. Osteotome-Mediated Sinus Floor Elevation With or Without a Grafting Material: A Systematic Review and Meta-analysis of Long-term Studies (≥5-Years). Implant Dent. 2018;27:488–97.  132. Sánchez-Pérez A, Cano-Tovar AB, Martín-de-Llano JJ, Sarobe-Oyarzun FJ, Davis S, Carda-Batalla C. Effect of Rotary Instrument Mineral Oil Lubricant on Osseointegration: A Randomized, Blinded Study in Rabbits. J Oral Implantol. 2019;45:12–7.  133. Freitas da Silva EV, Dos Santos DM, Sonego MV, de Luna Gomes JM, Pellizzer EP, Goiato MC. Does the Presence of a Cantilever Influence the Survival and Success of Partial Implant-Supported Dental Prostheses? Systematic Review and Meta-Analysis. Int J Oral Maxillofac Implants. 2018;33:815–23.  134. Cho Y-D, Ku Y. Guided bone regeneration using K-incision technique. J Periodontal Implant Sci. 2018;48:193–200.  135. Niu W, Wang P, Ge S, Ji P. Effects of Platelet Concentrates Used in Alveolar Ridge Preservation: A Systematic Review. Implant Dent. 2018;27:498–506.  136. Sanz-Sánchez I, Sanz-Martín I, Carrillo de Albornoz A, Figuero E, Sanz M. Biological effect of the abutment material on the stability of peri-implant marginal bone levels: A systematic review and meta-analysis. Clin Oral Implants Res. 2018;29 Suppl 18:124–44.  137. Gatti F, Gatti C, Tallarico M, Tommasato G, Meloni SM, Chiapasco M. Maxillary Sinus Membrane Elevation Using a Special Drilling System and Hydraulic Pressure: A 2-Year Prospective Cohort Study. Int J Periodontics Restorative Dent. 2018;38:593–9.  138. Dreyer H, Grischke J, Tiede C, Eberhard J, Schweitzer A, Toikkanen SE, et al. Epidemiology and risk factors of peri-implantitis: A systematic review. J Periodontal Res. 2018;53:657–81.  139. Ghanaati S, Herrera-Vizcaino C, Al-Maawi S, Lorenz J, Miron RJ, Nelson K, et al. Fifteen Years of Platelet Rich Fibrin in Dentistry and Oromaxillofacial Surgery: How High is the Level of Scientific Evidence? J Oral Implantol. 2018;44:471–92.  140. Alfadda SA. Current Evidence on Dental Implants Outcomes in Smokers and Nonsmokers: A Systematic Review and Meta-Analysis. J Oral Implantol. 2018;44:390–9.  141. Perić M, Cavalier E, Toma S, Lasserre JF. Serum vitamin D levels and chronic periodontitis in adult, Caucasian population-a systematic review. J Periodontal Res. 2018;53:645–56.  142. Akram Z, Vohra F, Javed F. Locally delivered metformin as adjunct to scaling and root planing in the treatment of periodontal defects: A systematic review and meta-analysis. J Periodontal Res. 2018;53:941–9.  143. Starch-Jensen T, Mordenfeld A, Becktor JP, Jensen SS. Maxillary Sinus Floor Augmentation With Synthetic Bone Substitutes Compared With Other Grafting Materials: A Systematic Review and Meta-analysis. Implant Dent. 2018;27:363–74.  144. Mercado F, Hamlet S, Ivanovski S. Regenerative surgical therapy for peri-implantitis using deproteinized bovine bone mineral with 10% collagen, enamel matrix derivative and Doxycycline-A prospective 3-year cohort study. Clin Oral Implants Res. 2018;29:583–91.  145. Antonoglou GN, Stavropoulos A, Samara MD, Ioannidis A, Benic GI, Papageorgiou SN, et al. Clinical Performance of Dental Implants Following Sinus Floor Augmentation: A Systematic Review and Meta-Analysis of Clinical Trials with at Least 3 Years of Follow-up. Int J Oral Maxillofac Implants. 2018;33:e45–65.  146. Elnayef B, Porta C, Suárez-López Del Amo F, Mordini L, Gargallo-Albiol J, Hernández-Alfaro F. The Fate of Lateral Ridge Augmentation: A Systematic Review and Meta-Analysis. Int J Oral Maxillofac Implants. 2018;33:622–35.  147. Lee J, Lee J-B, Koo K-T, Seol Y-J, Lee Y-M. Flap Management in Alveolar Ridge Preservation: A Systematic Review and Meta-Analysis. Int J Oral Maxillofac Implants. 2018;33:613–21.  148. Caricasulo R, Malchiodi L, Ghensi P, Fantozzi G, Cucchi A. The influence of implant-abutment connection to peri-implant bone loss: A systematic review and meta-analysis. Clin Implant Dent Relat Res. 2018;20:653–64.  149. Atieh MA, Alsabeeha N, Duncan WJ. Stability of tapered and parallel-walled dental implants: A systematic review and meta-analysis. Clin Implant Dent Relat Res. 2018;20:634–45.  150. Tavelli L, Barootchi S, Nguyen TVN, Tattan M, Ravidà A, Wang H-L. Efficacy of tunnel technique in the treatment of localized and multiple gingival recessions: A systematic review and meta-analysis. J Periodontol. 2018;89:1075–90.  151. Susin C, Lee J, Fiorini T, de Freitas RM, Chiu H-C, Prasad HS, et al. Sinus augmentation using rhBMP-2/ACS in a mini-pig model: Influence of an adjunctive ceramic bone biomaterial. J Clin Periodontol. 2018;45:1005–13.  152. Chambrone L, Ramos UD, Reynolds MA. Infrared lasers for the treatment of moderate to severe periodontitis: An American Academy of Periodontology best evidence review. J Periodontol. 2018;89:743–65.  153. Farronato D, Fumagalli D, Asa’ad F, Rasperini G. Decontamination of Customized Laser-Microtextured Titanium Abutments: A Comparative in Vitro Study of Different Cleaning Procedures. Int J Periodontics Restorative Dent. 2018;38:e87–95.  154. Susin C, Lee J, Fiorini T, Koo K-T, Schüpbach P, Angst PDM, et al. Screening of candidate biomaterials for alveolar augmentation using a critical-size rat calvaria defect model. J Clin Periodontol. 2018;45:884–93.  155. Lin C-Y, Chen Z, Pan W-L, Wang H-L. Impact of timing on soft tissue augmentation during implant treatment: A systematic review and meta-analysis. Clin Oral Implants Res. 2018;29:508–21.  156. Homma S, Makabe Y, Sakai T, Morinaga K, Yokoue S, Kido H, et al. Prospective multicenter non-randomized controlled study on intraosseous stability and healing period for dental implants in the posterior region. Int J Implant Dent. 2018;4:10.  157. Sendyk DI, de Oliveira NK, Pannuti CM, da Graça Naclério-Homem M, Wennerberg A, Deboni MCZ. Conventional Drilling Versus Piezosurgery for Implant Site Preparation: A Meta-Analysis. J Oral Implantol. 2018;44:400–5.  158. Faggion CM, Monje A, Wasiak J. Appraisal of systematic reviews on the management of peri-implant diseases with two methodological tools. J Clin Periodontol. 2018;45:754–66.  159. Troiano G, Lo Russo L, Canullo L, Ciavarella D, Lo Muzio L, Laino L. Early and late implant failure of submerged versus non-submerged implant healing: A systematic review, meta-analysis and trial sequential analysis. J Clin Periodontol. 2018;45:613–23.  160. Kotsakis GA, Lian Q, Ioannou AL, Michalowicz BS, John MT, Chu H. A network meta-analysis of interproximal oral hygiene methods in the reduction of clinical indices of inflammation. J Periodontol. 2018;89:558–70.  161. Sanz-Sánchez I, Carrillo de Albornoz A, Figuero E, Schwarz F, Jung R, Sanz M, et al. Effects of lateral bone augmentation procedures on peri-implant health or disease: A systematic review and meta-analysis. Clin Oral Implants Res. 2018;29 Suppl 15:18–31.  162. Pinto JPNS, Goergen J, Muniz FWMG, Haas AN. Vitamin D levels and risk for periodontal disease: A systematic review. J Periodontal Res. 2018;53:298–305.  163. Kroll P, Hou L, Radaideh H, Sharifi N, Han PP, Mulligan R, et al. Oral Health-Related Outcomes in Edentulous Patients Treated With Mandibular Implant-Retained Dentures Versus Complete Dentures: Systematic Review With Meta-Analyses. J Oral Implantol. 2018;44:313–24.  164. Jordi C, Mukaddam K, Lambrecht JT, Kühl S. Membrane perforation rate in lateral maxillary sinus floor augmentation using conventional rotating instruments and piezoelectric device-a meta-analysis. Int J Implant Dent. 2018;4:3.  165. Moro MG, Silveira Souto ML, Franco GCN, Holzhausen M, Pannuti CM. Efficacy of local phytotherapy in the nonsurgical treatment of periodontal disease: A systematic review. J Periodontal Res. 2018;53:288–97.  166. Dini C, Pereira MMA, Souza JGS, Shibli JA, de Avila ED, Barão VAR. Association between industry support and the reporting of study outcomes in randomized clinical trials of dental implant research from the past 20 years. Clin Implant Dent Relat Res. 2022;24:94–104.  167. Anitua E, Anitua B, Alkhraisat MH, Piñas L, Eguia A, Torre A. Dental implants survival after nasal floor elevation: a systematic review. J Oral Implantol. 2021. https://doi.org/10.1563/aaid-joi-D-21-00219.  168. Baumeister S-E, Reckelkamm SL, Baurecht H, Nolde M, Kocher T, Holtfreter B, et al. A Mendelian randomization study on the effect of 25-hydroxyvitamin D levels on periodontitis. J Periodontol. 2021. https://doi.org/10.1002/JPER.21-0463.  169. Wu DT, Raoof S, Latimer JM, Nguyen TT. Partial Extraction Therapy: A Review of Human Clinical Studies. J Oral Implantol. 2021. https://doi.org/10.1563/aaid-joi-D-21-00095.  170. Al-Maawi S, Becker K, Schwarz F, Sader R, Ghanaati S. Efficacy of platelet-rich fibrin in promoting the healing of extraction sockets: a systematic review. Int J Implant Dent. 2021;7:117.  171. de Melo Menezes K, Roncalli da Costa Oliveira ÂG, de Vasconcelos Gurgel BC. Impact of 0.12% Chlorhexidine Gluconate Mouthwash on Peri-Implant Mucositis and Gingivitis After Nonsurgical Treatment: A Multilevel Analysis. Int J Oral Maxillofac Implants. 2021;36:1188–97.  172. Gaspar J, Proença L, Botelho J, Machado V, Chambrone L, Neiva R, et al. Implant Stability of Osseodensification Drilling Versus Conventional Surgical Technique: A Systematic Review. Int J Oral Maxillofac Implants. 2021;36:1104–10.  173. Dommisch H, Walter C, Difloe-Geisert JC, Gintaute A, Jepsen S, Zitzmann NU. Efficacy of tooth splinting and occlusal adjustment in patients with periodontitis exhibiting masticatory dysfunction: A systematic review. J Clin Periodontol. 2021. https://doi.org/10.1111/jcpe.13563.  174. Orlandi M, Muñoz Aguilera E, Marletta D, Petrie A, Suvan J, D’Aiuto F. Impact of the treatment of periodontitis on systemic health and quality of life: A systematic review. J Clin Periodontol. 2021. https://doi.org/10.1111/jcpe.13554.  175. Montero E, Molina A, Palombo D, Morón B, Pradíes G, Sanz-Sánchez I. Efficacy and risks of tooth-supported prostheses in the treatment of partially edentulous patients with stage IV periodontitis. A systematic review and meta-analysis. J Clin Periodontol. 2021. https://doi.org/10.1111/jcpe.13482.  176. Ramanauskaite A, Fretwurst T, Schwarz F. Efficacy of alternative or adjunctive measures to conventional non-surgical and surgical treatment of peri-implant mucositis and peri-implantitis: a systematic review and meta-analysis. Int J Implant Dent. 2021;7:112.  177. Mao Z, Lee C-T, He SM, Zhang S, Bao J, Xie ZG. Buccal bone dimensional changes at immediate implant sites in the maxillary esthetic zone within a 4-12-month follow-up period: A systematic review and meta-analysis. Clin Implant Dent Relat Res. 2021;23:883–903.  178. Tomasi C, Albouy J-P, Schaller D, Navarro RC, Derks J. Efficacy of rehabilitation of stage IV periodontitis patients with full-arch fixed prostheses: Tooth-supported versus Implant-supported-A systematic review. J Clin Periodontol. 2021. https://doi.org/10.1111/jcpe.13511.  179. Kloukos D, Roccuzzo A, Stähli A, Sculean A, Katsaros C, Salvi GE. Effect of combined periodontal and orthodontic treatment of tilted molars and of teeth with intra-bony and furcation defects in stage-IV periodontitis patients: A systematic review. J Clin Periodontol. 2021. https://doi.org/10.1111/jcpe.13509.  180. Shi J-Y, Zhang X, Qian S-J, Wei S-M, Yan K-X, Xu M, et al. Evidence and risk indicators of non-random sampling in clinical trials in implant dentistry: A systematic appraisal. J Clin Periodontol. 2022;49:144–52.  181. Tan NCP, Khan A, Antunes E, Miller CM, Sharma D. The effects of physical decontamination methods on zirconia implant surfaces: a systematic review. J Periodontal Implant Sci. 2021;51:298–315.  182. Mishra SK, Gaddale R, Sonnahalli NK, Chowdhary R. Platform-Switching Concept in Dental Implants: A Systematic Review and Meta-analysis of Randomized Controlled Trials with a Minimum Follow-up of 3 Years. Int J Oral Maxillofac Implants. 2021;36:e97–109.  183. de Moura Costa PV, Ferreira MS, Veríssimo C, de Torres ÉM, Valladares-Neto J, Garcia Silva MA. Is Zirconia Better Than Titanium Abutments for Soft Tissue Color? A Systematic Review and Meta-analysis of Spectrophotometric Evaluation. Int J Oral Maxillofac Implants. 2021;36:875–84.  184. Sugita R, Jones AA, Kotsakis GA, Cochran DL. Radiographic evaluation of a novel bone adhesive for maintenance of crestal bone around implants in canine oversized osteotomies. J Periodontol. 2021. https://doi.org/10.1002/JPER.20-0876.  185. Sanz-Martín I, Cha J-K, Sanz-Sánchez I, Figuero E, Herrera D, Sanz M. Changes in peri-implant soft tissue levels following surgical treatment of peri-implantitis: A systematic review and meta-analysis. Clin Oral Implants Res. 2021;32 Suppl 21:230–44.  186. Gotfredsen K, Hosseini M, Rimborg S, Özhayat E. Patient’s perception of timing concepts in implant dentistry: A systematic review. Clin Oral Implants Res. 2021;32 Suppl 21:67–84.  187. Pjetursson BE, Sailer I, Latyshev A, Rabel K, Kohal R-J, Karasan D. A systematic review and meta-analysis evaluating the survival, the failure, and the complication rates of veneered and monolithic all-ceramic implant-supported single crowns. Clin Oral Implants Res. 2021;32 Suppl 21:254–88.  188. Aiquel LL, Pitta J, Antonoglou GN, Mischak I, Sailer I, Payer M. Does the timing of implant placement and loading influence biological outcomes of implant-supported multiple-unit fixed dental prosthesis-A systematic review with meta-analyses. Clin Oral Implants Res. 2021;32 Suppl 21:5–27.  189. Francisco H, Marques D, Pinto C, Aiquel L, Caramês J. Is the timing of implant placement and loading influencing esthetic outcomes in single-tooth implants?-A systematic review. Clin Oral Implants Res. 2021;32 Suppl 21:28–55.  190. Gao X, Qin S, Cai H, Wan Q. Comparison of general and aesthetic effects between flapless and flap techniques in dental implantation: a meta-analysis of randomized controlled trials. Int J Implant Dent. 2021;7:100.  191. Camacho-Alonso F, Salinas J, Sánchez-Siles M, Pato-Mourelo J, Cotrina-Veizaga BD, Ortega N. Synergistic antimicrobial effect of photodynamic therapy and chitosan on the titanium-adherent biofilms of Staphylococcus aureus, Escherichia coli, and Pseudomonas aeruginosa: An in vitro study. J Periodontol. 2021. https://doi.org/10.1002/JPER.21-0306.  192. Menne MC, Pandis N, Faggion CM. Reporting quality of abstracts of randomized controlled trials related to implant dentistry. J Periodontol. 2021;:73–82.  193. Totou D, Naka O, Mehta SB, Banerji S. Esthetic, mechanical, and biological outcomes of various implant abutments for single-tooth replacement in the anterior region: a systematic review of the literature. Int J Implant Dent. 2021;7:85.  194. Badaró MM, Mendoza Marin DO, Pauletto P, Simek Vega Gonçalves TM, Porporatti AL, De Luca Canto G. Failures in Single Extra-Short Implants (≤ 6 mm): A Systematic Review and Meta-analysis. Int J Oral Maxillofac Implants. 2021;36:669–89.  195. Dini C, Borges GA, Costa RC, Magno MB, Maia LC, Barão VAR. Peri-implant and esthetic outcomes of cemented and screw-retained crowns using zirconia abutments in single implant-supported restorations-A systematic review and meta-analysis. Clin Oral Implants Res. 2021;32:1143–58.  196. Nagay BE, Dini C, Borges GA, Mesquita MF, Cavalcanti YW, Magno MB, et al. Clinical efficacy of anodized dental implants for implant-supported prostheses after different loading protocols: A systematic review and meta-analysis. Clin Oral Implants Res. 2021;32:1021–40.  197. Nitta K, Yoneyama M. Polymer concentration dependence of the helix to random coil transition of a charged polypeptide in aqueous salt solution. Biophys Chem. 1975;3:323–9.  198. Van de Winkel T, Heijens L, Listl S, Meijer G. What is the evidence on the added value of implant‐supported overdentures? A review. Clin Implant Dent Rel Res. 2021;23:644–56.  199. Zaki J, Yusuf N, El-Khadem A, Scholten RJPM, Jenniskens K. Efficacy of bone-substitute materials use in immediate dental implant placement: A systematic review and meta-analysis. Clin Implant Dent Relat Res. 2021;23:506–19.  200. Blase DV, Dricot RG, Lasserre JF, Toma S, Brecx MC. Combination of a Hydraulic Device and Nanohydroxylapatite Paste for Minimally Invasive Transcrestal Sinus Floor Elevation: Procedure and 4-Year Results. Int J Oral Maxillofac Implants. 2021;36:587–97.  201. Jiang X, Zhou W, Wu Y, Wang F. Clinical Outcomes of Immediate Implant Loading with Fixed Prostheses in Edentulous Maxillae: A Systematic Review. Int J Oral Maxillofac Implants. 2021;36:503–19.  202. Nomeika D, Jasiunas A, Janužis G, Skrodenienė E, Banienė R, Juodžbalys G. Effects of Different Bone Substitutes on Reactive Oxygen Species Release in Leukocytes in Vitro: A Pilot Study. Int J Oral Maxillofac Implants. 2021;36:e42–50.  203. Zhu M, Zhao M, Hu B, Wang Y, Li Y, Song J. Efficacy of glycine powder air-polishing in supportive periodontal therapy: a systematic review and meta-analysis. J Periodontal Implant Sci. 2021;51:147–62.  204. Rosenberg SL. Temperature and pH optima for 21 species of thermophilic and thermotolerant fungi. Can J Microbiol. 1975;21:1535–40.  205. Ma G, Wu C, Shao M. Simultaneous implant placement with autogenous onlay bone grafts: a systematic review and meta-analysis. Int J Implant Dent. 2021;7:61.  206. Lopez-Lopez J, Ramezanzade S, Atabaki MS, Sadeghi E. Psychologic Status and Influence of Surgery Techniques on Acute Nonneuropathic Pain After Dental Implant Surgery: Systematic Review and Meta-analyses. Int J Oral Maxillofac Implants. 2021;36:248–69.  207. Caetano V da S, de Andrade RSB, França LF de C, Pessoa LDS, Rodrigues AA, Alves EHP, et al. Food restriction reduces hepatic alterations associated with experimental periodontitis. J Periodontol. 2022;93:156–65.  208. Saravi B, Vollmer A, Lang G, Adolphs N, Li Z, Giers V, et al. Impact of renin-angiotensin system inhibitors and beta-blockers on dental implant stability. Int J Implant Dent. 2021;7:31.  209. Atieh MA, Baqain ZH, Tawse-Smith A, Ma S, Almoselli M, Lin L, et al. The influence of insertion torque values on the failure and complication rates of dental implants: A systematic review and meta-analysis. Clin Implant Dent Relat Res. 2021;23:341–60.  210. Reiniger APP, Maier J, Wikesjö UME, Moreira CHC, Kantorski KZ. Correlation between dental plaque accumulation and gingival health in periodontal maintenance patients using short or extended personal oral hygiene intervals. J Clin Periodontol. 2021;48:834–42.  211. Sendyk DI, Souza NV, César Neto JB, Tatakis DN, Pannuti CM. Selective outcome reporting in root coverage randomized clinical trials. J Clin Periodontol. 2021;48:867–77.  212. Melo JGA, Sousa JP, Firmino RT, Matins CC, Granville-Garcia AF, Nonaka CFW, et al. Different applications forms of green tea (Camellia sinensis (L.) Kuntze) for the treatment of periodontitis: a systematic review and meta-analysis. J Periodontal Res. 2021;56:443–53.  213. AlMoharib HS, Steffensen B, Zoukhri D, Finkelman M, Gyurko R. Efficacy of an Er:YAG laser in the decontamination of dental implant surfaces: An in vitro study. J Periodontol. 2021;92:1613–21.  214. López-Pacheco A, Soto-Peñaloza D, Gómez M, Peñarrocha-Oltra D, Alarcón MA. Socket seal surgery techniques in the esthetic zone: a systematic review with meta-analysis and trial sequential analysis of randomized clinical trials. Int J Implant Dent. 2021;7:13.  215. Alshamsi M, Mehta J, Nibali L. Study design and primary outcome in randomized controlled trials in periodontology. A systematic review. J Clin Periodontol. 2021;48:859–66.  216. Wei S-M, Zhu Y, Wei J-X, Zhang C-N, Shi J-Y, Lai H-C. Accuracy of dynamic navigation in implant surgery: A systematic review and meta-analysis. Clin Oral Implants Res. 2021;32:383–93.  217. Aimetti M, Fratini A, Manavella V, Giraudi M, Citterio F, Ferrarotti F, et al. Pocket resolution in regenerative treatment of intrabony defects with papilla preservation techniques: A systematic review and meta-analysis of randomized clinical trials. J Clin Periodontol. 2021;48:843–58.  218. Toledano-Serrabona J, Romeu-I-Fontanet A, Gay-Escoda C, Camps-Font O, Sánchez-Garcés MÁ. Clinical and histological outcomes of maxillary sinus floor augmentation with synthetic bone substitutes for dental implant treatment: A meta-analysis. J Oral Implantol. 2021. https://doi.org/10.1563/aaid-joi-D-20-00202.  219. Bilgin Çetin M, Sezgin Y, Maraş E, Cebeci İA. Association of probable bruxism with periodontal status: A cross-sectional study in patients seeking periodontal care. J Periodontal Res. 2021;56:370–8.  220. Sgolastra F, Petrucci A, Ciarrocchi I, Masci C, Spadaro A. Adjunctive systemic antimicrobials in the treatment of chronic periodontitis: A systematic review and network meta-analysis. J Periodontal Res. 2021;56:236–48.  221. Stavropoulos A, Bertl K, Spineli LM, Sculean A, Cortellini P, Tonetti M. Medium- and long-term clinical benefits of periodontal regenerative/reconstructive procedures in intrabony defects: Systematic review and network meta-analysis of randomized controlled clinical studies. J Clin Periodontol. 2021;48:410–30.  222. Trimmel B, Gede N, Hegyi P, Szakács Z, Mezey GA, Varga E, et al. Relative performance of various biomaterials used for maxillary sinus augmentation: A Bayesian network meta-analysis. Clin Oral Implants Res. 2021;32:135–53.  223. Merchant AT, Liu J, Reynolds MA, Beck JD, Zhang J. Quantile regression to estimate the survivor average causal effect of periodontal treatment effects on birthweight and gestational age. J Periodontol. 2021;92:975–82.  224. Nakayama Y, Matsuda H, Itoh S, Iwai Y, Takai H, Mezawa M, et al. Impact of adjunctive procedures on recombinant human fibroblast growth factor-2-mediated periodontal regeneration therapy: A retrospective study. J Periodontol. 2021;92:983–94.  225. Seyssens L, De Lat L, Cosyn J. Immediate implant placement with or without connective tissue graft: A systematic review and meta-analysis. J Clin Periodontol. 2021;48:284–301.  226. Li S, Zhang T, Zhou M, Zhang X, Gao Y, Cai X. A novel digital and visualized guided bone regeneration procedure and digital precise bone augmentation: A case series. Clin Implant Dent Relat Res. 2021;23:19–30.  227. Zhao P, Wang Q, Zhang P, Zhou X, Nie L, Liang X, et al. Clinical Efficacy of Chlorhexidine as an Adjunct to Mechanical Therapy of Peri-Implant Disease: A Systematic Review and Meta-Analysis. J Oral Implantol. 2021;47:78–87.  228. Li J, Chen Z, Mendonça G, Chan H-L, Sinjab K, Wang H-L. Potential of Using an Implant Fixture as a Ridge Expander for Minor Ridge Augmentation: An Ex Vivo Randomized Controlled Study. J Oral Implantol. 2021;47:125–30.  229. Ferreira DM, Knorst JK, Menegazzo GR, Bolsson GB, Ardenghi TM. Effect of individual and neighborhood social capital on gingival bleeding in children: A 7-year cohort study. J Periodontol. 2021;92:1430–40. | 1. Review 2. Retrospective study 3. Systematic review and meta-analysis 4. Systematic review 5. Systematic review and meta-analysis 6. Case series 7. Non-randomised trial 8. Systematic review 9. Systematic 10. Quasi-randomised trial 11. Cohort study 12. Systematic review and meta-analysis 13. Literature review 14. In-vitro study 15. Review 16. Retrospective study 17. Systematic review and meta-analysis 18. Review 19. Systematic review and meta-analysis 20. Systematic review and meta-analysis 21. Systematic review and meta-analysis 22. Cross-sectional study 23. Posteriori additional analysis of rct 24. Systematic review 25. Case series 26. Systematic review and meta-analysis 27. Animal study 28. Network meta-analysis 29. Analysis of side effects in RCT 30. Animal study 31. Observational study 32. Systematic review and meta-analysis 33. Non-randomised trial 34. Systematic review and meta-analysis 35. Systematic review and emta-analysis 36. Meta-analysis 37. Systematic-Review and meta-analysis 38. Systematic review and meta-analysis 39. Systematic review and meta-analysis 40. Systematic Review 41. Systematic Review 42. Systematic review and meta-analysis 43. Systematic Review 44. Systematic review and meta-analysis 45. Review 46. Systematic review 47. Systematic review and meta-analysis 48. Systematic review and meta-analysis 49. Systematic review and meta-analysis 50. Systematic review and meta analysis 51. Systematic review 52. Systematic review and meta-analysis 53. Meta-research study 54. Systematic review and meta-analysis 55. Systematic review and meta-analysis 56. Animal study 57. Review 58. In vitro study 59. Meta-analysis 60. meta-analysis 61. Animal study 62. Systematic review and meta-analysis 63. Case series 64. Case series 65. Multiple RCT analysis 66. Systemtic review and meta-analysis 67. Retrospective study 68. Systematic review 69. Animal study 70. Systematic review and meta-analysis 71. Systematic review 72. Review 73. Systematic review 74. Systematic review and meta-analysis 75. Systematic review and Meta-analysis 76. Systematic review and meta-analysis 77. Systematic review 78. Review 79. Ex-vivo study 80. Systematic review and meta-analysis 81. Systematic review and meta-analysis 82. Systematic review and meta-analysis 83. Additional time and cost analysis of RCT 84. Systematic review and meta-analysis 85. Literature review 86. Systematic review 87. Systematic review 88. Animal study 89. Animal study 90. Systematic review and meta regression 91. Systematic review 92. Systematic review and meta-anyslsis 93. Systematic review and meta-analysis 94. Systematic review and meta-analysis 95. Systematic review and meta-analysis 96. Comparative study 97. Systematic review and meta-analysis 98. Methodology study 99. Systematic review and meta-analysis   100. Systematic review  101. Review  102. Systematic review and meta-analysis  103. Systematic review and meta-analysis  104.Systematic review and meta-analysis  105.No human RCT  106. Review  107. Systematic review and meta-anylsis  108. ex vivo study  109. Anaimal study  110. Systematic review  111.Meta-analysis  112. Systematic review  113.Meta-analysis  114. Systematic review  115.Systematic review  116. Systematic review  117. Systemaic Review and meta-analysis  118. Systematic review  119. Consensus report  120. Systematic review  121. Systematic review and meta-analyis  122. Systematic review  123. Systematic review and meta-analysis  124. Systematic review  125. Systematic review  126. Systermatic review  127. Review  128. review  129. Systematic review  130. Systematic review and meta-analysis  131. Systematic review and meta-analysis  132. Animal study  133. Systematic review and meta-analysis  134. Review  135. Systematic review  136. Systematic review and meta-analysis  137. Prospective cohort study  138. Systematic review  139. Review  140. Systematic review and Meta-analysis  141. Systematic review  142.Systematic review and meta-analysis  143. Systematic review and meta-analysis  144.Prospective cohort study  145. Systematic review and meta-analysis  146. Systematic review and meta-analysis  147. Systematic review and meta-analysis  148. Systematic review and meta-analysis  149. Systematic review and meta-analysis  150. Systematic review and meta-analysis  151. Animal study  152. Review  153. In vitro study  154. Animal study  155. Systematic review and meta-analysis  156. non-randomised trial  157. meta-analysis  158. Methodology study  159. Systematic review and meta-analysis  160. Meta analysis  161. Systematic review and meta-analysis  162. Systematic review  163. Systematic review and meta-analysis  164. meta-analysis  165. Systematic review  166. Review  167. Systematic Review  168. Mendelian randomization study  169. Review  170. Systematic review  171. Multilevel analysis  172. Systematic review  173. Systematic review  174. Systematic review  175. Systematic review and meta-analysis  176. Systematic review and meta-analysis  177. Systematic review and meta-analysis  178. Systematic review  179. Systematic Review  180. Systematic appraisal  181. Systematic review  182. Systematic review and network meta-analysis  183. Systematic review and meta-analysis  184. Animal study  185. Systematic review and meta-analysis  186. Systematic review  187. Systematic review and meta-analysis  188. Systematic review and meta-analysis  189. Systematic review  190. Meta analysis  191. In vitro study  192. meta-research study  193. Systematic review  194. Systematic review and meta-analysis  195. Systematic review and meta-analysis  196. Systematic review and meta-analysis  197. Non human trial (dental material)  198. Review  199. Systematic review and meta-analysis  200. Retrospective study  201. Systematic review  202. In vitro study  203. Systematic review and meta-analysis  204. Non dentistry journal  205. Systematic review and meta-analysis  206. Systematic review and meta-analysis  207. Animal study  208. Retrospective  209. Systematic review and meta-analysis  210. Secondary analysis of RCT  211. Review  212. Systematic review and meta-analysis  213. In vitro study  214. Systematic review and meta-analysis  215. Systematic Review  216. Systematic review and meta-analysis  217. Systematic review and meta-analysis  218. Meta-analysis  219. Cross-sectional study  220. Systematic review and meta-analysis  221. Systematic review and meta-analysis  222. Meta-analysis  223. Quantile regression  224. Retrospective study  225. Systematic review and meta-analysis  226. Case seriees  227. Systematic review and meta-analysis  228. ex vivo study  229. Cohort study |
