# Supplementary material for: Reporting of flow diagrams in randomised controlled trials published in periodontology and implantology: a survey
Source: BMC Med Res Methodol. 2023 Apr 27;23:105. doi: 10.1186/s12874-023-01923-7 (PMC10134555; doi:10.1186/s12874-023-01923-7)
Supplement: Supplementary file 5 — Additional file 5. Articles excluded after full text assessment with reasons. [file 12874_2023_1923_MOESM5_ESM.docx]

**Additional file 5, Articles excluded after full text assessment with reasons**

| Article | Reason for exclusion |
| --- | --- |
| 1. Cieplik F, Ihlenfeld I, Hiller K-A, Pummer A, Schmalz G, Buchalla W, et al. Tooth survival and clinical outcomes up to 26 years after guided tissue regeneration therapy in deep intra-bony defects: Follow-up investigation of three randomized clinical trials. J Clin Periodontol. 2020;47:863–74.  2. Hagenfeld D, Prior K, Harks I, Jockel-Schneider Y, May TW, Harmsen D, et al. No differences in microbiome changes between anti-adhesive and antibacterial ingredients in toothpastes during periodontal therapy. J Periodontal Res. 2019;54:435–43.  3. Esposito M, González-García A, Peñarrocha Diago M, Fernández Encinas R, Trullenque-Eriksson A, Xhanari E, et al. Natural or palatal positioning of immediate post-extractive implants in the aesthetic zone? 1-year results of a multicentre randomised controlled trial. Eur J Oral Implantol. 2018;11:189–200.  4. Lu W, Xu J, Wang H-M, He F-M. Influence of Lateral Windows with Decreased Vertical Height Following Maxillary Sinus Floor Augmentation: A 1-year Clinical and Radiographic Study. Int J Oral Maxillofac Implants. 2018;33:661–70.  5. Calon TGA, van Tongeren J, Omar O, Johansson ML, Stokroos R-J. Cytokine expression profile in the bone-anchored hearing system: 12-week results from a prospective randomized, controlled study. Clin Implant Dent Relat Res. 2018;20:606–16.  6. Clementini M, Castelluzzo W, Ciaravino V, Agostinelli A, Vignoletti F, Ambrosi A, et al. The effect of immediate implant placement on alveolar ridge preservation compared to spontaneous healing after tooth extraction: Soft tissue findings from a randomized controlled clinical trial. J Clin Periodontol. 2020;47:1536–46.  7. Aldana CA, Ruiz AS, Messina DR, Quirynen M, Carrasco NP. Leukocyte- and Platelet-Rich Fibrin Versus Connective Tissue Graft for a Coronally Advanced Flap in the Treatment of Miller Class I and II Localized Gingival Recessions: A Randomized Controlled Clinical Trial. Int J Periodontics Restorative Dent. 2021;41:e287–96.  8. Karcı B, Öncü E, Dogan M. The Effect of Different Dental Implant Surface Characteristics on Bone Immunologic Biomarkers and Microbiologic Parameters: A Randomized Clinical Study. Int J Periodontics Restorative Dent. 2021;41:589–97. | 1. Follow-up of 3 RCTs 2. Subgroup analysis of an RCT 3. No full text, requested via email but authors did not answer 4. Retrospective results of an RCT 5. Not related to dentistry, RCT from Otorhinolaryngology 6. Secondary report of an RCT 7. No full text, requested via email but authors did not answer 8. No full text, requested via email but authors did not answer |
