# Supplementary material for: Reporting of flow diagrams in randomised controlled trials published in periodontology and implantology: a survey
Source: BMC Med Res Methodol. 2023 Apr 27;23:105. doi: 10.1186/s12874-023-01923-7 (PMC10134555; doi:10.1186/s12874-023-01923-7)
Supplement: Supplementary file 6 — Additional file 6. Completeness of reporting per item in the flow diagram (N=317). [file 12874_2023_1923_MOESM6_ESM.docx]

**Additional file 6** Completeness of reporting per item in the flow diagram (N=317)

| Variable | Not reported  N (%) | Reported  N (%) |
| --- | --- | --- |
| 1) Eligibility | 40 (12.6) | 277 (87.4) |
| 2) Excluded overall | 44 (13.9) | 273 (86.1) |
| 3) Exclusion criteria | 54 (17.0) | 263 (83.0) |
| 4) Declined participation | 54 (17.0) | 263 (83.0) |
| 5) Excluded other reasons | 54 (17.0) | 263 (83.0) |
| 6) Randomised | 5 (1.6) | 312 (98.4) |
| 7) Allocated | 7 (2.2) | 310 (97.8) |
| 8) Received intervention | 7 (2.2) | 310 (97.8) |
| 9) Did not receive intervention | 7 (2.2) | 310 (97.8) |
| 10) Reasons did not receive intervention | 13 (4.1) | 304 (95.9) |
| 11) Lost to follow-up | 11 (3.5) | 306 (96.5) |
| 12) Reasons lost to follow-up | 96 (30.3) | 221 (69.7) |
| 13) Discontinued intervention | 11 (3.5) | 306 (96.5) |
| 14) Reasons discontinued intervention | 18 (5.7) | 299 (94.3) |
| 15) Included main analysis | 25 (7.9) | 292 (92.1) |
| 16) Excluded main analysis | 25 (7.9) | 292 (92.1) |
| 17) Reasons excluded analysis | 43 (13.6) | 274 (86.4) |
